# A New Species of the Basal “Kangaroo” *Balbaroo* and a Re-Evaluation of Stem Macropodiform Interrelationships

**DOI:** 10.1371/journal.pone.0112705

**Published:** 2014-11-19

**Authors:** Karen H. Black, Kenny J. Travouillon, Wendy Den Boer, Benjamin P. Kear, Bernard N. Cooke, Michael Archer

**Affiliations:** 1 School of Biological, Earth and Environmental Sciences, University of New South Wales, Sydney, Australia; 2 School of Earth Sciences, University of Queensland, St Lucia, Australia; 3 Palaeobiology Programme, Department of Earth Sciences, Uppsala University, Uppsala, Sweden; 4 Queensland Museum, South Brisbane, Australia; Monash University, Australia

## Abstract

Exceptionally well-preserved skulls and postcranial elements of a new species of the plesiomorphic stem macropodiform *Balbaroo* have been recovered from middle Miocene freshwater limestone deposits in the Riversleigh World Heritage Area of northwestern Queensland, Australia. This constitutes the richest intraspecific sample for any currently known basal “kangaroo”, and, along with additional material referred to *Balbaroo fangaroo*, provides new insights into structural variability within the most prolific archaic macropodiform clade – Balbaridae. Qualitative and metric evaluations of taxonomic boundaries demonstrate that the previously distinct species *Nambaroo bullockensis* is a junior synonym of *B. camfieldensis*. Furthermore, coupled Maximum Parsimony and Bayesian phylogenetic analyses reveal that our new *Balbaroo* remains represent the most derived member of the *Balbaroo* lineage, and are closely related to the middle Miocene *B. camfieldensis*, which like most named balbarid species is identifiable only from isolated jaws. The postcranial elements of *Balbaroo* concur with earlier finds of the stratigraphically oldest balbarid skeleton, *Nambaroo gillespieae*, and suggest that quadrupedal progression was a primary gait mode as opposed to bipedal saltation. All *Balbaroo* spp. have low-crowned bilophodont molars, which are typical for browsing herbivores inhabiting the densely forested environments envisaged for middle Miocene northeastern Australia.

## Introduction

Balbaridae is an extinct radiation of dentally lophodont macropodiforms – an iconic clade of diprotodontian marsupials that today includes the prolific kangaroos (Macropodidae) and rat-kangaroos (Potoroidae). Balbaridae was originally envisaged by Flannery, Archer and Plane [1] as a subfamilial grouping – Balbarinae – that was possibly ancestral to macropodids, but subsequently elevated to familial status by Cooke and Kear [2], based on a phylogenetic analysis [3] that recovered Balbaridae as a discrete monophyletic clade basal to all other macropodiforms.

Thirteen species of balbarids are presently ascribed to at least five genera: *Balbaroo* (3 spp.), *Nambaroo* [4] (5 spp.), *Ganawamaya* [5] (3 spp.), *Wururoo* [6] (1 spp.), and *Galanarla* [1] (1 sp.). Their fossils occur in various deposits from central South Australia to northeastern Queensland and the Northern Territory, and stratigraphically range from the late Oligocene to middle Miocene [7], [8]. In addition, as many as seven presently undescribed species of *Nambaroo* have been identified from the famous Riversleigh World Heritage Area in northwestern Queensland [8], [9], and a further three undefined balbarids are reported from the Etadunna Formation of South Australia [10]. The extent to which these remains are conspecific is unknown.

Most balbarids are identified from mandibular and dental material with only two species represented by well-preserved crania: *Balbaroo fangaroo* [11], and *Nambaroo gillespieae* [12]. An articulated postcranium has also been documented for *Nambaroo gillespieae*, and thus far represents the stratigraphically oldest known for any macropodiform. Both the craniodental and postcranial skeleton of balbarids indicate a potentially unique behavioral repertoire that has not reoccurred in any subsequent macropodiform lineage. For example, *B. fangaroo* was found to possess hypertrophied upper canines that resembled ‘fangs’, a distinctive feature in comparison to the extant “true” kangaroos and rat-kangaroos (Macropodoidea). Furthermore, the appendicular elements imply heightened pedal mobility, an opposable first toe (lost in derived macropodoids), and robust forelimbs consistent with quadrupedal versus bounding progression, and perhaps the ability to climb [2], [12].

Despite this, the mainly dental data available for most balbarids has hampered a robust interpretation of their interrelationships (see [3], [7], [12], [13]). Indeed, although relatively complete cranial material is known for the type genus *Balbaroo*, this genus is still represented by only six specimens across three species. The identification of any substantial cranial, and particularly, postcranial fossils of balbarids is therefore significant because it could clarify their proposed unusual palaeoecology together with their taxonomic status as the most basal radiation within Macropodiformes. Here we describe a new species of *Balbaroo*, which is established on exceptionally well-preserved craniodental and associated postcranial material. In addition, we present novel dental specimens of *B. fangaroo* and reassess the generic assignment of balbarid material from the archetypal Bullock Creek Local Fauna of the Northern Territory, Australia.

## Materials and Methods

**Figure 1:**
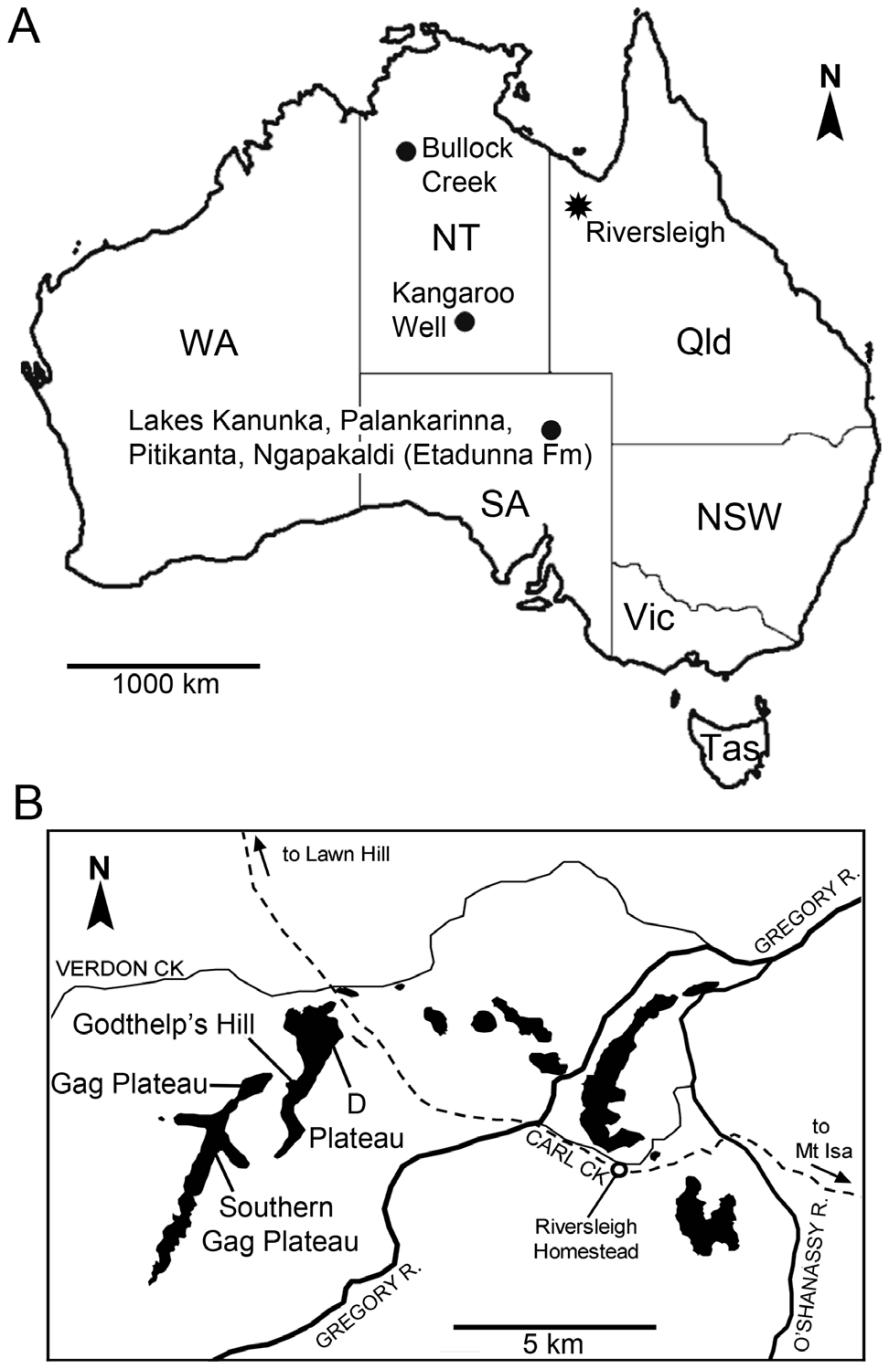
Map showing (A) Australian fossil localities discussed in the text; and (B) schematic of the Riversleigh World Heritage Area, northwestern Queensland (After Arena [31], and Megirian [94]).

Familial and subfamilial classifications followed in this work include Cooke and Kear [2], and Kear and Cooke [7], with higher-level taxonomic classification following Meredith et al. [14] in use of the subordinal clade name Macropodiformes. Dental terminology is after Archer [15]; however, the cusp in the posterolingual position on upper molars is considered to be the metaconule (*sensu* Tedford and Woodburne [16]), rather than the hypocone. Premolar and molar homology derived from Flower [17] with the exception of deciduous third premolar homology (dp3, DP3), which was identified by Luckett [18]. General cranial terminology is based on Stirton [19], Murray [20], [21], Murray et al. [22], [23], Archer [24] and Aplin [25] for basicranial features. Postcranial skeletal and myological terminology follows Flannery [26], Wells and Tedford [27], and Kear et al. [12]. Biostratigraphic nomenclature draws on Woodburne et al. [10], Archer et al. [28], [29], Creaser [30], Arena [31], and Travouillon et al. [32], [33]. Australian fossil localities mentioned in the text are indicated in Figure 1, including a schematic map of the Riversleigh World Heritage Area. All necessary permits were obtained for the described study, which complied with all relevant regulations. Permits numbers from the Queensland Government Department of Environment and Heritage Protection were WITK09501311 and EPBC2011/5925, with all specimens registered in the paleontological collections of the Queensland Museum, Brisbane, Australia.

### Institutional Abbreviations

AR, Palaeontological collection, University of New South Wales, Sydney; CPC, Commonwealth Palaeontological Collection, Canberra; NMV P, Museum Victoria Palaeontological collection, Melbourne; NTM, P, Northern Territory Museum Palaeontological collection, Alice Springs; QM F, Queensland Museum Fossil collection, Brisbane; QVM, Queen Victoria Museum, Launceston.

### Nomenclatural Acts

The electronic edition of this article conforms to the requirements of the amended International Code of Zoological Nomenclature, and hence the new names contained herein are available under that Code from the electronic edition of this article. This published work and the nomenclatural acts it contains have been registered in ZooBank, the online registration system for the ICZN. The ZooBank LSIDs (Life Science Identifiers) can be resolved and the associated information viewed through any standard web browser by appending the LSID to the prefix “http://zoobank.org/”. The LSID for this publication is: urn:lsid:zoobank.org:pub: FBE39CDD-0A58-4E04-8B0E-610D2D5F4EB9. The electronic edition of this work was published in a journal with an ISSN, and has been archived and is available from the following digital repositories: PubMed Central, LOCKSS.

### Skeletal metrics

**Figure 2:**
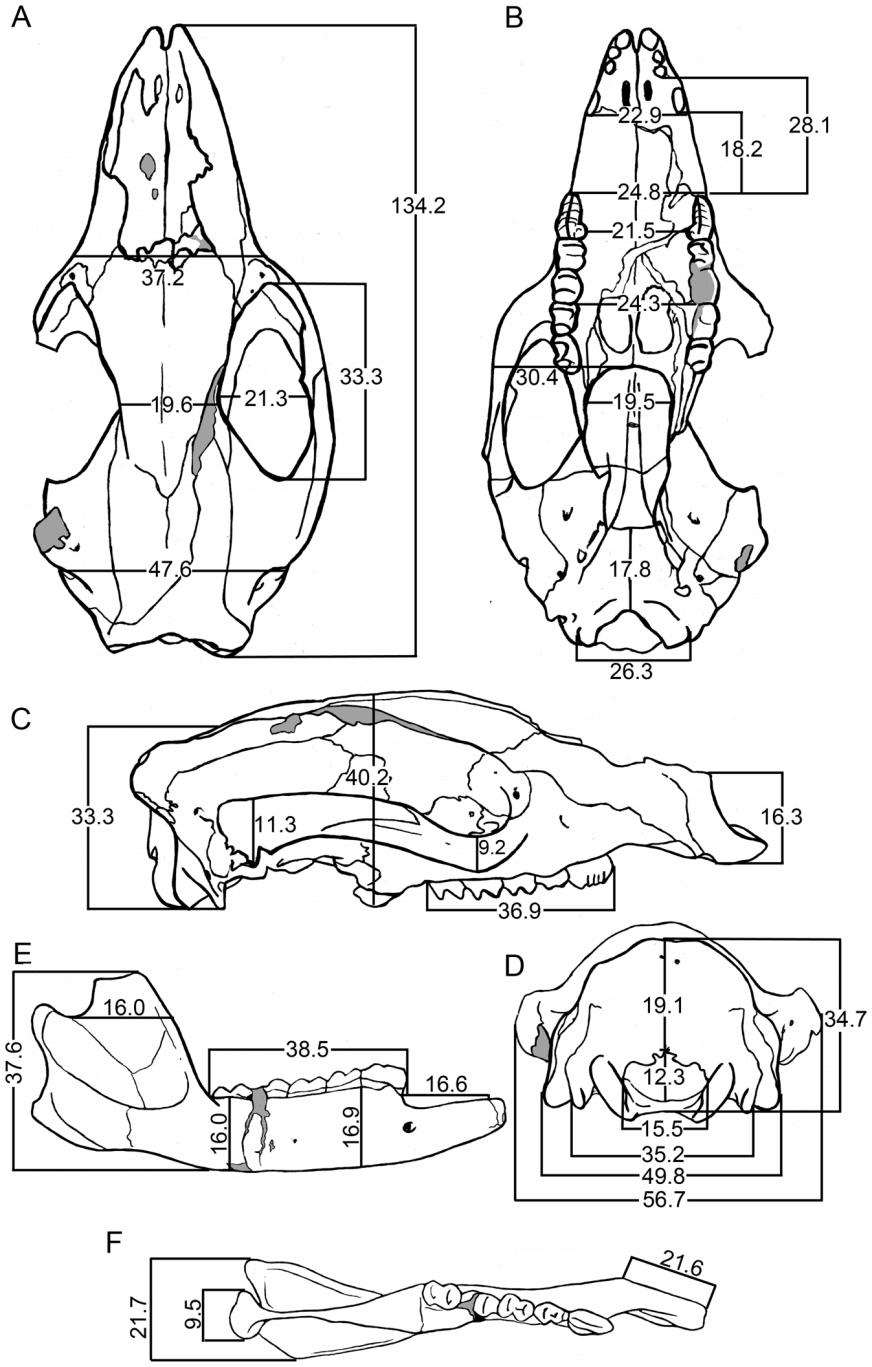
Cranio-mandibular measurements (mm) of *Balbaroo nalima* sp. nov.: (A–D) Holotype, QM F36295; (E–F) paratype, QM F31446. Not to scale.

**Figure 3:**
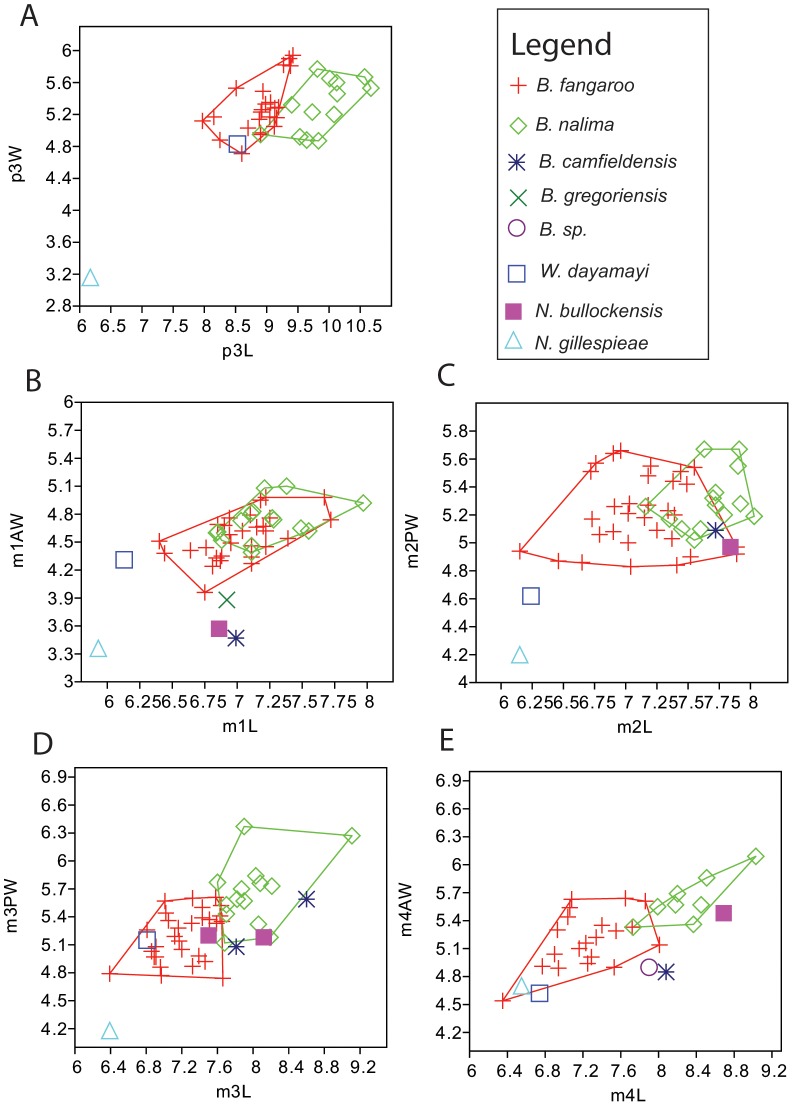
Bivariate plots of lower dentition of *Balbaroo nalima* sp. nov., *B. fangaroo, B. camfieldensis, B. gregoriensis, B.* sp., *Nambaroo bullockensis* and *N. gillespieae.* L – anteroposterior length, AW =  anterior width, W =  maximum width, P =  premolar, M =  molar.

**Figure 4:**
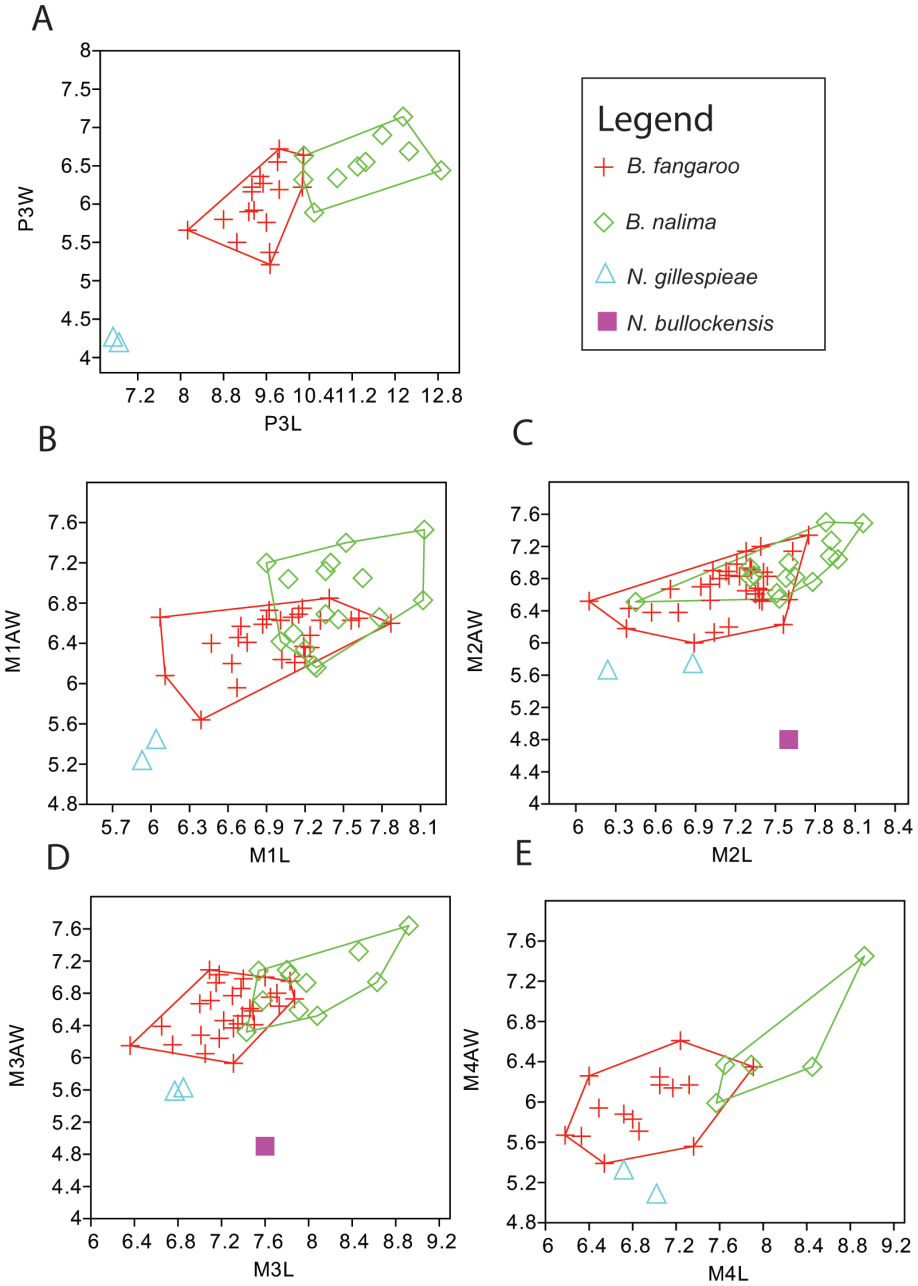
Bivariate plots of upper dentition of *Balbaroo nalima* sp. nov., *B. fangaroo, Nambaroo bullockensis* and *N. gillespieae.* L – anteroposterior length, AW =  anterior width, PW =  posterior width, W =  maximum width, p =  premolar, m =  molar.

Dental, cranio-mandibular and postcranial measurements were made using digital vernier calipers. Molar and premolar length (i.e., maximum anteroposterior dimension) and premolar width (i.e., maximum buccolingual dimension) were taken at the base of the crown. Molar anterior and posterior widths were taken across the anterior and posterior lophs/lophids, respectively. Measurements of the lower and upper dentitions from our new species of *Balbaroo* are listed in Table 1 and Table 2, respectively, and cranio-mandibular measurements are detailed in Figure 2. Dental measurements of type and additional specimens of *Balbaroo fangaroo* are given in the Supporting Information: Table S1 (lower dentition) and Table S2 (upper dentition). Basic univariate statistics and coefficients of variation were calculated for *Balbaroo nalima* n. sp. and *B. fangaroo* dental variables using PAST Version 1.51 [34] and are provided in Table S3 and Table S4, respectively. Coefficients of variation (CVs) between 4 and 10 were accepted as compatible with data set derivation from a single population, while CVs <4 implied insufficient sample size [35]. Bivariate plots (Figs 3–4) comparing dental variables of all *Balbaroo* species and some *Nambaroo gillespieae*, *Nambaroo bullockensis* and *Wururoo dayamayi*, were generated in PAST. Measurements for *Balbaroo camfieldensis, Balbaroo gregoriensis, Balbaroo* sp. (of Flannery, Archer and Plane [1]) and *N. bullockensis* were taken directly from Flannery, Archer and Plane [1] (Table S5). Postcranial element dimensions for *B. nalima* n.sp. are listed in Table S6.

**Table 1:** Measurements (mm) of the lower dentition of type and referred material of *Balbaroo nalima* sp. nov. from the Riversleigh World Heritage Area, Australia.

		dp3		p3		m1			m2			m3			m4		
QM	Site	L	W	L	W	L	AW	PW	L	AW	PW	L	AW	PW	L	AW	PW
F56964	AL90			9.64	4.88	7.49	4.65	5.08	8.03	5.49	5.19						
F50415	AL90					7.38	5.1	5.3	7.72	5.38	5.36	7.7	5.62	5.53	7.73	5.33	4.85
F52811	AL90	5.61	3.93			7.11	4.39	4.85	7.55	5.05	5.02						
F50588	AL90			9.53	4.92	6.88	4.52	4.96	7.35	5.34	5.17	7.82	5.8	5.59			
F31446	AL90			9.81	5.77	7.54		5.29	7.7	5.44	5.32	7.81	5.66		8.2	5.69	5.25
F52809	AL90			9.83	4.87	7.28	4.74	5.19	7.72	5.39	5.27	8.08	5.87	5.76	8.51	5.86	5.47
F29706	COA			8.9	4.95	7,11	4.46	4.95									
F24183	COA														8.37	5.36	5.31
F20851	COA			9.73	5.23	7.27	4.76	5.16	7.79	5.55	5.2						
F56282	COA			10.08	5.2												
F19889	COA					7.21	5.08	5.61	7.91	5.82	5.67						
F24188	COA							5.01	7.6	5.14	5.1						
F56281	COA											7.9	6.51	6.37			
F56965	COA											8.19	5.8	5.18	8.45	5.57	5.29
F56283	COA			10.57	5.67												
F24220	COA											8.03	5.52	5.84			
F23205	FF			9.4	5.32	6.85	4.61	5.05	7.45	5.25	5.1	7.68	5.58	5.12	7.99	5.55	5.04
F57592	Gag	6.44	4.42			8.38	4.99	5.63	8.29	5.86	5.99						
F20502	HH			10.01	5.65	7.11	4.81	5.33	7.9	5.63	5.55						
F20518	HH					7.55	4.62	5.04									
F20417	HH			10.13	5.46												
F20378	HH			10.12	5.6												
F56280	JC				5.52	6.84	4.6	5.3	7.16	5.18	5.26	7.87	5.39	5.7	8.18	5.57	5.49
F52804	KCB					7.12	4.83	5.6	7.63	5.87	5.67	7.6	5.95	5.77			
F52805	KCB								7.92	5.44	5.28						
F56969	KCB											8.21	5.58	5.73			
F56970	KCB					7.03	4.73	5.04									
F56971	KCB											8.06	5.54	5.32			
F56972	KCB											7.9	5.74	5.57			
F56973	KCB											7.7	5.18	5.43			
F56279	RTS			10.67	5.53	7.97	4.92	5.54		5.78		9.11	6.13	6.27	9.03	6.09	5.83

Abbreviations: AW, anterior width; dp, deciduous premolar; L, length; m, molar; p, premolar; PW, posterior width. Riversleigh Site abbreviations: AL90, Alan's Ledge 1990; COA, Cleft Of Ages; FF, Fireside Favorites; Gag, Gag; HH, Henk's Hollow; JC, Jim's Carousel; KCB, Keith's Chocky Block; RTS, Ringtail.

**Table 2:** Measurements (mm) of the upper dentition of type and referred material of *Balbaroo nalima* sp. nov. from the Riversleigh World Heritage Area, Australia.

QM	Site	P2		DP3		P3		M1			M2			M3			M4		
		L	W	L	W	L	W	L	AW	PW	L	AW	PW	L	AW	PW	L	AW	PW
F36295	AL90					10.29	6.32	7.01	6.42	6.02	7.31	6.81	6.03	7.58	6.71	5.94	7.65	6.37	4.85
F36295	AL90					10.3	6.63	7.11	6.5	5.63	5.63	6.51	6.25	7.43	6.32	6.07	7.57	5.99	4.78
F57025	AL90	4.24	3.93	5.9	4.95			7.46	6.64	6.01	6.01	6.81	6.11	7.98	6.93	6.21			
F57025	AL90	4.22	4.07	5.76	4.86			7.27	6.18	5.65	5.65	6.55	6.11	7.91	6.59	5.87			
F52811	AL90											6.62	6.07						
F52811	AL90											6.84	5.45						
F56278	AL90							7.78	6.66	6.36	6.36								
F50415	AL90					10.49	5.89	8.12	6.83	6.67	6.67	6.94	6.46	7.54	7.08	5.8	7.89	6.37	4.7
F41272	AL90			6.48	5.02			7.2	6.34	5.9	5.9								
F24181	COA					11.45	6.55	7.07	7.04	6.58	6.58								
F20492	COA					12.15	7.14												
F20841	COA					10.92	6.34	7.4	7.2	6.46	6.46	7.04	6.23	8.08	6.52	5.92	8.45	6.35	5.36
F31369	COA					11.76	6.9												
F24430	COA					11.3	6.49												
F23194	COA							6.9	7.2	6.95	6.95	7.5	6.54						
F20007	COA					11.18		8.13	7.53	6.28	6.28								
F20008	COA											6.82	6.5						
F36325	COA							7.36	6.69	6.31	6.31	7.08	6.01	7.8	7.09	6.02			
F29737	COA							7.36	7.12	6.45	6.45	7	6.17						
F31602	FF							7.29	6.16	5.64	5.64								
F56284	Gag					12.86	6.44	7.65	7.05	6.53	6.53	7.27	6.79	7.83	7.03	6.32			
F56987	HH							7.71				6.76	6.16	8.63	6.94	6.01			
F56986	HH							7.99					5.93	8.46	7.32	6.1			
F20524	RRP					12.26	6.69	7.52	7.4	6.84	6.84	7.49	6.81	8.92	7.64	6.94	8.93	7.45	6.13

Abbreviations: DP, deciduous premolar; AW, anterior width; L, length; M, molar; P, premolar; PW, posterior width; W, buccal-lingual width. Riversleigh Site abbreviations: AL90, Alan's Ledge 1990; COA, Cleft Of Ages; FF, Fireside Favorites; HH, Henk's Hollow; RRP, Rackham's Rock Pile.

### Phylogenetic Analysis

To phylogenetically determine interrelationships within Balbaridae, we conducted a series of Maximum Parsimony and Bayesian multivariate analyses using Kear and Pledge [13], the most comprehensive published morphological data set of Oligocene-Miocene macropodiforms. Because our objective was to explore the distribution of basal taxa, and not critique character interpretations or clarify the contrasting topologies of living lineages (see [14], [36]), we kept the inherent taxon selections and state polarities largely intact. However, to evaluate *Balbaroo* species topology, we specifically sought out and coded (Table S7) additional original specimens of *Balbaroo fangaroo* (QM F30456, QMF36994), *Balbaroo camfieldensis* (CPC22179), and *Balbaroo gregoriensis* (CPC22186), and also incorporated new data for *Wururoo dayamayi* (QM F19820) as well as two additional stem macropodiforms to test monophyly of Hypsiprymnodontidae (see [37]): *Hypsiprymnodon bartholomaii* (scored from published cranial remains: QM F13051, QM F13052, QM F13053); and *Propleopus oscillans* (based on composite elements: SAM P18846, SAM P20815, SAM P35632, SAM P35633, SAM P35648 and supplemented by Ride et al. [38]). Parsimony analyses were performed following the general approach of Kear and Barrett [39] using PAUP* v4.0b10 [40]. Namely, most parsimonious trees (MPTs) and bootstrap frequencies were computed using the heuristic search algorithm with tree-bisection-reconnection (TBR) branch swapping (and branch lengths of zero collapsed). Bootstrap support (>70%: see [41]) was calculated using 1000 pseudoreplicates. We preferentially employed delayed transformation (DELTRAN) character state optimization for our morphological datasets (see Angnarsson and Miller [42] for discussion). Nonetheless, unequivocal synapomorphies were shared by both DELTRAN (indicated by *italic* typeface) and ACCTRAN (accelerated transformation) character state optimization methods. TreeRot.ver3 [43] was used to calculate decay (Bremer) indices for trees.

Bayesian analyses were performed using MrBayes ver.3.2 [44] using the discrete M_K_ model [45] for morphological data. To introduce rate heterogeneity between characters (see [46] for discussion), we set the rates variation parameter to “gamma” (with coding to “variable”), and also partitioned the data (*sensu* Clarke and Middleton [47]) using cranial (characters 1–11, 45, 46), mandibular (12–18, 105), dental (19–44, 47, 106–108), and postcranial (48–104) character sets (see List S1 for individual character descriptions). Note, however, that subsequent runs with non-partitioned data produced identical topologies and compatible node support. Characters were alternately treated as unordered (default), or ordered with modifications from Kear et al. [12] and Kear and Pledge [13]. The analysis comprised two simultaneous runs of four Markov Chains each: one cold and three heated (using default heating values). Trees were sampled every 100^th^ generation for five million generations. We discarded the first 500 generations from each run as burn-in and used the remaining generations to construct a majority-rule consensus tree. Bayesian Posterior Probabilities (PP) of >0.95 were deemed as strong clade support and PPs between 0.90 and 0.95 were deemed as moderate clade support (see [48]).

### Systematic Palaeontology

Macropodiformes Kirsch, Lapointe and Springer, 1997 [49]

Balbaridae (Flannery, Archer and Plane, 1983 [1]) sensu Cooke and Kear (1999) [2]

*Balbaroo* Flannery, Archer and Plane, 1983 [1]

#### Type species

*Balbaroo camfieldensis*, Flannery, Archer and Plane, 1983 [1]

#### Additional species

*Balbaroo gregoriensis*, Flannery, Archer and Plane, 1983 [1]; *Balbaroo fangaroo*, Cooke 2000 [11]; *Balbaroo nalima* sp. nov.

#### Revised Generic Diagnosis

Species of *Balbaroo* differ from other balbarids in being larger and in having: large upper canines (except *Nambaroo gillespieae*); a well-developed hypocingulid on all lower molars; and six cuspids on the occlusal crest of P3/p3. Species of *Balbaroo* also differ from *N. gillespieae* (the only other balbarid for which significant cranial and postcranial material is known) in having: more inflated frontal sinuses; weak or absent postorbital processes of the frontals; a shallow zygomatic arch; a laterally constricted cranium at the level of the postglenoid region; laterally hypertrophied mastoid processes (but less ventrally extensive than those of *Nambaroo*); large nuchal crests that more extensively overhang the occiput; smaller, less anteriorly extensive posterior palatal vacuities; and a femoral shaft with a well-defined ridge for the insertion of the m. quadratus femoris and adductor muscle complexes.

*Balbaroo camfieldensis* Flannery, Archer and Plane, 1983 [1]

*Nambaroo bullockensis* Schwartz and Megirian, 2004 [50]

(Figure 5)

**Figure 5:**
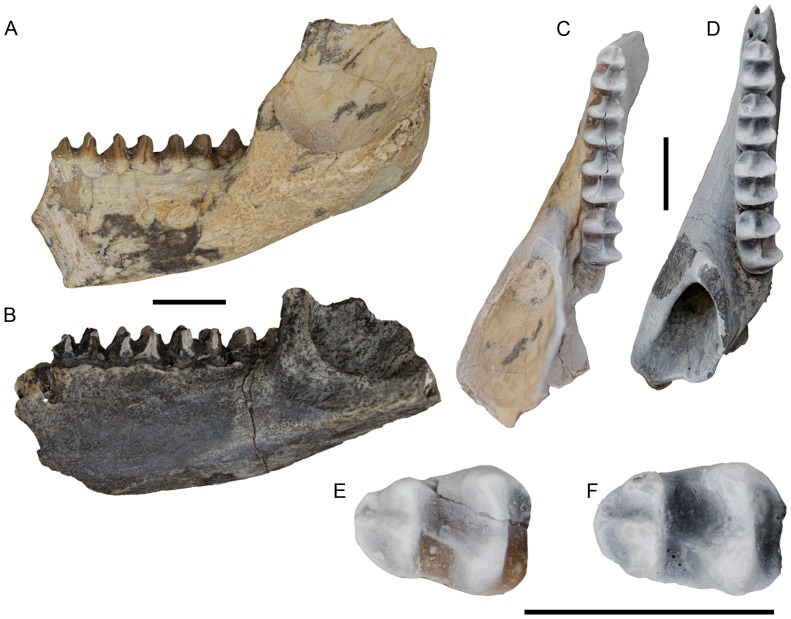
Holotypes of *Balbaroo camfieldensis* (A, C, E) (CPC 22179, left dentary) and *Nambaroo bullockensis* (B, D, F) (NTM P991-24, mirrored right dentary). (A–B) lateral view, (C–D) occlusal view, and (E–F) occlusal view of m1. Scale bars equal 10 mm.

#### Holotype

CPC 22179, posterior portion of left dentary with m1-4.

#### Material

From Horseshoe West Locality: NMV P165000, left dentary with posterior root of m1, m2-3, m4 removed from its crypt. From Blast Site: NTM P991-24, right dentary fragment with m1–4. From unrecorded quarries: QVM: 2000:GFV: 18, left m3; QVM: 2000:GFV: 67, left m2; QVM: 2000:GFV: 69, left m4 trigonid; QVM: 2000:GFV: 68, left m3 trigonid. From Top Site: NTM P9464-215, left M2; and NTM P87110-26, right M3.

#### Type Locality

Small Hills Locality, Camfield Beds, Bullock Creek Local Fauna.

#### Age and distribution

All specimens are from the Bullock Creek LF which is estimated to be middle Miocene in age [22], [23], [51]–[54].

#### Remarks

In their description of the m1(Figure 5F) of the holotype of *Nambaroo bullockensis*, Schwartz and Megirian ([50], p.669) indicate a “Swelling buccal to the protoconid is slightly indistinct due to wear but clearly represents the protostylid, joined to the protoconid by a protostylid crest”. On the basis of this feature, Schwartz and Megirian [50] assigned these specimens to the genus *Nambaroo*, species of which all possess a protostylid on m1. Schwartz and Megirian [50] further listed two synapomorphies uniting *N. bullockensis* with *Nambaroo novus*: 1, contact between the cristid obliqua and protoconid base; and 2, the fully developed hypolophid. After examining the holotype of *N. bullockensis* we could find no evidence of a protostylid on m1 (Figure 5F). Regardless, a remnant protostylid and associated buccal swelling occur in species of *Balbaroo* (e.g., *Balbaroo nalima* sp. nov.), *Wururoo* and *Ganawamaya*. Further, contact between the cristid obliqua and protoconid base occurs in species of *Balbaroo*, *Ganawamaya* and *N. gillespieae*, while a fully developed hypolophid is present in species of *Balbaroo*, *Wururoo*, *Ganawamaya* and *N. gillespieae*. We interpret the morphology of the holotype of *N. bullockensis* (Figs 5B, 5D, 5F) to be virtually identical to that of the holotype of *Balbaroo camfieldensis* (Figs 5A, 5C, 5E) and therefore regard *N. bullockensis* to be a junior synonym of *B. camfieldensis*.

*Balbaroo gregoriensis* Flannery, Archer and Plane, 1983 [1]

#### Holotype

CPC 22186, Rm1.

#### Type Locality

G Site (Verdon Creek Sequence, D Plateau) Riversleigh World Heritage Area, Lawn Hill National Park, northwestern Queensland, Australia.

#### Age and distribution

G Site is interpreted to be part of Riversleigh's Faunal Zone A, late Oligocene in age [28]–[33].

#### Remarks

Cooke [9], in his biostratigraphic analysis of Riversleigh macropodiforms, reported *Balbaroo gregoriensis* from several Faunal Zone B (then System B) deposits at Riversleigh. However, after re-examining material from these deposits, we suggest the balbarid material is instead referrable to *Balbaroo fangaroo*, based on overall morphological similarity, including the possession of a metaconid that is subequal in height to the protoconid on m1 in unworn specimens (contra the condition in *B. gregoriensis* where the protoconid is much taller than the metaconid). The only specimen known of *B. gregoriensis*, a Rm1, is most similar to that of *Wururoo dayamayi* [6], also from Riversleigh's late Oligocene FZ A. Both species possess a very low metaconid on m1 [6], in comparison to other bilophodont macropodoids. *W. dayamayi* differs from *B. gregoriensis* in being smaller and having a short protostylid crest. However, the latter feature is variably expressed in *B. fangaroo* (see below). *Wururoo dayamayi* further shares similarities in premolar morphology with *B. fangaroo* (e.g., large, plagiaulacoid and almond-shaped in occlusal view, with six cuspids, five of which having associated transcristae; [6], [11]; premolar unknown for *B. gregoriensis*). Collectively, these similaritiesraise the possibility that *W. dayamayi* may be more appropriately referred to *Balbaroo* and potentially synonymous with *B. gregoriensis.* More specimens are needed to test this proposal.

Balbaroo fangaroo Cooke, 2000 [11]

(Figure 6)

**Figure 6:**
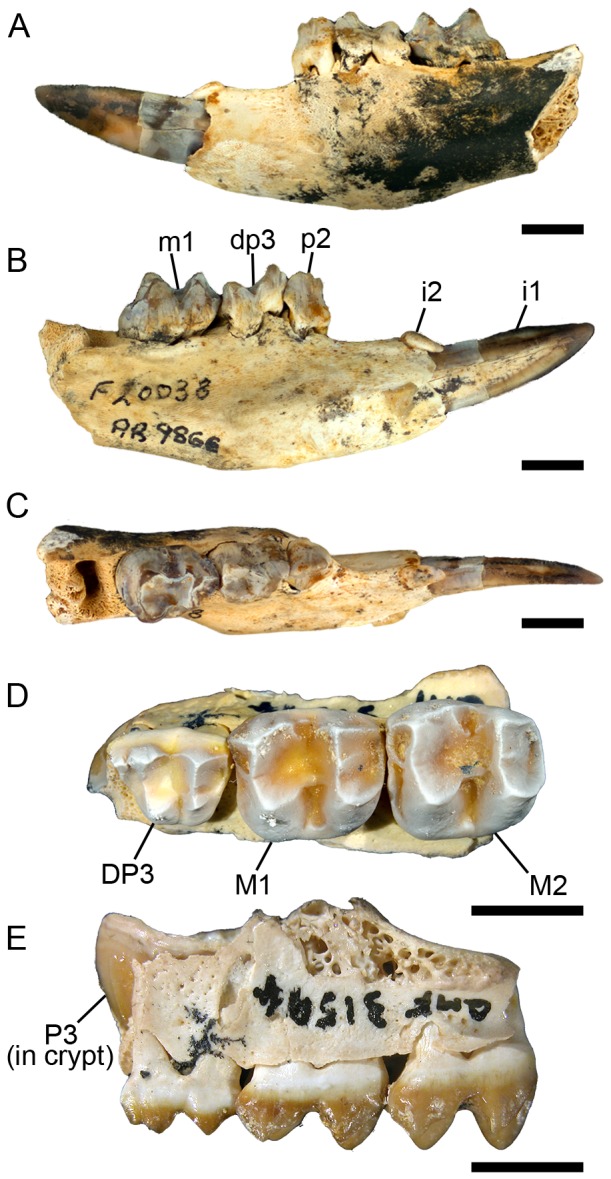
Juvenile dentition of *Balbaroo fangaroo*. (A–C), QM F20038, juvenile left dentary with i1-2, p2, dp3, m1; (D–E), QM F31594, juvenile partial left maxilla with DP3, P3 (in crypt), M1-2. (A) lateral view; (B) medial view; (C) occlusal view; (D) occlusal view; (E) lateral (buccal) view. Scale bars equal 5 mm.

#### Holotype

QM F36994, partial skull with right P3, M1-4 and associated left dentary with i1, p3, m1-4.

#### Paratype

QM F30456, anterior portion of a skull with right I1, and left and right I2-3, C1, P3, M1-4 and associated left dentary with i1, p3-m4.

#### Additional Material

All material was recovered from sites within the Riversleigh WHA, northwestern Queensland. From Boid Site: QM F20616, partial right dentary with m1-2, m3 broken. From Boid Site East: QM F56980, partial left dentary with root of i1, p3, m1. From Cadbury's Kingdom Site: QM F56291, right maxilla with M2, broken M3; QM F56292, right P3; QM F56293, left and right P3; QM F56294, right p3. From Camel Sputum Site: QM F13096, left maxilla with M2-3; QM F19608, left dentary with m3-4; QM F19638, left maxilla with M1-3; QM F19676, left maxilla with M1-3; QM F19678, right maxilla with M1-2, broken M3; QM F19697, left dentary with p3; QM F19806, left dentary with m2-4; QM F19863, left broken P3-M1, M2; QM F19865, right dentary with m3-4; QM F19867, right dentary with i1, m2-3, broken m4; QM F19971, right maxilla with M2-3; QM F19983, left maxilla with M2-4; QM F20024, left dentary with m1-4; QM F20025, left maxilla with broken P3-M1, M2-3; QM F20071, right dentary with p3, m1-2; QM F20085, left dentary with m1-4; QM F20102, left P3; QM F20162, left maxilla with M1-4; F20245, right dentary with m1 (broken); QM F20247, left dentary with m2-4; QM F20251, left dentary with m1-4; QM F20273, left M1; QM F20283, left dentary with m2-3; QM F20287, right maxilla with M2-4; QM F20314, left maxilla with M1; QM F20576, left maxilla with P3, M1-3; QM F20606, left p3; QM F23477, right maxilla with M3-4; QM F23480, right dentary with m1-2; QM F23484, left maxilla with M1-3; QM F23488, left maxilla with P3, M1-3; QM F24089, left maxilla with P3, M1-2; QM F31601, right maxilla with M1-3; QM F56288, left dentary with m1-4; QM F56974, right maxilla with P3, M1-2; QM F56975, left maxilla with P3, M1-4; QM F56976, left dentary with p3, m1-4; QM F56977, left maxilla with P3, broken M1, M2-3; QM F56978, left maxilla with P3, M1; QM F56984, left dentary with p2, dp3, m1. From Creaser's Ramparts Site: QM F20629, left dentary with p3, m1-4; QM F56287, right dentary with broken m2, m3, broken m4. From Dirk's Towers Site: QM F20367, left maxilla with M2-4; QM F20610, left dentary with p3, m1-4; QM F24524, right maxilla with M1, broken M2-3; QM F31401, right dentary with broken p3-m2, m3-4. From Inabeyance Site: QM F24518, right dentary with m1-3. From Judith's Horizontalis Site: QM F56297, left p3. From Mike's Menagerie Site: QM F20277, right m3. From Mike's Potato Patch (MPP) Site: QM F23605, left maxilla with M1-3. From Neville's Garden Site: QM F13091, left maxilla with M3-4; QM F24614, left dentary with m2-4; QM F31448, right dentary with m2-3; QM F56289, left dentary with p3, m1-4; QM F56290, right dentary with m4; QM F56983, right dentary with p3, m1. From Phil Found It Site: QM F40048, left p3. From Price Is Right Site: QM F56285, left and right dentaries with p3, m1-4; QM F56286, left dentary with m2-4. From Quantum Leap Site: QM F30427, left dentary with i1, m3, m4 in crypt.From R12C Site: QM F19671, right maxilla with M1-2. From Ross Scott Orr Site: QM F20038, juvenile left dentary with i1, p2, dp3, m1; QM F19578, left dentary with p2, dp3, m1. QM F20278, left maxilla with M3-4; QM F56296, right maxilla with M2-4. From Souvenir Site: QM F30869, right p3. From Upper Site: QM F19928, left M4; QM F19649, left maxilla with M3; QM F20087, right dentary with p3, m1-4; QM F20279, left maxilla with P3, M1-3. From Wayne's Wok Site: QM F19601, right maxilla with M3; QM F19615, right premaxilla with I1; QM F19815, left dentary with p3, broken m1; QM F19823, left dentary with m2-4; QM F19832, left dentary with unerupted m4; QM F19847, left dentary with i1, p2, dp3, p3; QM F19934, left maxilla with M1-2; QM F20005, right maxilla with DP3, P3, M1; QM F20063, right dentary with p2, dp3, unerupted p3, m1-3; QM F20073, right dentary with p3, m1-3; QM F20078, left dentary with m1; QM F24193, right maxilla with unerupted P3, M1-2; QM F31594, left maxilla with DP3 (P3), M1-2; QM F36365, right maxilla with M1-3; QM F36413, right maxilla with M1-4; QM F39979, left maxilla with P3; QM F56295, left dentary with m1, m3 in crypt; QM F56976, right dentary with p2, dp3, unerupted p3, m1-3; QM F56979, left maxilla with P3, M1-4; QM F56981, right dentary with p3, m1-3; QM F56982, right dentary with p3, m1-2; QM F56985, right dentary with unerupted p3, m1; QM F57589, right dentary with p3, m1-4; QM F57590, left dentary with broken p3, m1-4; QM F57591, left juvenile dentary with i1, p2, dp3, p3, m1-2, m3 in crypt.

#### Type Locality

The Holotype is from Outasite (Godthelp's Hill Sequence, D Plateau), and the paratype from Margo's Immense Might Site (Verdon Creek Sequence, D Plateau), Riversleigh World Heritage Area, Lawn Hill National Park, northwestern Queensland, Australia.

#### Age and distribution

Additional referred material from Boid, Boid Site East, Cadbury's Kingdom, Camel Sputum, Creaser's Ramparts, Dirk's Towers, Inabeyance, Judith's Horizontalis, Mike's Menagerie, Mike's Potato Patch, Neville's Garden, Price Is Right, Quantum Leap, Ross Scott-Orr, Upper and Wayne's Wok Sites. They are interpreted to be part of Riversleigh's Faunal Zone B, early Miocene in age [28]–[33], [55]. Margo's Immense Might, Phil Found It, R12C and Souvenir Sites are of unknown age.

#### Revised species diagnosis

*Balbaroo fangaroo* differs from *B. nalima* sp. nov. (the only other species of *Balbaroo* for which the cranium is known) in having: a deeper rostrum with more inflated maxillae anteriorly; a less anteriorly extensive lacrimal; a smaller subsquamosal foramen; a more postorbitally constricted cranium; convergent frontal crests; more ventrally-extensive mastoid processes; larger C1; proportionately shorter DP3 with a proportionately smaller and shorter protocone, metaconule and stA; a less elongate P3/p3 whose longitudinal axes are deflected buccally with respect to the molar row; a concavo-convex (in lateral view) longitudinal crest on P3; a shorter diastema with a stronger diastemal crest; a more posteriorly extensive mandibular symphysis; a reduced mandibular foramen; a small i2 that abuts i1 posteriorly; three anterior cuspids on the occlusal ridge of p2; a weaker paracristid and crista obliqua on m1; a more linear, less arcuate hypolophid on m1; a narrower trigonid on m1; and a less distinctly increasing posterior molar gradient.

*Balbaroo fangaroo* differs from *Balbaroo gregoriensis* in the following features of m1: a more centrally positioned protoconid that is subequal in height to the metaconid.

*Balbaroo fangaroo* differs from *Balbaroo camfieldensis* in having a posterior mental foramen on the dentary.

#### Description

In addition to the original description of the skull, dentary and most elements of the dentition by Cooke [11], we provide the following amendments to the type description:

The i1 preserved in the Holotype was badly damaged [11]. Description here is based on a complete i1 preserved in juvenile specimen QM F20038 (Figs 6A–C). i1 is short, procumbent and triangular in lateral outline, with a smoothly convex buccal margin. In dorsal view, it curves lingually towards its distal end. Enamel covers the buccal face and dorsal, lingual half of the tooth. Prominent dorsal and ventral enamel flanges are visible from the lingual side of the tooth. A single, central longitudinal ridge runs the length of the lingual surface.

Cooke [11] noted the presence of a small alveolus for i2, directly posterior to i1. This tooth is preserved in QM F20038 (Figs 6A–C). In dorsal view (Figure 6C) the crown is ovoid in outline, tapering posteriorly with a flat dorsal surface. The tooth crown is bent anteriorly through greater than 90° such that it slopes anteroventrally while resting against the lingual surface of the dorsal enamel flange of i1.

The p2 of QM F20038 (Figs 6A–C) is a short, broad tooth with a central longitudinal occlusal ridge that is orientated slightly anterobuccally with respect to the molar row. The buccal and lingual faces of this ridge are steeply sloping. Three prominent anterior cuspids and a fourth, lower posterior cuspid, occupy the occlusal ridge with transcristids associated with the three anterior cuspids. The anterior-most cuspid is set back from the anterior-most point of the crown while the posterior cuspids overhang the posterior base. In buccal view, the longitudinal ridge is convex with its highest point occurring at the apex of the third cuspid. Anteriorly, a ridge of enamel descends from the anterior-most cuspid, extending about one-third of the way to the base of the crown. A ridge descends from the apex of the posterior-most cuspid and curves lingually as it runs to the base of the overhanging posterior part of the crown.

The dp3 of QM F20038 is triangular in occlusal outline (Figure 6C), tapering in an anterior direction. The protoconid is the tallest cusp on the tooth and is set centrally in the trigonid. A paracristid descends anterolingually from the protoconid to a large paraconid just posterior to the anterior tooth margin. The metaconid is positioned posterolingual to the protoconid. The metaconid, protoconid and paraconid are laterally compressed and form an extension of the shearing crest of p2. A postmetacristid descends almost to the floor of the interlophid valley. The hypoconid is shorter than the entoconid. The cristid obliqua extends slightly lingually as it descends into the interlophid valley where it meets a protostylid ridge that descends the posterobuccal flank of the protoconid. In posterior view the hypolophid forms a shallow ‘v’ with its lowest point buccally adjacent to the entoconid. A cristid descending from this point may represent a buccally displaced postentocristid. A preentocristid extends anteriorly from the entoconid apex into the interlophid valley.

Description of DP3 is based on QM F31594, a left partial maxilla with DP3-M2 and unerupted P3 (Figure 6D–E). DP3 is trapezoidal in occlusal view with its buccal margin longer than its lingual margin. There are five prominent cusps, the paracone, metacone, protocone, metaconule and stylar cusp A (StA or parastyle) and a much weaker swelling representing StE. The paracone and metacone are sub-equal in height and are the tallest cusps on DP3. The protocone and metaconule are subequal in height although the protocone is quite worn, yet the wear facet occurs slightly buccal of the apex. StA is well developed and taller than the protocone and metaconule. A protoloph does not connect the apices of the paracone and protocone, rather a weak transverse crest extends lingually into the longitudinal valley to meet a low preprotocrista. Wear is apparent on the protocone and anterior face of the metaconule. The well-developed metaloph connects the apices of the metacone and metaconule. The buccal faces of the paracone and metacone are steeply sloping. The lingual and posterior faces of the metaconule slope very gently to the base of the crown and are almost shelf-like. The protocone apex sits lingually opposite but slightly posterior to the paracone apex. The lingual metaconule apex is directly opposite the metacone. A sharp crest extends anterobuccally from the apex of StA and fades into the anterobuccal base of the crown at the anterobuccal corner of the tooth. A low, short premetaconule crista meets a low (albeit worn) postprotocrista. A short anterior cingulum extends from the anterolingual base of StA, fading into the crown at the tooth midline. The preparacrista is very low and short. The postparacrista is sharply defined and extends posteriorly and slightly buccally from the paracone apex into the transverse valley where it meets a low premetacrista. The postmetacrista descends posteriorly from the metacone apex, swelling slightly at the posterobuccal tooth corner to form StE. A weak posterior cingulum extends lingually from StE to the posterobuccal base of the metaconule. The transverse valley is shallow and open. The base of the protocone is broad anteroposteriorly whereas the base of the metaconule is broad bucco-lingually. The lingual margin is gently rounded whereas the buccal margin is relatively linear.

#### Remarks

In unworn juvenile specimens of *Balbaroo fangaroo* (notably QM F19578 and QM F20038 from RSO Site, QM F56984 from Camel Sputum Site, and QM F20063 and QM F19847 from Wayne's Wok Site) a remnant of a protostylid is evident as a short crest positioned directly posterobuccal to the protoconid that connects posterolingually to the cristid obliqua. This crest is not present in any of the adult individuals examined probably because it has been obscured by wear. However, in some less-worn adult specimens (e.g., QM F56981 from RSO Site), a distinct wear facet is found in the position of the short crest of juveniles, suggesting that this crest is worn flat by the time m4 erupts. Originally, the presence of this protostylid crest led Cooke [9] to assign the immature examples of *B. fangaroo* to *Wururoo*, the only known species of which, *W. dayamayi*, possesses a protostylid crest on m1 [6]. Re-evaluation of this material, in light of our new fossils, shows that these remains are actually indistinguishable from *B. fangaroo*. Consequently, we reassign the following specimens to this taxon: QM F20038, QM F19578, QM F20071, QM F20851, QM F19847, QM F20078, QM F 56981 (AR11300), QM F56982 (AR13370), QM F56984 (AR11771). Previous attributions include *Wururoo* (sensu Cooke [3]) or *Wururoo* sp. 2 [8], [9].

Balbaroo nalima sp. nov.

(Figures 7–15; urn:lsid:zoobank.org:act:A9EF21ED-0075-4BEA-9008-3643ED9FD57D)

**Figure 7:**
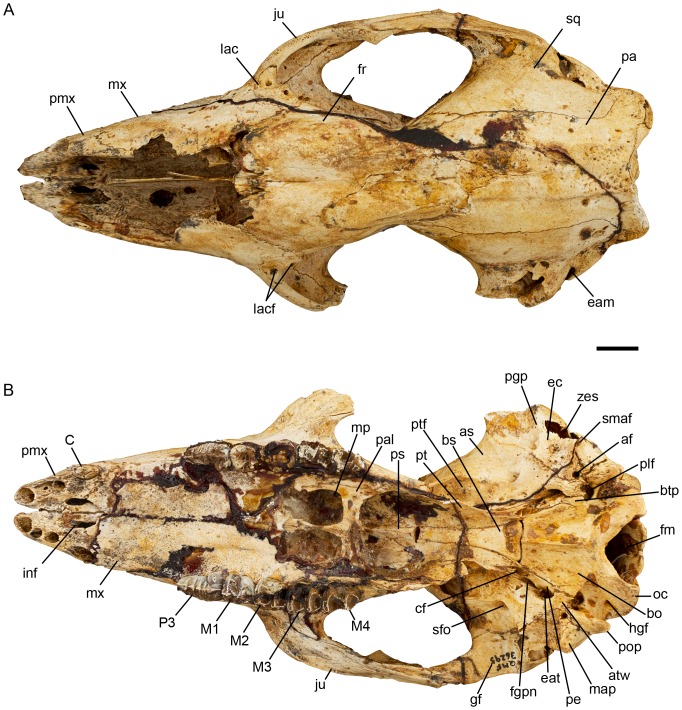
*Balbaroo nalima* sp. nov. (holotype QM F36295). (A) dorsal and (B) ventral views. Abbreviations: af, accessory foramen; as, alisphenoid; atw, alisphenoid tympanic wing; bo, basioccipital; bs, basisphenoid; btp, basioccipital tympanic process; C, canine; cf, caratoid foramen; eam, external auditory meatus; eat, exit for the auditory tube; ec, ectotympanic; fgpn, foramen for greater petrosal nerve; fm, foramen magnum; fr, frontal; gf, glenoid fossa; hgf, hypoglossal foramen; inf, incisive fenestra; ju, jugal; lac, lacrimal; lacf, lacrimal foramen; M1-M4, upper molar one to molar four; map, mastoid process; mp, maxillopalatine fenestra; mx, maxilla; oc, occipital condyle; P3, upper third premolar; pa, parietal; pal, palatine; pe, petrosal; pgp, postglenoid process; plf, posterior lacerate foramen; pmx, premaxilla; pop, paroccipital process; ps, presphenoid; pt, pterygoid; ptf, pterygoid fossa; sfo, secondary foramen ovale; smaf, stylomastoid foramen; sq, squamosal; zes, zygomatic epitympanic sinus. Scale bar equals 10 mm.

**Figure 8:**
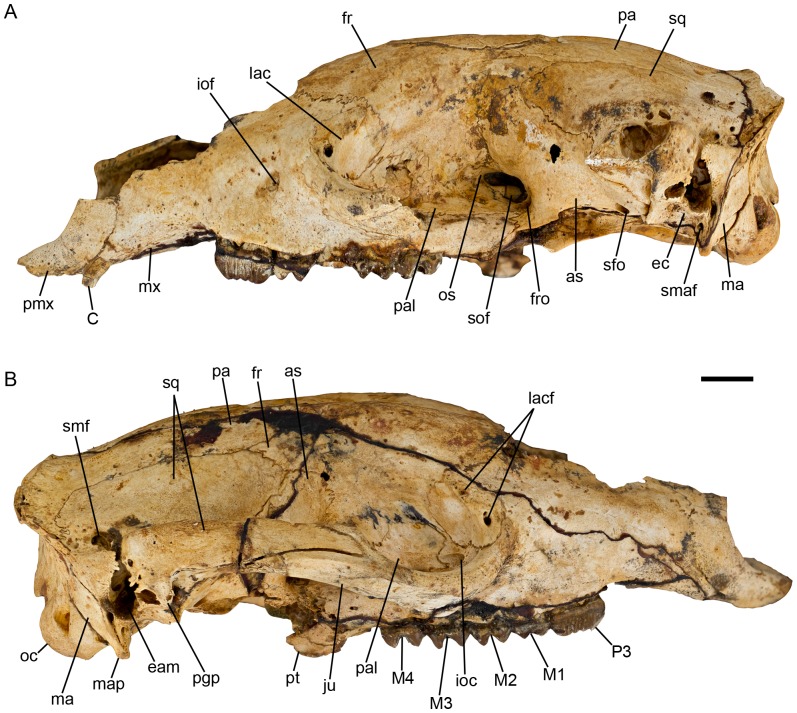
*Balbaroo nalima* sp. nov. Holotype QM F36295. (A) left lateral view and (B) right lateral view (B). Abbreviations: as, alisphenoid; C, upper canine; eam, external auditory meatus; ec, ectotympanic; fr, frontal; fro, foramen rotundum; ioc, infraorbital canal; iof, infraorbital foramen; ju, jugal; lac, lacrimal; lacf, lacrimal foramen; M1-M4, upper molar one to molar four; ma, mastoid; map, mastoid process; mx, maxilla; oc, occipital condyle; os, orbitosphenoid; P3, upper third premolar; pa, parietal; pal, palatine; pgp, postglenoid process; pmx, premaxilla; pt, pterygoid; sfo, secondary foramen ovale; smaf, stylomastoid foramen; smf, suprameatal foramen; sof, sphenorbital fissure; spf, sphenopalatine foramen; sq, squamosal. Scale bar equals 10 mm.

**Figure 9:**
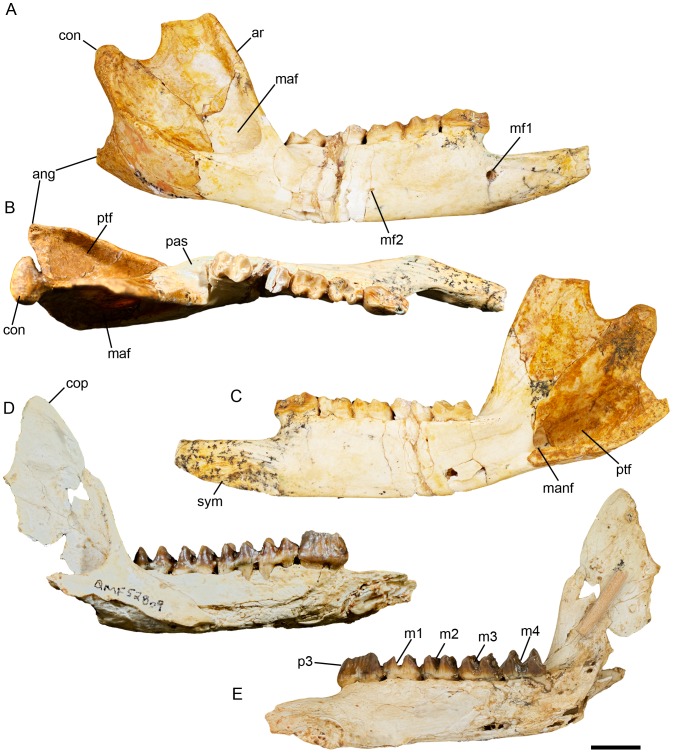
*Balbaroo nalima* sp. nov. right dentaries of paratypes QM F31446 (A–C), and QM F52809 (D–E). (A) and (D), lateral view; (B) occlusal view; (C) and (E), lingual view. Abbreviations: ang, angular process; ar, ascending ramus; con, mandibular condyle; cor, coronoid process; m1-m4, lower molar one to molar four; maf, masseteric fossa; manf, mandibular foramen; mf1, primary mental foramen; mf2, secondary mental foramen; p3, lower third premolar; pas, postalveolar shelf; pef, pterygoid fossa; sym, mandibular symphysis. Scale bar equals 10 mm.

**Figure 10:**
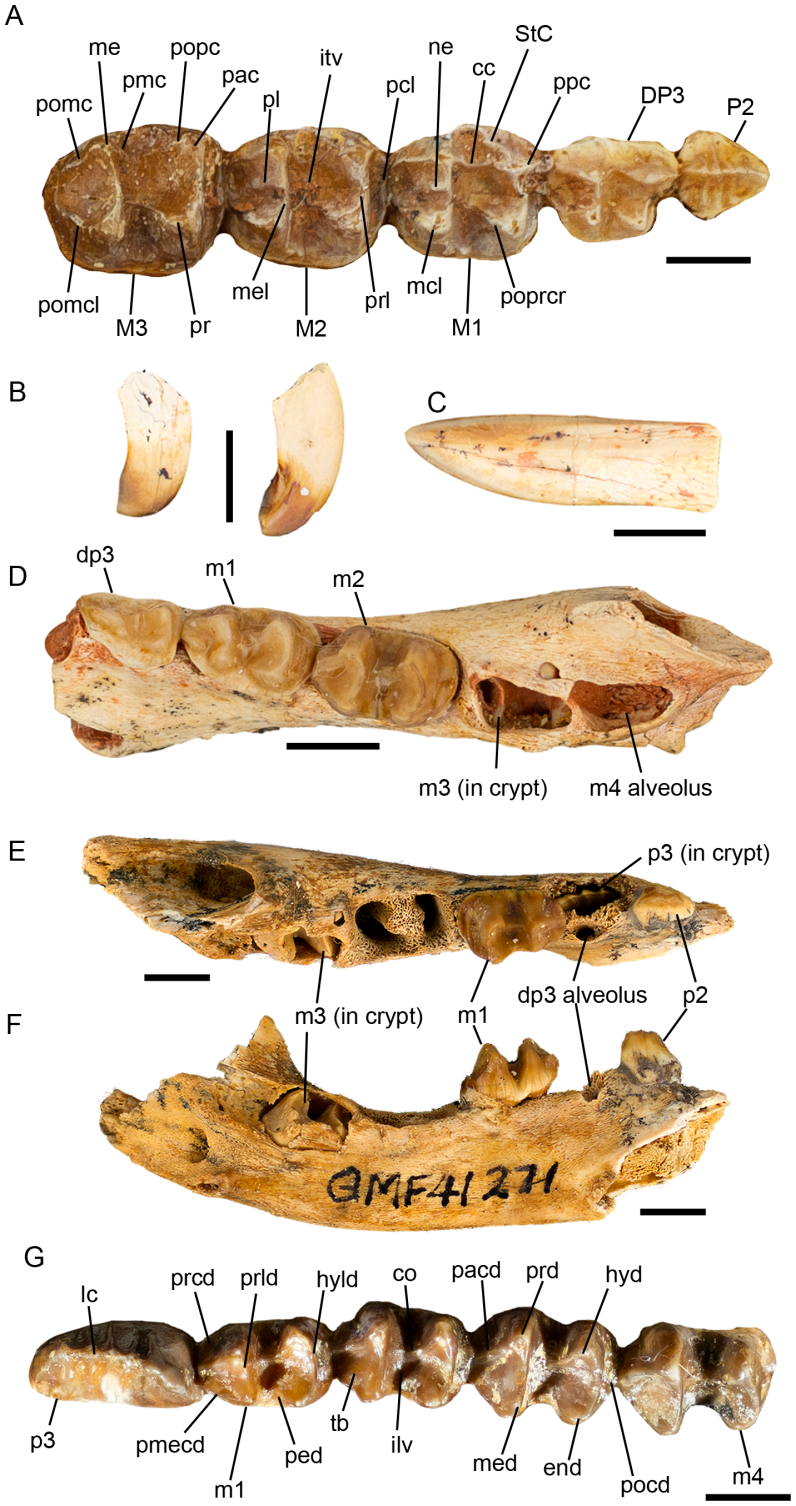
*Balbaroo nalima* sp. nov. dentitions. (A) juvenile upper (paratype QM F57025) dentition in occlusal view; (B–D) paratype QM F52811 juvenile, associated (B) left and right upper incisors, (C) right lower incisor, and (D) right dentary in occlusal view; (E–F) QM F41271, juvenile left dentary preserving p2 in (E) occlusal and (F) medial views; (G) QM F52809, adult right lower dentition. Abbreviations: cc, centrocrista; co, crista obliqua; P2, upper premolar two; DP3, deciduous upper premolar three; p2, lower premolar two; dp3, deciduous lower premolar three; end, entoconid; hyd, hypoconid; hyld, hypolophid; I1, upper first incisor; i1, lower first incisor; ilv, interlophid valley; itv, interloph valley; lc, longitudinal crest; M1-M4, upper molar one to molar four; m1-m4, lower molar one to molar four; mcl, metaconule; me, metacone; med, metaconid; mel, metaloph; ne, neometaconule; p3, lower third premolar; pac, paracone; pacd, paracristid; pcl, precingulum; ped, preentocristid; pl, postlink; pmc, premetacrista; pmecd, premetacristid; pocd, posteriorcingulid; pomc, postmetacrista; pomcl, postmetaconulecrista; popc, postparacrista; poprcr, postprotocrista; ppc, preparacrista; pr, protocone; prcd, precingulid; prd, protoconid; prl, protoloph; prld, protolophid; St C, stylar cusp C; tb, trigonid basin. Scale bars equal 5 mm.

**Figure 11:**
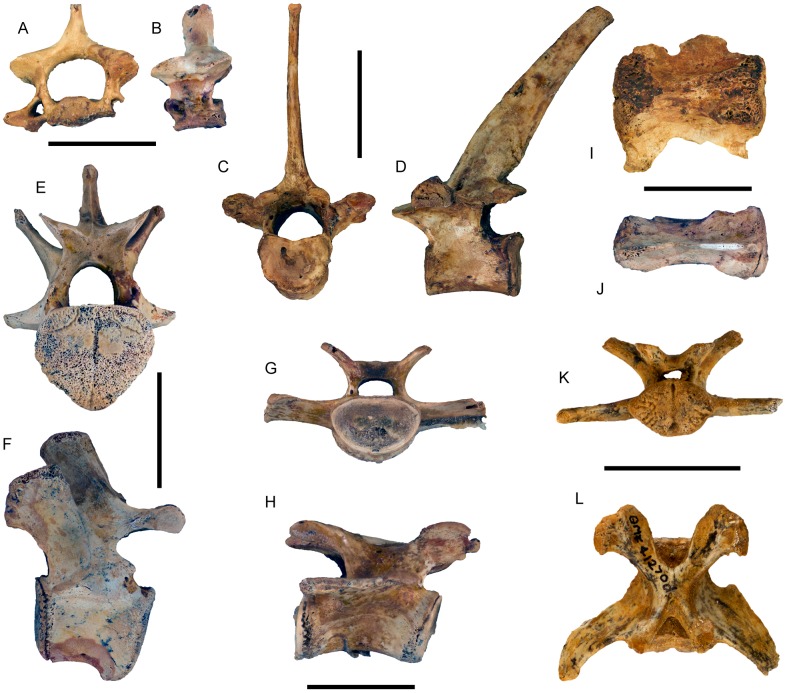
Vertebrae associated with dental remains of *Balbaroo nalima* sp. nov. All specimens pertain to QM F41234, except for (K–L) which derive from QM F41270. Cervical vertebrae in (A) posterior and (B) lateral views. Thoracic vertebrae in (C) posterior and (D) lateral views. Lumbar vertebrae in (E) posterior and (F) lateral views. Anterior caudal vertebrae in (G) anterior and lateral (H) views. Caudal vertebrae in ventral (I) and lateral (J) views. Anterior caudal vertebrae in posterior (K) and dorsal (L) views. Scale bar equals 10 mm in A, B; 20 mm in C–L.

**Figure 12:**
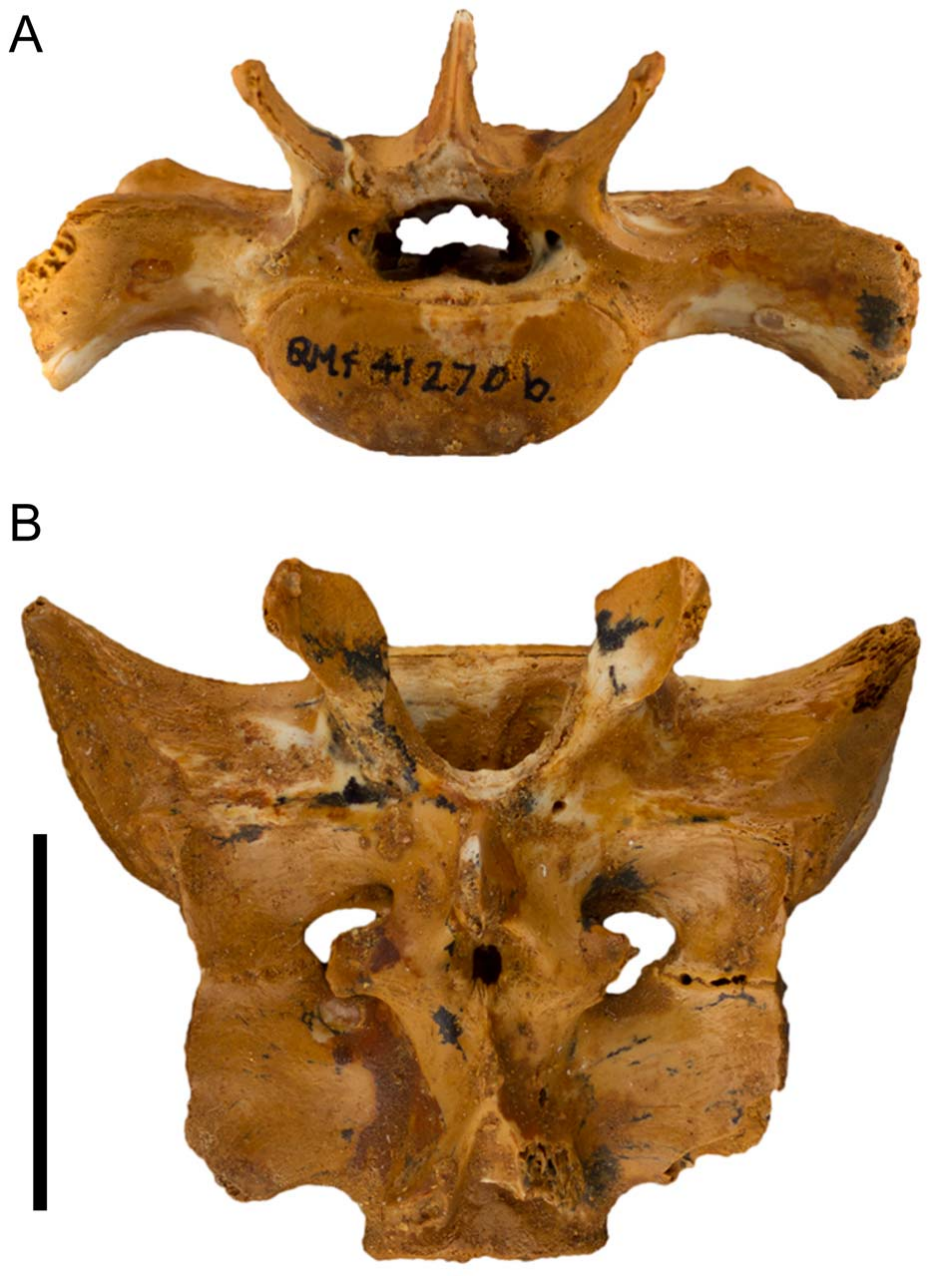
Sacrum (QM F41270) of *Balbaroo nalima* sp. nov. (A) anterior and (B) dorsal views. Scale bar equals 20 mm.

**Figure 13:**
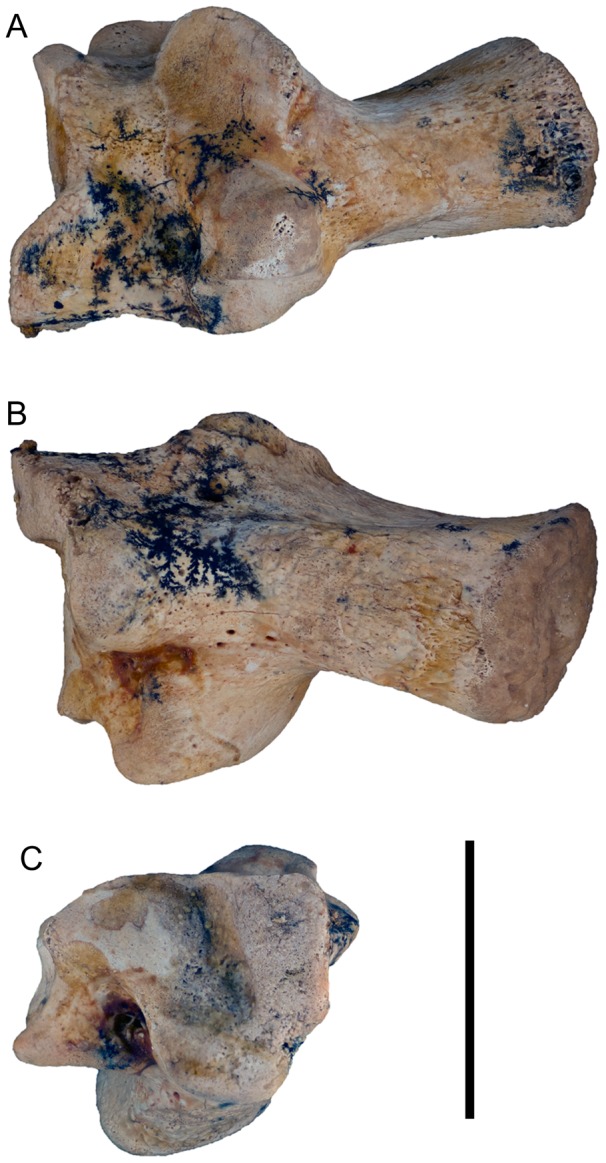
Calcaneum (QM F41234) of *Balbaroo nalima* sp. nov. (A) ventral, (B) dorsal, and (C) anterior views. Scale bar equals 20 mm.

**Figure 14:**
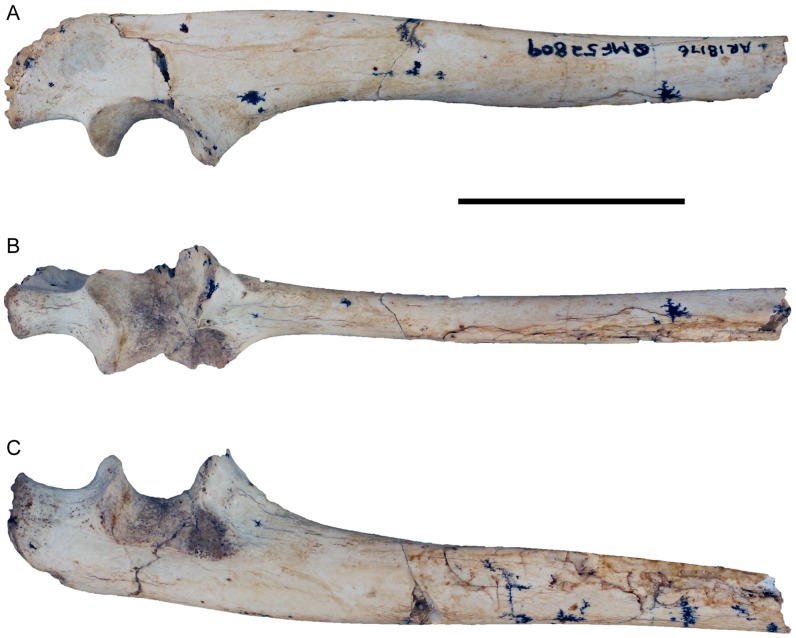
Right ulna (QMF52809) of *Balbaroo nalima* sp. nov. (A) medial, (B) anterior and (C) lateral views. Scale bar equals 30 mm.

**Figure 15:**
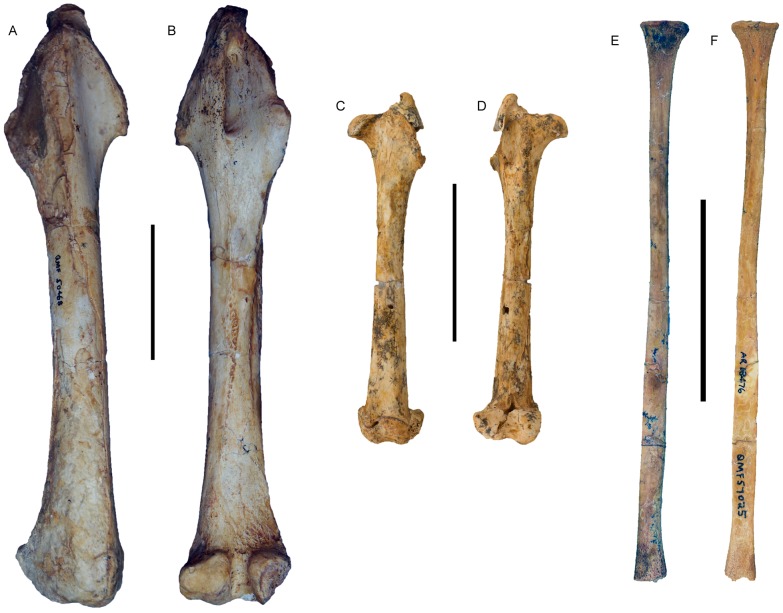
Femur (QMF50468) of *Balbaroo nalima* sp. nov. (A) anterior and (B) posterior views; (C–D) juvenile individual (QM F41270) shown at scale in (C) anterior and (D) posterior views. (E–F) Fibula (QM F57025) in (E) lateral and (F) medial views. Scale bars equal 50 mm.

#### Holotype

QM F36295, nearly complete adult skull with LC1, LP3, LM1-4 (broken), RP3, RM1-4. Nasals and dorsal surfaces of the premaxillae and maxillae are missing as is most of the left zygomatic arch.

#### Etymology

*nalima* is a Wanyii Aboriginal word meaning “lonely”, in reference to the rarity of fossil specimens of balbarids among Riversleigh's Faunal Zone C deposits.

#### Paratype

QM F 31446, right dentary with p3, m1-4; QM F41234, one cervical vertebra, one anterior thoracic vertebra, one lumbar vertebra, two caudal vertebrae, right ulna, left femur and left calcaneum; QM F41272, juvenile left partial maxilla with P3 (encrypted) dP3, M1; QM F50468, left femur; QM F57025, juvenile skull with left and right P2, DP3, M1-4, and associated right fibula; QM F52809, partial right dentary with p3-m4 and associated right ulna; QM F52811, juvenile right dentary with dp3, m1-2, m3 in crypt, with associated right i1, left and right I1, right M2 and left M3 QM F56964, partial left dentary with broken i1, and p3, m1-2.

#### Referred Material

From Alan's Ledge 1990 (AL90) Site: QM F41271, left juvenile dentary with p2, dp3 (isolated), m1, m3 in crypt, and isolated right m2; QM F41270, left femur, lumbar vertebra, sacrum, and four anterior caudals associated with QM F41271: QM F50397, partial juvenile skull with left and right encrypted M3, missing the rostrum, anterior section of right frontal, and much of the right alisphenoid and occipital region; QM F50415, associated right partial maxilla with P3-M4, and partial left dentary with root of i1, m1-4, missing ascending ramus and ventral border below m2-4; QM F50588, partial right dentary with p3-m3, missing anterior to p3, much of the ascending ramus, and the articular condyle; QM F56278, relatively complete juvenile skull with right M1, and encrypted left and right M3, missing the nasals, much of the basicranium, and the occipital region. From Cleft of Ages (COA) Site: QM F19889, left dentary with m1-2; QM F20007, right maxilla with broken P3, M1, broken M2; QM F20008, LM2; QM F20492, RP3; QM F20841, right maxilla with P3, M1-4; QM F20851, left dentary with p3, m1-2; QM F23194, right maxilla with M1-2; QM F24181, left maxilla with P3, M1; QM F24183, left dentary with m4; QM F24188, left dentary with broken m1, m2; QM F24220, right dentary with m3; QM F24430, RP3; QM F29706, left partial dentary with p3-m1; QM F29737, partial maxilla with RM1-2; QM F56282, Rp3; QM F31369, LP3. QM F36325, left partial maxilla with M1-3; QM F56965, left dentary with m3-4; QM F56281, partial left dentary with M2-3; QM F56283, Lp3. From Gag Site: QM F56284, juvenile left maxilla fragment with I1, P3 and M3 unerupted, M1-2 erupted; QM F57592, juvenile left dentary with i1, dp3, m1-2. From Fireside Favourites Site: QM F23205, right dentary with p3, m1-4; QM F31602, LM1. From Henk's Hollow Site: QM F20378, Rp3; QM F20417, Rp3; QM F20502, left dentary with p3, m1-2; QM F20518, right dentary with m1; QM F56986, right maxilla with broken P3-M2, M3; QM F56987, left maxilla with broken M1, M2-3. From Jim's Carousel 1 Site: QM F56280, partial left dentary with p3-m4, missing everything anterior to p3 and posterior to the postalveolar shelf. From Keith's Chocky Block Site: QM F52804, right dentary with m1-3; QM F52805, right dentary with m2; QM F56969, Lm3; QM F56970, Lm1; QM F56971, Lm3; QM F56972, Rm3; QM F56973, Lm3. From Rackham's Rock Pile Site: QM F20524, right maxilla with P3, M1-4. From Ringtail Site: QM 56279, partial right dentary with p3-m1, trigonid of m2, m3-4, missing everything anterior to p3 and the coronoid and angular processes.

#### Type Locality

AL90 Site, Southern Gag Plateau, Riversleigh World Heritage area, Lawn Hill National Park, northwestern Queensland, Australia.

#### Age and distribution

The AL90, Cleft of Ages, Fireside Favourites, Gag, Henk's Hollow Jim's Carousel, Keith's Chocky Block and Ringtail Sites are Depositional Phase C deposits [29]–[34]. They are interpreted to be middle Miocene in age on the basis of biocorrelation of their contained local faunas [32], [54], [56]–[58]. Little is known about the faunal assemblage from Rackham's Rock Pile Site to confidently assign it to a Faunal Zone. However, the presence of *B. nalima* may indicate a middle Miocene age for this deposit.

#### Species Diagnosis

*Balbaroo nalima* differs from all other species of *Balbaroo* in having: a shallower dentary with a less-robust horizontal ramus; a generally well-developed premetacristid; and a fully enclosed trigonid basin on m1 between the premetacristid, anterior cingulum and paracristid.

*Balbaroo nalima* differs from *Balbaroo fangaroo* (the only other species of *Balbaroo* for which the cranium is known) in having: a shallower rostrum anteriorly that has less inflated maxillae dorsally; a lacrimal that extends further onto the facial cranium; a larger subsquamosal foramen; a less postorbitally constricted cranium; parallel (as opposed to convergent) frontal crests; less ventrally-extensive mastoid processes; and reduced canines. However, the latter feature may reflect sexual dimorphism (i.e., comparing a potentially female *B. nalima* individual with a male *B. fangaroo* individual) rather than a diagnostic feature of the species. Other differences include: a proportionately larger DP3 with a proportionately larger and taller protocone and metaconule, broader lingual margin yet weaker StA; a more elongate P3/p3 whose longitudinal axes are more in line with the molar row; a more linear longitudinal crest on P3 that is horizontal in lateral view (as opposed to sinuous); a better developed posterolingual cusp on P3; a proportionately larger mental foramen; an i1 that projects less steeply from its alveolus; a longer diastema with a weaker diastemal crest; a less posteriorly extensive symphysis; a wider, more ovate mandibular foramen; two as opposed to three anterior cuspids on the occlusal ridge of p2; a stronger paracristid and crista obliqua on m1; a more arcuate hypolophid on m1; and a more distinctly increasing posterior molar gradient.

*Balbaroo nalima* differs from *Balbaroo camfieldensis* in having: a more posteriorly positioned ascending ramus originating distal to m4; a wider trigonid on m1; a more strongly-developed postentocristid that is continuous with the hypocingulid; and a narrower m4.

*Balbaroo nalima* differs from *Balbaroo gregoriensis* in the following features of m1: a more anteriorly orientated paracristid; a taller metaconid connected to the protoconid via the protolophid; and a posteriorly (rather than posterolingually) orientated postentocristid.

#### Description of the cranium

Description is based on the Holotype QM F36295 (Figs 2, 7–8), an adult skull with fully erupted P3-M4. The left P3 and right P3-M4 have intact crowns. The left C1 is missing tip of crown, left M1 missing posterolingual tooth corner, left M2 missing all of the crown, left M3-4 missing lingual face of the crown. Skull near complete missing only the nasals, the dorsal surfaces of the left and right maxillae and left premaxillae, and most of the left zygomatic arch. The unworn dentition and the distinct, unobliterated sutures (particularly those of the basicranium and occiput) suggest the individual was a relatively young adult.

Skull widest across the zygomatic arch at a point approximately 9 mm anterior to the most posterior extent of the jugal. Skull is deepest at a point vertically above the pterygoids (Figure 2, Figure 8A–B). The dorsal surface of the skull is convex in lateral view. The rostrum is downturned anteriorly with a distinct flexion evident (relative to horizontal plane of molar row) on the palatal surface opposite the mid-length of P3. The basicranium is elevated relative to the cheektooth row and the basioccipital is downturned posteriorly relative to the plane of the molar row. In dorsal view, the skull is markedly constricted posterior to the orbit in line with the vertical alisphenoid-frontal suture. As in other balbarids [11], [12], the frontals have inflated sinuses, thus exaggerating the postorbital constriction; however, this constriction is less pronounced than in *Balbaroo fangaroo* or *Nambaroo gillespieae*. A relatively deep median sulcus runs the length of the frontal-frontal suture becoming shallower and wider posteriorly. There are no postorbital processes of the frontals. Two weak frontal crests originate slightly anterior to the postorbital constriction and are continuous posteriorly with weak parietal crests that run parallel to, and 6 mm lateral to, the parietal-parietal suture. These crests slightly diverge laterally at their most posterior extent where they meet a well-developed nuchal crest. A sagittal crest is not developed. Posteriorly, the frontals form a wedge between the parietals. The anterior wings of the parietals terminate at the dorsal most point of the postorbital constriction (Figure 7A).

The rostrum is narrow, elongate (comprising 40% of skull length; taken from the tips of the premaxilla to the occipital condyles, 134.2 mm), and tapers anteriorly. The nasals are not preserved, but appear to have been confined to the dorsal surface of the rostrum (Figure 7A). Naso-frontal suture appears to have been anteriorly concave and positioned in line with the anterior margin of the orbit. A portion of the nasal septum and turbinales are preserved. The shape of the narial aperture is difficult to determine because of the broken premaxillae and nasals, however, its maximum width is 17.2 mm. In lateral view (Figure 8B), the narial aperture is retracted to the level of C1. The floor of the narial aperture is relatively shallow with a shallow trough separating the dorsal premaxillary surfaces of the incisor alveoli. The anterior palatal fenestrae (interincisive foramina) are evident at the posterior margin of this trough. The maxillae are mildly inflated anterodorsally, as they are both anteriorly and posteriorly on the palatal surface. In fact the palatal surfaces of the maxillae and premaxillae are comprised of relatively porous bone and the midline premaxillary suture within the incisor arcade is poorly fused and, as a consequence, a wide gap between the premaxillae separates the left and right I1 alveoli (Figure 7A–B). The lateral maxilla-premaxillary suture is convex becoming anteriorly concave ventrally where it crosses onto the palatal surface through the canine alveolus. The infraorbital foramen is elliptical (height 3.5 mm, width 2.2 mm) and positioned 10 mm dorsal to the anterior root of M1, and 10 mm anterior to the orbital fossa. The maxillae extend 2/3 the length of the rostrum. The maxilla-frontal suture is short (11.2 mm), highly interdigitated and obliquely orientated, extending posteroventrally from the junction of the nasal, frontal and maxilla. It defines the anterior border of the frontal sinuses and terminates at the anterodorsal border of the orbit at its junction with the lacrimal. From here the maxilla-lacrimal suture is arcuate following the curve of the orbit. It extends from the juncture of the maxilla, frontal and lacrimal bones to a point 2.3 mm anterior to the lacrimal foramen, where it meets the jugal.

Within the orbit, the maxilla-lacrimal suture is short and irregular and originates at the medial corner of the anterior zygomatic fossa. A short, thin, posterodorsal projection of the maxilla meets a similar anteroventral projection of the frontal on the medial wall of the orbit preventing contact between the lacrimal and palatine within the orbit. The medial wall of the orbit is mostly composed of the frontal dorsomedially, the palatines medially, the maxilla ventrally and the alisphenoid posteromedially. A posterodorsal wing of the frontal contacts the squamosal 8 mm posterior to the postorbital constriction, excluding contact between the alisphenoid and parietals. The frontal-alisphenoid suture is highly interdigitated but generally vertically orientated. It extends anteriorly from the alisphenoid-squamosal-frontal juncture, then curves ventrally and follows the path of the postorbital constriction on the medial wall of the orbit, curving posteriorly into the sphenorbital fissure. The frontal-squamosal suture is obliquely (anteroventrally) orientated and 8.3 mm long. It originates from its junction with the alisphenoid at a point 8 mm posterior to the postorbital constriction.

The structure of the maxillary suborbital shelf is similar to that of *Nambaroo gillespieae* in being narrow and tapering posteriorly [12]. The round (3.3 mm diameter) exit of the infraorbital canal is situated in the anterolateral corner of the suborbital shelf, entirely within the maxilla (Figure 8B). A short (8.1 mm) sulcus separates the infraorbital canal from the smaller, anteroposteriorly ovate (2.8 mm long) sphenopalatine foramen, which is situated entirely within the palatine. Several small nutrient foramina/canals perforate the suborbital shelf.

The ethmoid foramen is situated at the posteroventral corner of the frontal along the frontal-orbitosphenoid suture, and 5 mm anterior to the sphenorbital fissure. The palatine is extensive within the orbit, extending by a thin dorsal projection between the maxilla and the anteroventral wing of the alisphenoid, to then expand to occupy much of the mesial wall of the orbit. The palatine suture is highly interdigitated with the surrounding bones. It continues dorsally almost into the sphenorbital fissure, then curves anterodorsally bordering the orbitosphenoid, then extends roughly anteriorly from the level of the ethmoidal foramen, bordering the frontal and expanding anteriorly above the sphenopalatine foramen. A small foramen is positioned 5.4 mm posterior and slightly dorsal to the SPF.

The sphenorbital fissure (SOF) is large (∼9 mm long; Figure 8A). The foramen rotundum is situated posterolateral to the SOF and is separated from it by a thin bony wall. The lacrimal is roughly circular in shape and has a relatively extensive contribution to the facial region as it does also in *N. gillespieae* [12]. The lacrimal foramen is small (2.0×1.2 mm) and situated where the zygomatic arch projects from the facial region of the cranium. On the right side of the skull, the lacrimal foramen is divided by a short strut into a larger ventral and a smaller dorsal foramen. A small ridge-like lacrimal tuberosity is situated at the anterodorsal border of the orbit.

There is no distinct masseteric process, only a small rounded eminence of the ventral tip of the zygomatic process of the maxilla (Figure 8B). The maxilla-jugal suture follows the line of the anterior projection of the zygomatic arch. The zygomatic arch is composed almost equally of jugal and squamosal. The jugal process consists of a distinct medial ridge that borders dorsally a well-defined sulcus for the superficial masseter. On the lateral face of the zygomatic arch, the jugal process bifurcates into a short dorsal wing and a longer, deeper ventral wing, such that the squamosal process forms a wedge in the jugal on the lateral side. The dorsal wing of the jugal is deflected laterally along its dorsal border forming the postorbital process of the jugal. On the medial side of the zygomatic arch a ridge extends anteromedially from the postorbital process of the jugal and fades into the maxillo-jugal suture, delimiting the posterior border of the deep oval fossa within the anterior orbit. A larger ventral wing tapers posteriorly and flattens out ventrally to terminate at the glenoid fossa. The surface of the ventral wing of the jugal process is dominated by a relatively deep narrow fossa. Medially, within the orbit the jugal process of the zygomatic arch is dominated by a deep oval fossa that originates ventral to the lacrimal-jugal suture and excavates the anterolateral border of the zygomatic arch (also present in *N. gillespieae* and *B. fangaroo* [11], [12]). The sulcus fades posteriorly along the zygomatic arch just prior to the flange-like medial process of the jugal. The squamosal process of the ZA is more extensive laterally than medially. On the medial face of the ZA the squamosal is separated from the maxilla by a 15 mm wedge of the jugal.

The interincisive foramina are small (length ∼5.6 mm) and bordered by both the premaxillae and maxillae (Figure 7B). The posterior palatal fenestrae are moderate in size (maximum width 7.8 mm). Their anterior extent is difficult to determine due to breakage in that region but they would not have extended anteriorly beyond the transverse valley of M2. Posteriorly, they extend to the level of the protoloph of M4. They are separated medially by a very narrow strip of the palatines. A 3.6 mm strip of palatine separates the posterior palatal fenestrae from the interpterygoid fossa. The lateral section of the maxilla-palatine suture is preserved on the left side of the skull as a more or less linear suture that extends posteriorly through a small posterolateral palatine foramen preserved at the anterodorsal corner of the interpterygoid fossa.

The interpterygoid fossa is large and bordered by the pterygoids laterally and the palatines anteriorly. The pterygoids are incomplete with their most ventral projection not preserved. They extend anteriorly within the interpterygoid fossa beyond the level of the presphenoid-basisphenoid suture, although their sutural relationships become unclear at this point. A thin strip of palatine separates the pterygoids from the maxilla at the anterolateral corner of the interpterygoid fossa. A small triangular section of the presphenoid is evident on the roof of the interpterygoid fossa. The presphenoid-basisphenoid suture is widely open and narrow mediolaterally (3.1 mm).

The parieto-squamosal suture is linear for much of its length, running generally parallel to the midline parietal suture (Figure 7A). It becomes arcuate posteriorly curving laterally as it extends towards the nuchal crest where it terminates at a point anterodorsal to the dorsal-most extent of the mastoid. The midline parietal suture is completely fused (obscured) for the posterior 16 mm of its 26.7 mm length. This area is perforated by numerous small nutrient foramina. The parietals are widest (31.7 mm) at the nuchal crest. A large foramen is present on the left side of the cranium 10 mm anterior to the nuchal crest just lateral to the fronto-parietal crest.

The squamosal comprises the ventrolateral wall of the neurocranium. A large suprameatal foramen exists at the posterodorsal base of the zygomatic arch within a deep fossa. This fossa that houses several smaller foramina becomes shallower posteriorly in the direction of the nuchal crest. Anteroventrally, this fossa is continuous with the external auditory meatus (EAM). A small anterior projection of the squamosal almost meets the squamosal process of the zygomatic arch at its point of origin posteriorly, resulting in a narrow fissure between the suprameatal foramen and the base of the zygomatic arch, leading into the EAM. The EAM is bordered dorsally by the squamosal. Within the EAM, the squamosal forms a sinus that is inflated dorsal to the periotic and within the squamosal process of the zygomatic arch (zygomatic epitympanic sinus).

The basioccipital (Figure 7B) is trapezium-shaped with a prominent medial crest and moderately deep fossae on either side of this crest. The lateral tympanic process of the basioccipital meets a posterior wing of the alisphenoid and defines the posterior border of the exit for the auditory tube and the anterior border of the posterolacerate foramen. A small hypoglossal foramen is located at the anterodorsal border of the condyle. It connects to the foramen magnum internally and is overhung by the ventral border of the condyles. Lateral to the hypoglossal foramen is a smaller secondary hypoglossal foramen. The basisphenoid is a trapezium-shaped wedge between the pterygoids. It expands posteriorly to meet the basioccipital opposite the entrance to the entocarotid canal. The basisphenoid-basioccipital suture is linear and measures 9 mm mediolaterally. A thin posterior section of the presphenoid is visible ventrally, its anterior extent being overlain by the vomer.

The postglenoid process is moderately developed (Figure 8B), and the glenoid fossa is generally flat. Its medial border is defined by the squamoso-alisphenoid suture which runs obliquely (posteromedially) to its junction with the ectotympanic at the ventral process/tuberosity (see below).

The posterior lacerate foramen (PLF) is large and bounded by the basioccipital medially and posteriorly, the paroccipital process (POP) laterally, and the alisphenoid anteriorly (Figure 7B). The junction of the posterior extremity of the alisphenoid tympanic wing (ATW) and the basioccipital tympanic process underrun the PLF anteroventrally. As in *Nambaroo gillespieae* [12], at the anterolateral corner of the PLF is a rugose prominence marking the junction of the posterior ATW, the base of the mastoid process, the ectoympanic, and the base of the POP. The ATW is not inflated into a tympanic bulla. A small, round foramen (Figure 7B, af) occurs lateral to the rugose prominence within the ectotympanic at the medial base of the mastoid process. This foramen is also present in the juvenile cranium QM F50397 as a notch in the posterior margin of the ectotympanic. However, it was not found in any other macropodoid examined during this study. Lateral to this foramen there is a small stylomastoid foramen on the anterior face of the mastoid process at the mastoid/ectotympanic suture. Aplin [59; p.161] noted the presence in some potoroids of an accessory stylomastoid foramen (positioned lateral to the ‘true’ stylomastoid foramen) that served as an exit for the posterior auricular artery. It is possible that the small round foramen of *Balbaroo nalima* (Figue 7B; ‘af’) is actually the true stylomastoid foramen and what we regard to be the stylomastoid foramen (Figure 7B; ‘smaf’) is actually an accessory foramen. However, because the lateral most foramen's anatomical position corresponds with that of the stylomastoid foramen of other macropodoid taxa, we have taken the conservative approach and labelled it as such. Another slit-like foramen is evident in lateral view dorsal to and confluent with the stylomastoid foramen within the thin section of the squamosal wedged between the ectotympanic and the mastoid at the posteroventral base of the EAM.

The ectotympanic is obliquely aligned and roughly rectangular in shape. It floors the medial part of the EAM and is bordered anterolaterally by the squamosal, medially by the alisphenoid (oblique alisphenoid-ectotympanic suture) and posteriorly by the POP and mastoid. It forms the medial border of the postglenoid process and the floor of the postglenoid foramen. At its anteromedial-most point where it meets the alisphenoid and squamosal, it forms a distinct tuberosity. Within the postglenoid foramen a thin bony septum from the ectotympanic divides the foramen into large and small anteromedial foramina.

The petrosal has a thin anterior projection that forms the medial border of the canal leading into the anterior entocarotid foramen (and the lateral wall of the basioccipital). The anterior entocarotid foramen is roofed by the alisphenoid and floored by the pterygoids. Lateral to the anterior entocarotid foramen is a small slit-like foramen. Anterolateral to this is the secondary foramen ovale, which is smaller and narrower (less rounded) than that of *N. gillespieae* or *Balbaroo fangaroo* [11], [12]. A prominent ridge extends anteriorly and slightly laterally from the medial wall of the secondary foramen ovale and is continuous with the anteroventral wing of the alisphenoid, which extends more medially at this point to meet the palatine and maxilla ventral to the sphenorbital fissure. The rugose pterygoid fossa is an ovate area bounded by the pterygoids medially and laterally by the alisphenoid ridge laterally. Along the medial border of the pterygoid fossa lies the transverse foramen (within the alisphenoid), close to the alisphenoid-pterygoid suture.

The occiput (Figure 2D) is irregular in shape resulting from the prominent nuchal crest laterodorsally which overhangs the occiput on both the left and right sides of the skull in line with the condyles. The nuchal crest is not prominent at the dorsal apex of the occiput near the midline junction of the parietals. The squamosal does not contribute to the large nuchal crest, which is formed by the parietals and occipitals. The squamosal overlaps the occiput at its junction with the mastoid but forms only a small contribution to the occiput. The occipital condyles are relatively small and obliquely (dorsolaterally) orientated. The foramen magnum is relatively large and ovate with an irregular dorsal border comprised of a small supraoccipital notch bordered by left and right processes from the exoccipital, which overhang its dorsolateral margin. This notch is characteristic of many juvenile or incompletely fused adult marsupial crania [57]. The fossae for the rectus capitus major (RCMA) and rectus capitus minor muscles are relatively shallow. The left and right RCMA fossae are separated by a prominent vertical bulge near the nuchal crest. The paroccipital processes (POP) are weak and only slightly less ventrally extensive than the laterally flared mastoid processes. Neither the POP nor mastoid processes extend below the ventral border of the condyles.

#### Description of the dentary

Description is based on QM F31446 (Figure 9A–C), the most complete dentary available. QM F31446 is missing: the coronoid process of the ascending ramus; the tip of the angular process; a narrow section of the horizontal ramus through m3; and much of i1. The ascending ramus is fractured in numerous places. The dentary is elongate and narrow. The horizontal ramus is shallow, its greatest depth occurring more or less below the anterior root of p3. The ventral margin of the horizontal ramus is relatively straight and runs parallel to the occlusal tooth row. The mandibular symphysis extends posteriorly to below the anterior root of p3. It is highly rugose posteriorly. The mental foramen (1.8 mm high, 2.1 mm wide) is positioned 4.0 mm below the diastema just anterior to p3. A secondary posterior mental foramen is situated 9.7 mm ventral to the posterior root of m2. A weak diastemal crest is present anterior to p3 but it fades out midway along the diastema.

The ascending ramus rises at an angle of 120° relative to the cheek tooth row. The anterior and ventral borders of the masseteric fossa comprise flattened triangular surfaces as they do in *Balbaroo fangaroo* and *N ambaroo gillespieae* (for the attachment of the superficial layer of the masseter) [11], [12]. The masseteric fossa is deep and houses a large masseteric canal, which is confluent with the dental canal. The masseteric fossa has an arcuate inferior margin and a linear anterodorsal margin. The inferior margin of the masseteric fossa extends well below the level of the cheek tooth row. The masseteric eminence is weak. The condyle is small, subovate, broader laterally and tapers medially. It is positioned 17 mm dorsal to the occlusal surface of the molar row. The angular process of the dentary would have extended posteriorly beyond the level of the condyle.

The pterygoid fossa is deep and rugose and extends under the ascending ramus. The mandibular foramen is large and ovate and situated at the anterior margin of the pterygoid fossa, 12.1 mm posterior to m4. The mandibular foramen opens into the dentary canal. The anterior border of the ascending ramus on the medial side is flattened and grades into the post-alveolar shelf anteriorly. The post-alveolar process is weak.

#### Description of the dentition

Description of I1 (Figure 10B) is based on QM F52811; isolated left and right I1s found in association with a juvenile dentary. Enamel covers the entire crown. Very little wear is present. I1 is recurved, pointed and laterally compressed. In the holotype (QM F36295), a large, oval, anterior-facing I1 alveolus is preserved (Figure 7B) with two smaller (by approx. 50%) alveoli for I2-3 present posterior and slightly lateral to that of I1. The alveolus of I3 is only slightly larger than the alveolus of I2. Neither I2 nor I3 are preserved in any of the specimens examined.

A short diastema separates the alveoli of I3 and C1 (Figs 7B, 8A). C1 is preserved on the left side of the holotype (QM F36295). It appears slightly recurved but is damaged at the tip. On the right side of the skull, the C1 alveolus is as large as that of I1.

Description of P2 is based on paratype QM F57025 (Figure 10A). P2 is triangular in occlusal outline and as wide as it is long. Three cusps occupy a longitudinal crest which extends along the midline of the crown. Buccal and lingual transcristae extend from the apices of each cusp. The anterior and posterior cusps are equally as large, and larger than the central cusp.

Description of DP3 is based on paratypes QM F57025 (Figure 10A) and QM F41272. DP3 is trapezoidal in occlusal outline with the buccal margin longer than the lingual margin. The paracone is the tallest cusp on the tooth, followed by the metacone, protocone and metaconule. The buccal margins of the paracone and metacone are steeply inclined. The preparacrista runs anteriorly from the apex of the paracone and ends at the most anterior edge of the tooth. In QM F41272, a slight swelling on this crest may represent a weak stylar cusp A. Also on this specimen, a swelling on the anterior tooth margin at the anterolingual base of the paracone may represent a small protostyle or paraconule. The postparacrista runs posteriorly from the paracone and meets the premetacrista in the interloph valley at its buccal border. The protocone is directly lingual to the paracone. These cusps are not joined by a protoloph. Instead, the preprotocrista extends anterobuccally from the protocone apex and meets a short, weak transverse crest at the lingual base of the paracone. The postprotocrista runs from the protocone posterobuccally and ends at the anterobuccal base of the metaconule in the interloph valley. In QM F41272, the postprotocrista meets a short crest extending from the anterior face of the metaloph. The metaconule apex is positioned slightly more buccal to that of the protocone and lingually opposite the metacone to which it is joined by a low metaloph. Its base is anteroposteriorly compressed relative to the broad, square base of the protocone. The postmetacrista departs the metacone posteriorly and joins a small cusp (possibly StE) before curving lingually, ending at the posterobuccal flank of the metaconule. A postmetaconulecrista is absent in QM F57025, but weakly developed in QM F41272 where it is continuous with the posterior cingulum.

Description of P3 is based on the holotype (QM F36295, Figure 7B) unless otherwise stated. P3 is elongate and blade-like. It is dominated by a serrated longitudinal crest and a well-developed posterolingual cusp, the height of which is about half that of the total height of the crown. The buccal margin and anterior part of the lingual margin are convex in occlusal outline. The longitudinal crest lies on the midline of the tooth. It is relatively linear except for a slight curvature at its anterior and posterior ends that becomes more pronounced in worn specimens. Five cusps are present on the major crest with transcristae associated with each of them. The anterior- and posterior-most cusps are the largest. A short enamel ridge links the buccal side of the posterolingual cusp to the lingual flank of the posterior cusp on the major crest in some specimens (e.g., QM F24430). Cristae from the apices of the anterior and posterior cusps delineate the anterior and posterior margins of the crown. In lateral view, the anterior tooth margin is convex whereas the posterior margin is more linear and steeply inclined.

Description of M1 is based on the holotype (QM F36295, Figure 7B). M1 is bilophodont and roughly square in occlusal outline but with the anterior half of the tooth slightly wider than the posterior half. The protoloph and metaloph are sub-equal in length. The metacone is the tallest cusp on the crown followed by the paracone, metaconule and protocone. The preparacrista runs anteriorly from the apex of the paracone to a prominence at the anterobuccal tooth corner, regarded here as representing StA. The protoloph is slightly convex. The apex of the paracone and metacone are positioned slightly lingual to the buccal margin and the buccal faces of the paracone and metacone slope gently to the base of the crown. The protoloph is comprised of strong transverse crests that meet at the longitudinal midpoint of the tooth. In some specimens a weak forelink is present that extends anteriorly or anterobuccally towards StA and connects to the anterior cingulum forming a deep cleft between the preparacrista and the forelink. The anterior cingulum runs lingually from StA to the anterolingual base of the protocone. Two ridges are associated with the posterior of the paracone. The more buccal (stylar crest) extends posterobuccally from the paracone apex. A small swelling along its length represents StC (variable in size between specimens examined). The linear postparacrista runs posteriorly and joins to the premetacrista (centrocrista) in the interloph valley. StD is not developed but a small stylar shelf is present, buccal to the premetacrista. The prominent postprotocrista runs posterobuccally from the protocone and meets a short anterior crest from the metaloph in the transverse valley, just lingual to the metaloph midpoint. The metaloph is generally linear, curving slightly posteriorly at the metaconule apex. A neometaconule is present just buccal to the midpoint of the metaloph. A short postlink is associated with the neometaconule on the posterior flank of the metaloph. The linear postmetaconulecrista runs posterobuccally towards the base of the crown on the posterior face of the metaconule, then turns buccally and is continuous with a wide metacingulum which meets the postmetacrista at the base of the anterobuccal tooth corner. A short lingual cingulum is developed at the lingual end of the transverse valley, and is sometimes cuspate. In QM F36325, a kink in the postmetacrista represents a weak StE.

M2 is similar to M1 but differs in the following features: it is larger with all four major cusps taller; StA and StC are reduced to crests; the postparacrista is weaker and does not meet the premetacrista; the metaconular cuspule is absent in most specimens; the neometaconule is less distinct; a lingual cingulum is variably expressed; the protoloph can be longer and straighter (e.g., QM F36295, QM F50415) or more convex (e.g., QM F36325); and the forelink may be less pronounced (QM F36295) or more strongly developed (QM F36325, QM F50415).

M3 is similar to M2 but differs in the following features: the tooth is more posteriorly attenuated; the anterior cingulum is wider; a prominent crista runs lingually from the apex of the protocone to the crown base; and in some specimens the postparacrista and premetacrista are further reduced.

M4 (Figure 7B) is trapezoidal in occlusal outline with a rounded posterior margin. It differs from the more anterior molars in being more elongate and much narrower posteriorly because of a reduced metaloph. The protocone is taller resulting in a much more horizontal protoloph. Stylar cusp C and the stylar shelf are absent. The interloph valley is open both buccally and lingually, and the preparacrista and centrocrista are reduced.

Description of i1 is based on a juvenile isolated right i1 (QM F52811, Figure 10C). It is short, procumbent and triangular in lateral outline. The buccal margin is smoothly convex and covered by enamel, which forms prominent dorsal and ventral flanges that are visible in medial view. The medial surface is dominated by an extensive longitudinal ridge. Enamel also covers the dorso-medial half of the tooth. In dorsal view, the i1 curves medially towards its tip. In all specimens examined, the bone surrounding the i1 alveolus is broken or fragmented, preventing identification of alveoli that may have been there for an i2 or canine. Hence it is not known if these teeth, which are present in some relatively plesiomorphic macropodoids, were present in this taxon.

Description of p2 is based on QM F41271 (Figure 10E–F). The p2 is a short, ovoid tooth in occlusal view with a short central longitudinal occlusal ridge. The lingual face of the tooth is slightly steeper than the buccal face. Two anterior cuspids are present anterior to a tall third cuspid on the occlusal ridge with transcristids associated with the two anterior cuspids. Short anterior and posterior enamel ridges descend from the anterior-most and posterior-most cuspids, respectively, approximately one-third of the way to the base of the crown.

Description of dp3 is based on QM F52811 (Figure 10D). The dp3 is triangular in outline, with a relatively linear buccal margin and bulging posterolingual margin that tapers anteriorly. The centrally-positioned protoconid is the tallest cuspid in the trigonid. A paracristid descends anterolingually from the protoconid to a minute paraconid, the apex of which is positioned just posterolingual to the anterior margin. The metaconid is also minute and positioned posterolingual to the protoconid. The metaconid, protoconid and paraconid are laterally compressed. The hypoconid is much shorter than the entoconid. The cristid obliqua extends slightly lingually into the interloph valley where it meets a protostylid ridge off the posterobuccal flank of the protoconid. In posterior view, the hypolophid forms a shallow ‘V’ with its lowest point buccally adjacent to the entoconid. The postentocristid descends steeply from this point and forms a hypocingulid. A preentocristid extends anteriorly from the entoconid apex into the interloph valley.

Description of p3 is based on QM F52809 (Figs 9D–E, 10G). The p3 is large, approximately 50% longer than m1, with its long axis flexed slightly buccally out of alignment with the molar row. It has a semi-lunar occlusal outline with a relatively straight lingual and convex buccal margin and is wider posteriorly, tapering anteriorly. The occlusal margin is comprised of a longitudinal crest that lies along the approximate midline of the crown, but curves slightly lingually, both anteriorly and posteriorly, extending to the anterolingual and posterolingual base of the crown, respectively. The occlusal margin is horizontal in lateral view, and elevated with respect to the molar occlusal plane. The lingual surface of p3 is more steeply inclined than the buccal surface. There are four minor cuspids positioned between two larger cuspids at either end of the longitudinal crest. Lingual and buccal transcristids are associated with these six cuspids but posterior transcristids are not visible in more worn specimens (e.g., QM F31446).

Description of m1 is based on QM F23205, QM F52809 (Figs 9D–E, 10G) and QM F52811 (Figure 10D). This tooth is rectangular in occlusal outline, yet constricted at the level of the interlophid valley. The protolophid is narrower than the hypolophid, the protoconid being situated closer to the centre line than is the hypoconid. The protoconid is taller than the metaconid. In QM F23205, the trigonid basin is completely enclosed by the anteriorly orientated paracristid and premetacristid, which are linked across the anterior margin by the raised anterior border of the anterior cingulid. In relatively unworn specimens (e.g., QM F52811, F41271, F29706) and, the premetacristid is not continuous, and is weakly expressed anterior to the metaconid with a shallow valley separating it from a distinct cuspid at the anterolingual tooth corner which is continuous with the anterior cingulum buccally. This cuspid may be a lingually displaced paraconid, and is variably expressed (e.g., strongly crested in QM F41271; not crested posteriorly in QM F52811). A premetacristid is present in all adults examined and appears to form as a result of wear. Buccal to the paracristid the anterior margin is ventrally steeply inclined. The cristid obliqua crosses the interlophid valley to meet the posterior face of the protolophid lingual to the base of the protoconid. In the juvenile QM F52811, a faint protostylid ridge is present on the posterior flank of the protoconid, and in QM F41271, the protostylid is present as a short cuspid. The protostylid ridge is not present in any of the adult specimens examined. A strongly developed postentocristid is continuous with a hypocingulid, which crosses the posterior base of the hypolophid as far as the hypoconid. A short preentocristid is characteristic of wornspecimens but it often longer and more pronounced in relatively unworn specimens (e.g., QM F52811).

Description of m2 is based on QM F23205, QM F52809 (Figs 9D–E, 10G) and QM F52811 (Figure 10D). The m2 is roughly rectangular in occlusal outline. The linear protolophid and arcuate hypolophid are of about equal length. The premetacristid and paracristid are sharply defined. The broad anterior cingulid ends lingually at the anterior end of the premetacristid. A short but broad precingulid inclines ventrally at the anterior end of the paracristid. The cristid obliqua meets the posterior face of the protolophid closer to the centre line of the tooth than is the case on m1. Postentocristid, preentocristid and hypocingulid are as described for m1.

m3. This description is based on QM F23205 and QM F52809 (Figs 9D–E, 10G). This tooth is similar to m2 except that it is longer and wider in occlusal outline, and all cuspids are slightly taller.

Description of m4 is based on QM F23205 and QM F52809 (Figs 9D–E, 10G). This tooth resembles m3 in most respects but is more constricted in the interlophid area and has a hypolophid that is shorter than the protolophid.

#### Description of the postcranial elements

Postcranial elements (Figures 11, 12, 13, 14, and 15) referred to *Balbaroo nalima* include components of the axial skeleton, forelimbs, hindlimbs, and tarsi. These bones were found in immediate association with diagnostic craniodental remains and/or morphologically identical postcranials from the type locality. For these reasons, they are attributable with confidence to this taxon. A series of vertebrae, an ulna, femur, and calcaneum (QM F41234) were also found together, and are structurally compatible with QM F41270, QM F52809, and QM F50468, all of which have co-occurring craniodental remains.

The cervical vertebra (Figure 11) recovered with QM F41234 is incompletely preserved but clearly possessed an opisthocoelous centrum and subtriangular neural arch outline with a vertically orientated neural spine (Figure 11A–B). The succeeding anterior thoracic vertebra (Figure 11C–D) is similarly opisthocoelous and has a smoothly rounded ventral surface on the centrum. Its complete neural spine is caudally orientated, and the prezygapophyses are prominent relative to the reduced postzygapophyses. Parapophyses were evidently absent. Like the thoracic, the lumbar vertebrae recovered with QM F41234 (Figure 11E–F) and QM F41270 are both opisthocoelous, keeled ventrally, and have reduced transverse processes and anapophyses. The prezygapophyses are large and lobate, and the sulci for the ligamentum flavium are deeply excavated. The sacral complex of QM F41270 (Figure 12) comprises two centra that are fused but which retain a sutural trace traversing both the centra and transverse processes where it encloses the sacral foramen. The posterior centrum articular face lacks an epiphysis. However, the anterior epiphysis is still intact and reveals a flat articular surface. The sacroiliac connection is largely restricted to the first sacral vertebra, and is deflected anterolaterally for its contact with the ilium. The prezygapophyses on the first sacral are transversely narrow and prong-like. The postzygapophyses of the second sacral are broken off. The caudal vertebrae of QM F41234 (Figure 11G–J) and QM F41270 (Figure 11K–L) are distinguished by their dorsoventrally compressed centra. Original positioning of the caudals within the anterior part of the series is evidenced by their prominent pre/postzygapophyses or transverse processes, which extend along most of the lateral surfaces of the centra.

The left calcaneum found with QM F41234 (Figure 13) is missing its epiphysis, indicating osteological immaturity. Nevertheless, the tuber calcis is clearly dorsoventrally deep and weakly flared posteriorly. The plantar rugosity is anteroposteriorly short with a shallow plantar sulcus. The sustentaculum tali is noticably broad and triangular in outline. The astragalar facet is not medially constricted. The facet for the lateral fibular malleolus is weakly developed and the deeply stepped cuboid articulation comprises an obliquely projecting dorsolateral facet separated from the median-ventral facet by a shallow groove. The inset dorsomedial facet is continuous with the median-ventral facet.

The partial ulnae preserved with QMF52809 and QM F41234, exhibit straight ventral shaft profiles (Figure 14). The olecranon process is dorsoventrally tall but lacks its terminal epiphysis. The semilunar notch is bounded by the coronoid and anconeal processes; the latter bearing a prominent midline projection. The trochlear facet is shallowly cupped in medial view with a sub-vertical anterior border. The capitular facet is laterally inclined for accommodation of the distal humeral condyle.

The left femur (Figure 15A–B) associated with QM F50468 is missing its articular head, but retains its greater trochanter adjoining the centrally positioned trochanteric fossa. The lesser trochanter bears a prominent pectineal process. The femoral shaft manifests a well-defined ridge marking the insertion point for the m. quadratus femoris and adductor muscle complex. This is likewise evident in the juvenile specimen QM F41270 (Figure 15C–D), which also retains a hemispherical articular head offset and perpendicular to the long axis of the femoral shaft.

The near complete right fibula occurring with the subadult skull, QM F57025, lacks both of its epiphyses (Figure 15E–F). The shaft is D-shaped in cross-section. The tibia contact extends along approximately half of the shaft length.

## Results

### Metric analysis

Coefficients of variation (CVs) for *Balbaroo nalima* dental variables (Table S3) range between 3.14 (m2L) and 11.59 (M4PW) generally falling within the expected range (4–10) for a single mixed-sex population [35]. The second lower molar was the least variable tooth in overall size (CVs 3.14–4.54) while M4 was the most highly variable tooth (CVs 7.14–11.59), however sample size (n = 5) was limited for the latter. Intraspecific metric dental variation was generally lower for *Balbaroo fangaroo* with coefficients of variation (Table S4) ranging between 4.22 (m1L) and 7.12 (P3W).

Bivariate plots (Figs 3–4) show a strong clustering of *B. nalima* specimens for most dental variables. Species of *Balbaroo* are strongly separated on the basis of premolar and posterior molar (m3, m4, and M4) dimensions, with the latter reflecting the more greatly increasing posterior molar gradient of *B. nalima* compared with *B. fangaroo*. Considerable overlap is evident between species of *B. nalima* and *B. fangaroo* in both their M1 and m1 dimensions; however, these species are clearly separated by discrete character state differences in their teeth (see Species Diagnosis). *Nambaroo gillespieae* is obviously distinguished from *Balbaroo* spp. by being much smaller, and by a reduced P3 and p3. *Balbaroo camfieldensis* and *Nambaroo bullockensis* are not discriminated, but deviate from *B. nalima* in their m3, m4 and upper molar dimensions.

### Phylogenetic analysis

**Figure 16:**
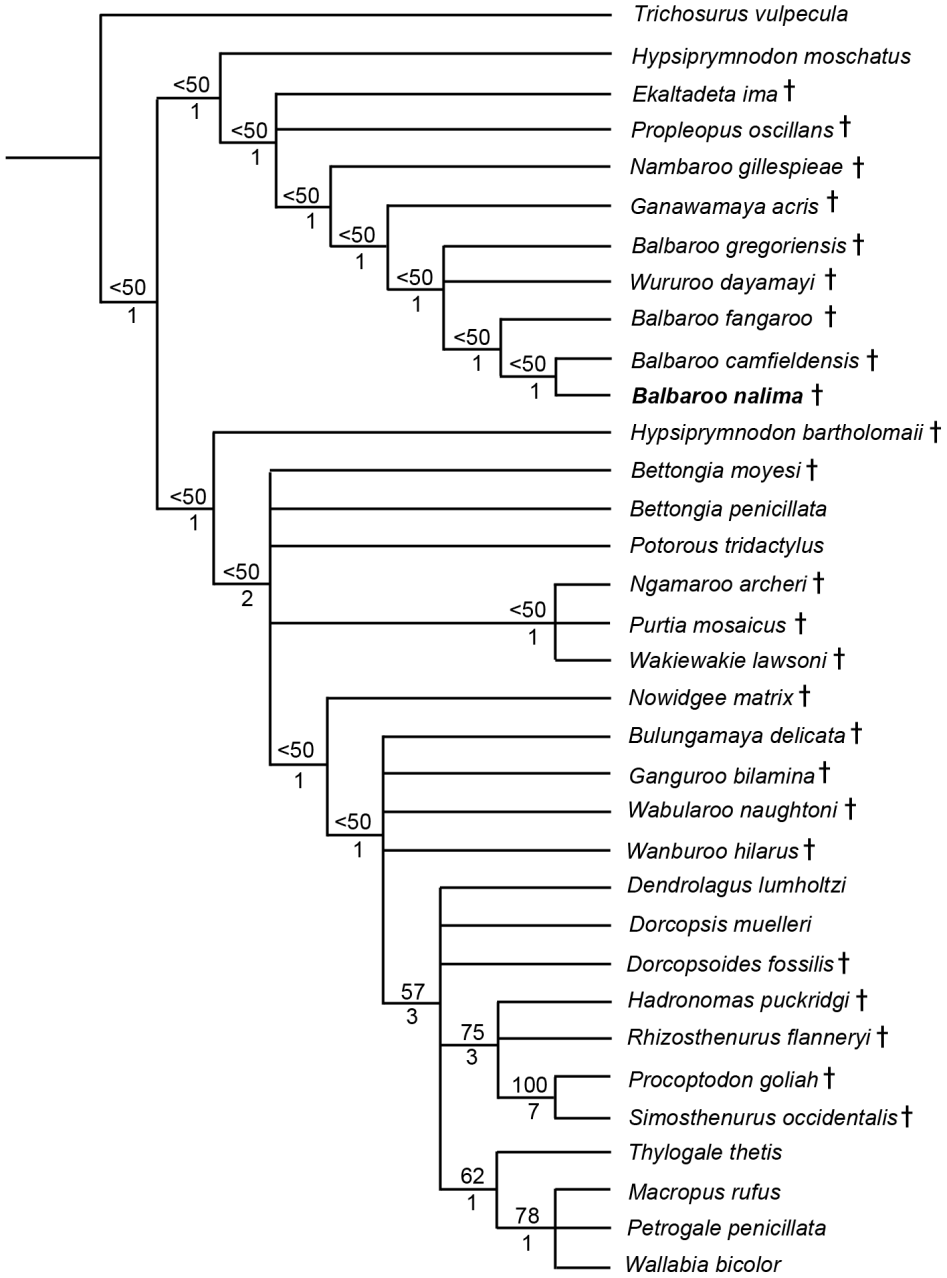
Ordered phylogenetic analysis based on our modified version of the Kear and Pledge [13] phylogenetic data set. Strict consensus of 96 most parsimonious trees (tree length  = 288; consistency index excluding uninformative characters  = 0.4375; rescaled consistency index  = 0.3168). Numbers above branches indicate bootstrap values (1000 replicates) and numbers below branches indicate decay indices. Fossil taxa are indicated by †. *Balbaroo nalima* sp. nov. is highlighted in bold.

**Figure 17:**
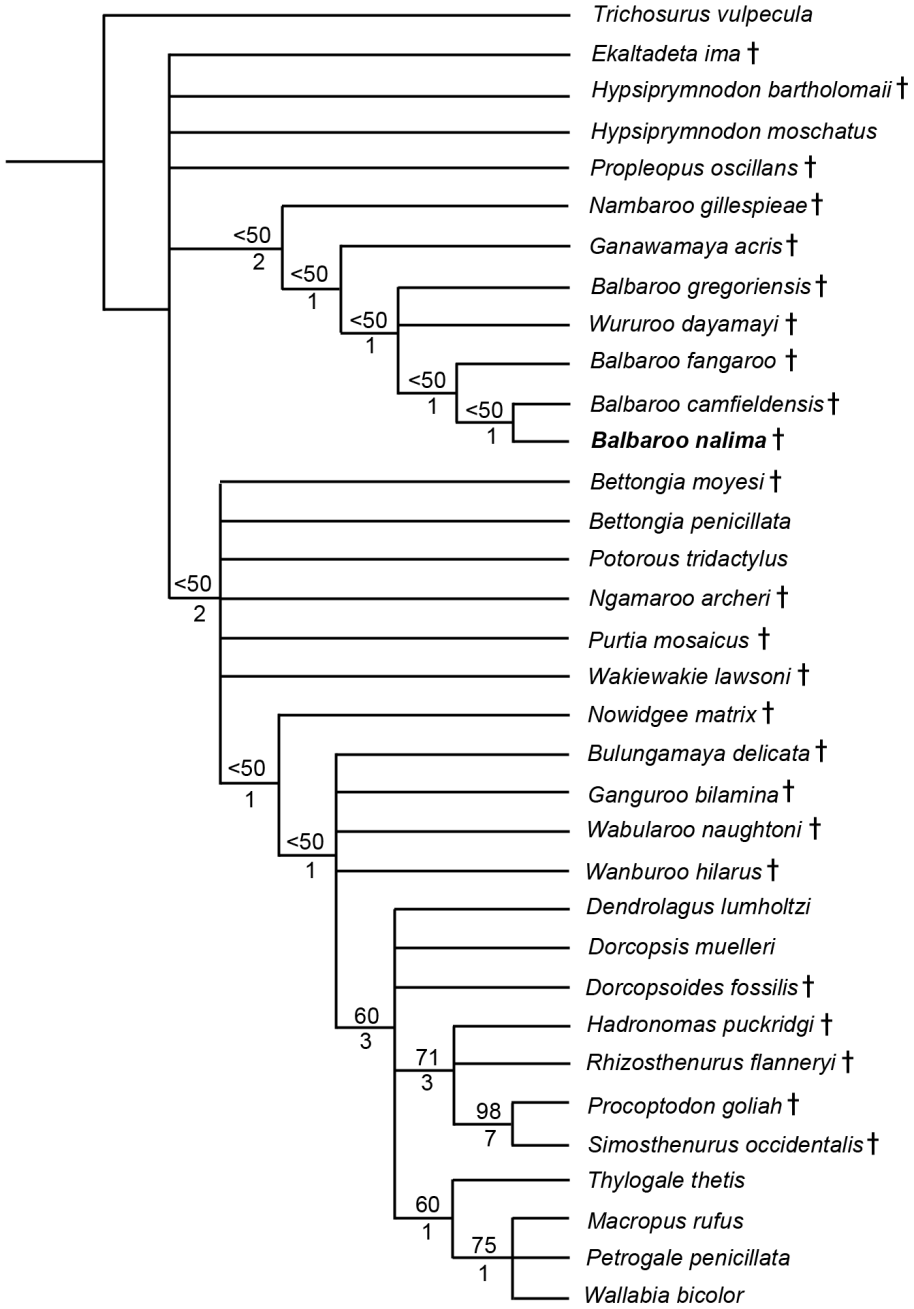
Unordered phylogenetic analysis based on our modified version of the Kear and Pledge [13] phylogenetic data set. Strict consensus of 140 most parsimonious trees (tree length  = 284; consistency index excluding uninformative characters  = 0.4437; rescaled consistency index  = 0.3207). Numbers above branches indicate bootstrap values (1000 replicates) and numbers below branches indicate decay indices. Fossil taxa are indicated by †. *Balbaroo nalima* sp. nov. is highlighted in bold.

**Figure 18:**
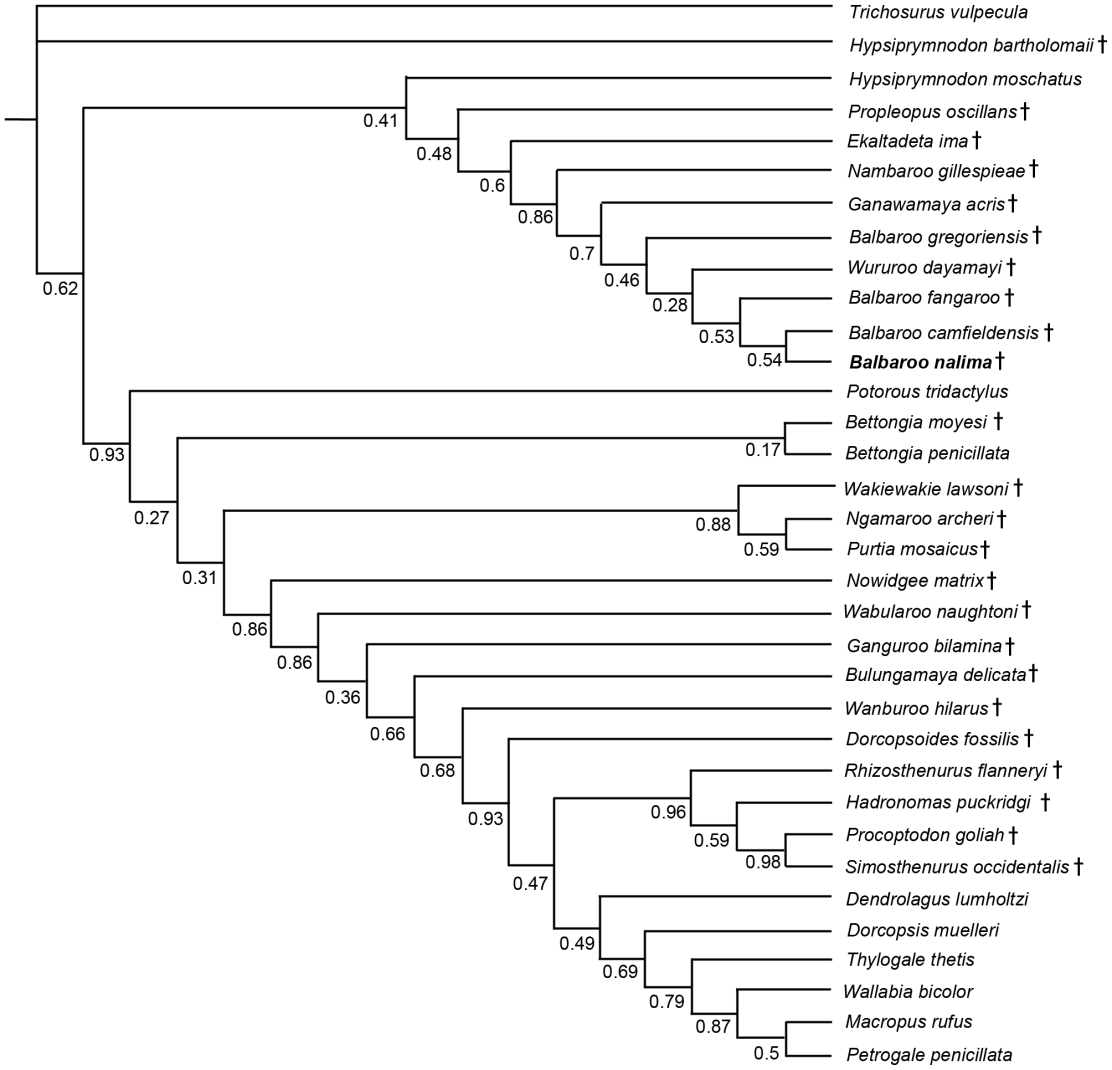
Ordered Bayesian analysis of our modified version of the Kear and Pledge [13] phylogenetic data set. Numbers at nodes represent Posterior Probabilities (PP). Fossil taxa are indicated by †. *Balbaroo nalima* sp. nov. is highlighted in bold.

**Figure 19:**
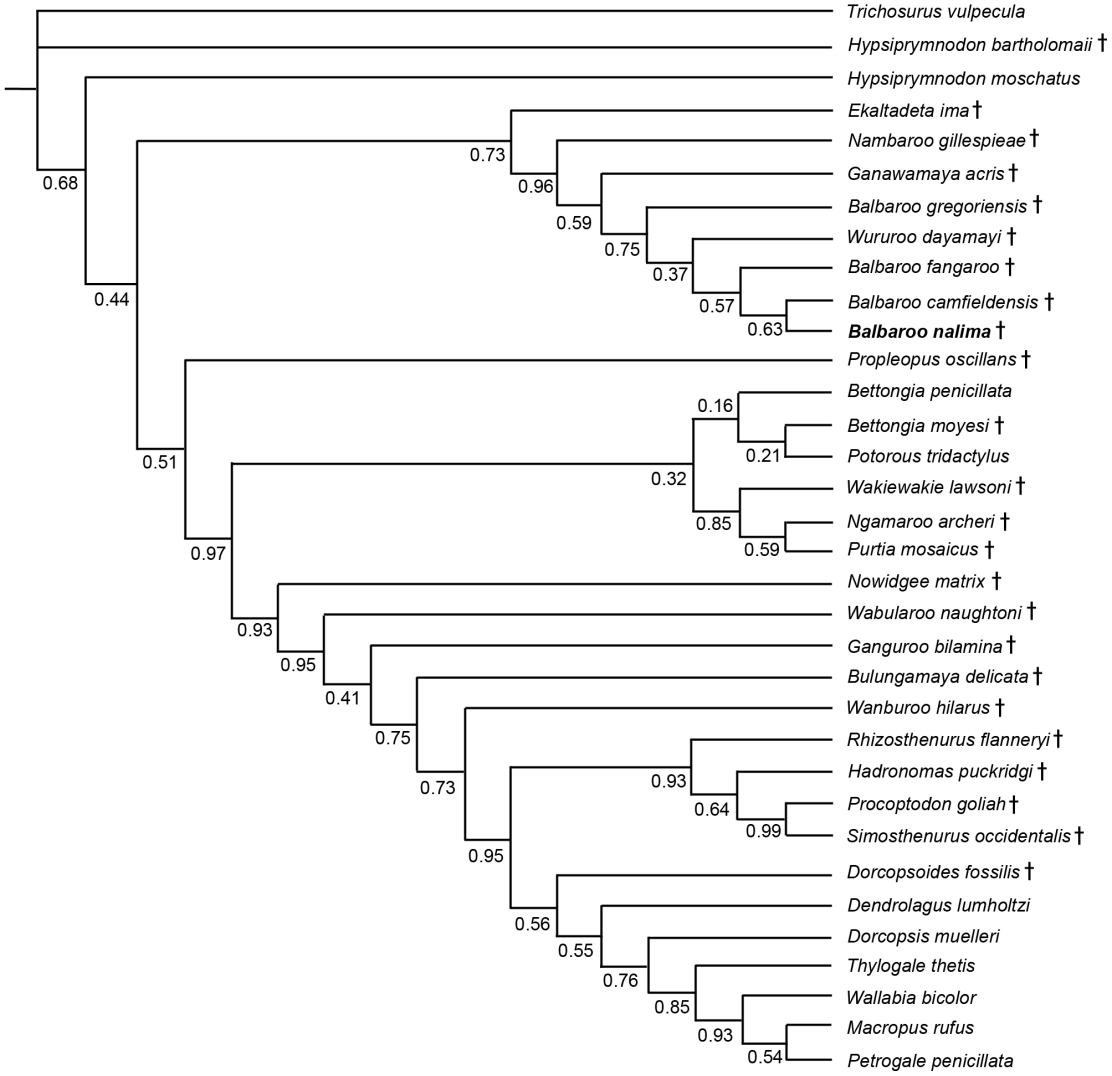
Unordered Bayesian analysis of our modified version of the Kear and Pledge [13] phylogenetic data set. Numbers at nodes represent Posterior Probabilities (PP). Fossil taxa are indicated by †. *Balbaroo nalima* sp. nov. is highlighted in bold.

Parsimony analyses using “*Trichosurus vulpecula*” as the user-specified outgroup, with uninformative characters excluded, and state ordering enforced, yielded 96 MPTs of length {L}  = 288 (Consistency Index {CI}  = 0.4375, Rescaled Consistency Index {RCI}  = 0.3168: see strict consensus in Figure 16), and returned *Hypsiprymnodon moschatus* + *Propleopus oscillans* + *Ekaltadeta ima* + Balbaridae as a monophyletic sister clade to all other Macropodoidea (*sensu* Meredith et al. [14]). Unfortunately, support for this grouping was weak (bootstrap/Bremer <50/1), as previously acknowledged by Kear et al. [12] and Kear and Pledge [13]. Indeed, bootstrap/Bremer support values (<50/1) were negligible for almost all higher-level clades (Figure 16) except *Dorcopsoides fossilis +* Macropodidae *sensu stricto* (57/3), and Sthenurinae (75/3). Irrespectively, balbarid taxa maintained coherence following imposition of unordered states (140 MPTs of L = 284, CI = 0.4437, RCI = 0.3207: Figure 17), as well as within our Bayesian topologies (PP = 0.91 averaged from ordered (Figure 18) and unordered (Figure 19) runs), which unanimously excluded Balbaridae from Potoroidae + Macropodidae *sensu lato* (PP = 0.95) thus advocating placement along the macropodiform stem (*sensu* Cooke and Kear [2], Kear and Cooke [7]). Inspection of the individual parsimony character transformations found that the balbarid constituent taxa *Nambaroo gillespieae* + *Ganawamaya acris* + *Wururoo dayamayi* + *Balbaroo* spp. were united by homoplastic (ch.24.2, ch.26.1) or symplesiomorphic (ch.32.0) dental states. Moreover, potential postcranial apomorphies (ch.104.1, *sensu* Kear et al. [12]) could only be scored for *Balbaroo nalima* and *N. gillespieae*, thus their significance is unclear without data from other fossils. The genus *Balbaroo* incorporated *W. dayamayi* to form a basal polytomy with *Balbaroo gregoriensis* (Figures 16–17), a result consistent with our proposed synonymy. These taxa specifically shared a narrow trigonid basin on the m1 (ch.30.1; secondarily expanded in *B. nalima,* ch.30.2), but otherwise manifested traits widely expressed in other clades (ch.43.1). Placement of additional species within *Balbaroo* likewise relied upon convergent dental features (ch.29.1 to unite *Balbaroo fangaroo + B. nalima* + *Balbaroo camfieldensis*; ch.28.1 for *B. nalima* + *B. camfieldensis*), which prompts our conclusion that existing character sets reliant upon fragmentary dental remains and limited postcranial material are inadequate for robustly delimiting balbarid interrelationships.

Inclusion of *P. oscillans* and *Hypsiprymnodon bartholomaii* within our analyses failed to precipitate a monophyletic Hypsiprymnodontidae (see [12]). Further exploration via a posteriori pruning following the semi-strict Adams consensus topology [60]–[62] demonstrated that all hypsiprymnodontids, with the exception of *E. ima*, functioned as “wildcards” (*sensu* Nixon and Wheeler [63]), and thus were affected by homoplasy and/or missing data. Placement of *H. moschatus* + *P. oscillans* + *E. ima* with Balbaridae relied upon symplesiomorphies (ch.31.0 ordered, ch.43.0) or derived states that were widely distributed amongst macropodiforms (ch.34.1, ch.89.1 ordered, ch.105.1 ordered). Despite this, some traits were specifically shared with *Balbaroo* spp. and *N*. *gillespieae* (ch.7.1). Nesting of *E. ima* + Balbaridae was derived on the buccal and dorsolingual extent of its i1 enamel (ch.35.1). *Hypsiprymnodon* spp., on the other hand, was consistently polyphyletic: *H. bartholomaii* forming the sister to Macropodoidea based on P3/p3 length (ch23.1) but with identical scores recorded in *H. moschatus* (i.e., “1”). *Hypsyprymnodon moschatus* and *H. bartholomaii* were in fact indistinguishable in their coding except for the primitive retention of an alisphenoid-parietal contact in *H. bartholomaii* (ch.7.1), which was alternately treated as a reversal or symplesiomorphy by our phylogenies.

## Discussion

### Palaeoecology

*Balbaroo nalima* sp. nov. displays the distinctive features of both the skull and postcranium that otherwise characterise *Balbaroo fangaroo* and *Nambaroo gillespieae* and support the hypothesis that balbarids occupied a unique niche – one no longer utilized by macropodoids within late Miocene to modern ecosystems. Shared structures of the cranium include: strongly developed nuchal crests; minimal flexion of the rostrum and basicranium relative to the plane of the tooth row; inflated frontal sinuses; hypertrophied mastoid processes; and the absence of an inflated auditory bulla. The last three of these traits were previously advocated as synapomorphies for Balbaridae [7], although tellingly, none was recovered by our phylogenies.

Cooke [11] noted that flexion of the basicranium in macropodoids was a derived condition associated with the development of an upright posture and bipedal saltation. In contrast, he suggested the absence of basicranial flexion in *B. fangaroo* combined with the presence of strongly developed nuchal crests and mastoid processes in the occiput (both indicative of powerful neck musculature) were adaptations to quadrupedal locomotion. At that time, the postcranial evidence to support this interpretation was lacking. Subsequent description of the partial skeleton of *N. gillespieae* [12], together with the *B. nalima* elements described here, imply that a quadrupedal gait, either in the form of bounding or slow pentapedal progression, might have been ubiquitous amongst balbarids.

Functionally, the postcranial osteology of *Balbaroo nalima* is therefore most closely compatible with *Nambaroo gillespieae* [12] among macropodiforms. In particular, the presence of compressed caudal centra constitutes a potential synapomorphy for these taxa, and is thought to reflect increased capacity for dorsoventral flexion, perhaps related to prehensility or slow “pentapedal” progression involving the tail [12]. Also conspicuous is the insertion structure for the m. quadratus femoris and adductor musculature on the posterior midline of the femoral shaft. Kear et al. [12] proposed that development of an incipient rugosity on the femur of *N. gillespieae* resembled those of primitive living macropodiforms, which consistently employ quadrupedal progression instead of bipedal saltation (e.g., *Hypsiprymnodon moschatus*: [64]). Certainly, the condition in advanced macropodids is noticeably different, with the muscle contact surface forming a heavily scarred “boss” (see [27]: p. 59–60; p. 69, fig. 32G–I), presumably associated with increased tensional stress at the site of attachment, and concomitantly, hopping gaits. Interestingly, the femur of *B. nalima* displays an intermediate morphotype, in which the m. quadratus femoris/adductor complex insertion manifests as a raised ridge (Figure 15). Whether this reflects greater propensity for saltation in comparison to *N. gillespieae* is unknown, but both taxa otherwise exhibit classical macropodiform traits (e.g., a “stepped” calcaneum-cuboid articulation) usually associated with specialization towards hopping locomotion [26].

Perhaps the most remarkable feature of *B. nalima*, *B. fangaroo* and *N. gillespieae* is the shared presence of enlarged canines in what are presumably browsing species characterised by lophodont dentitions. Medium to large canines are common in basal, omnivorous macropodiforms (e.g., species of *Hypsiprymnodon*, *Potorous* and *Bettongia*) and are likely plesiomorphic for the suborder. However, the “fang-like”canine teeth of *B. fangaroo* (as preserved in the paratype QM F30456) are hypertrophied to an extent not seen amongst any other macropodiform clade, suggesting they were not merely a plesiomorphic retention but were clearly functionally derived. Cooke [65] noted similarities in the canines of QM F30456 to those of several extant artiodactyl groups including the tragulids (Tragulidae) or Mouse deer of India and Southeast Asia. Tragulids inhabit dense vegetation and the males use their canines to effect close range visual cues for sexual identity, defense and/or display [11], [65]. Cooke [11], [65] hypothesized that a similar function might have been employed for the canines of *B. fangaroo*, which likewise lived in the dense understory of rainforests during the early-middle Miocene (see Archer et al. [28], [29]; Black et al. [66], p. 1017–1019, 1028–1030; Travouillon et al. [67], p. 34–35; and Travouillon et al. [68]; for a discussion of Riversleigh palaeoenvironments).

The late Oligocene balbarid *N. gillespieae* also appears to have possessed large canines, as evidenced by the size of the canine alveolus (see [12]). The upper canine of *B. nalima* is approximately half the size of that of the *B. fangaroo* paratype (QM F30456). It is not clear whether this contrasting length is diagnostic for *B. nalima*, plesiomorphic for Balbaridae or if it simply reflects sexual dimorphism. Certainly, Cooke [11] segregated the holotype (QM F36994) from the paratype of *B. fangaroo* as probably female versus male sexual morphs. This is consistent with the general trend towards larger canines, pronounced cranial crests (e.g., frontal and sagittal crests), and prominent processes (e.g., masseteric, postorbital processes) in male marsupials [69], [70]. These traits are also known to modify with ontogeny [71]. Although its teeth are fully erupted the patent cranial sutures of QM F36295 suggest a relatively young adult. Indeed, its dentition displays some of the smallest dimensional values recorded within our sample (Table 2). Based on this observed pattern, the comparatively reduced canines of the *B. nalima* holotype (QM F36295), together with its weakly developed frontal crests and postorbital processes, and the absence of sagittal crests, would presumably identify the individual as a female. Pointedly, bimodal distribution is not evident in our post canine tooth proportion plots for *B. nalima* (see Figs 3–4), which may argue against sex-based segregation. Nevertheless, these results are consistent with previous studies of craniodental variation in marsupials (e.g., *Perameles bougainville* [69]; *Thylacoleo carnifex* [72]; *Lestodelphys halli* [73]; *Diprotodon optatum* [74]; *Nimbadon lavarackorum* [57], *Phascolarctos cinereus* [75], *Macropus agilis* [76]), which indicate weaker manifestation of sexual dimorphism within cheek tooth parameters.

### Balbarid taxonomy

Cooke [11] reported either a protostylid and/or protostylid crest on the m1 of species referred to *Nambaroo* and *Wururoo* (unknown for *Galanarla*), and regarded this state as being plesiomorphic amongst Balbaridae. On the other hand, *Balbaroo* spp. are purportedly more derived in the loss of their protostylid, which was subsequently treated as a synapomorphy for this genus [11]. In contrast, our significantly larger sample of *B. fangaroo* specimens demonstrates a remnant protostylid crest in at least some individuals, including juvenile morphotypes (e.g., QM F19847 and QM F20038) erroneously identified as new species of *Wururoo* explicitly because of this feature (see [9]). Pointedly, the p3 and lower molar morphology of these examples is identical to that of *B. fangaroo* (Figure 6A–C), and falls within the estimated size range for this taxon (Table S1). Furthermore, some worn specimens undoubtedly referrable to *B. fangaroo* (QM F56981) manifest a wear facet in place of the protostylid crest, while others retain the structure even in heavily worn dentitions (QM F56289).

We also advocate synonymy of *Nambaroo bullockensis* [50] with *Balbaroo camfieldensis*. Schwartz and Megirian [50] originally assigned *N. bullockensis* to *Nambaroo* because of its weakly developed protostylid and protostylid crest on m1. Conversely, we found no evidence of a protostylid, nor any other discrete features that would warrant establishment of a distinct species. The confounding observation of a protostylid crest in many individuals of *B. fangaroo* thus renders this synapomorphy invalid for *Balbaroo*, and also brings into question the intuitive division of Balbaridae into the subfamilies Nambarinae and Balbarinae (*sensu* [2]), which was based primarily on the presence or absence of the m1 protostylid.

### Balbarid biostratigraphy

Our interpretation of the interspecific relationships within *Balbaroo* concur with current chronostratigraphical designations of faunal zones within the Riversleigh World Heritage Area [8], [28], [29], [32], [33], [77] and the Bullock Creek Local Fauna [32], [53], [78], despite the absence of robust support for the clade. For example, the potential placement of *Balbaroo gregoriensis* as the plesiomorphic sister taxon to all other *Balbaroo* spp. complies with its occurrence in one of the late Oligocene Faunal Zone A deposits, G Site [8], [28], [29], [32], [33], [77]. An as yet unnamed species of *Balbaroo* is also recorded in the late Oligocene Kangaroo Well Local Fauna of the Northern Territory [1], [78]. This specimen was not, however, available for study.

*Balbaroo fangaroo* is restricted to early Miocene Faunal Zone B LFs in the Riversleigh World Heritage Area, while both *Balbaroo nalima* and *Balbaroo camfieldensis* occur in middle Miocene Faunal Zone C LFs and the Bullock Creek LF respectively. As the seemingly most derived members of the genus, these latter taxa support assertions of faunal correlation between these assemblages [28], [77], [79], which was previously corroborated by coeval diprotodontids, palorchestids and thylacoleonids [22], [54], [56]–[58], [79]–[82].

With our proposed synonymy of *Nambaroo bullockensis* and *B. camfieldensis,* the last appearance of the *Nambaroo* lineage is thus confined to early Miocene assemblages where they lived alongside other more advanced balbarid taxa such as the species of *Ganawamaya* and *B. fangaroo.* No balbarids have so far been reported from late Miocene sequences, inferring probable extinction of the radiation sometime around the middle-late Miocene boundary. This transition is often linked with marked change in ecosystem structure (e.g., increasingly open vegetation), coupled to increasing aridity on the Australian landmass [83], [84]. Concomitant faunal turnover has also been proposed with many rainforest-inhabiting taxa such as the arboreal diprotodontid *Nimbadon lavarackorum* and the phascolarctids *Nimiokoala* and *Litokoala*, as well as enigmatic archaic lineages including yalkaparadontid, miralinid and pilkipildrid possums and yaralid bandicoots, disappearing from the fossil record before the late Miocene, followed by subsequent diversification of relatively more derived dasyurids, peramelids, thylacomyids and macropodine kangaroos [29], [55], [66], [85]–[92]. Growing molecular evidence, however, does not necessarily concur (see [14], [93]). Adding to the uncertainty is the rarity and relative paucity of the Australian late Miocene fossil record, which has significantly impaired our understanding of changes in diversity during this critical time in Australia's history. Thus, the interplay of lineage extinction and radiation might have been more complex and requires further investigation to test and refine these perceived distributional patterns.

## Supporting Information

Table S1 **Measurements (in mm) of the lower dentition of type and referred material of** ***Balbaroo fangaroo*** **from the Riversleigh World Heritage Area, Australia.**
(DOC)

Table S2 **Measurements (in mm) of the upper dentition of type and referred material of** ***Balbaroo fangaroo*** **from the Riversleigh World Heritage Area, Australia.**
(DOC)

Table S3 **Univariate statistics of type and referred material of** ***Balbaroo nalima*** **sp. nov from the Riversleigh World Heritage Area, Australia.**
(DOC)

Table S4 **Univariate statistics of type and referred material of** ***Balbaroo fangaroo*** **from the Riversleigh World Heritage Area, Australia.**
(DOC)

Table S5 **Measurements (in mm) of the lower and upper dentition of select species of** ***Balbaroo*** **,** ***Nambaroo*** **and** ***Wururoo*** **used in this study.**
(DOC)

Table S6 ***Balbaroo nalima*** **sp. nov. postcranial skeletal dimensions.**
(DOCX)

Table S7 **Character-taxon matrix used for phylogenetic analysis of Macropodiformes.**
(DOCX)

List S1 **Characters used in the phylogenetic analysis based on those of Kear and Pledge (2008).**
(DOCX)
