# Supplementary material for: A New Species of the Basal “Kangaroo” Balbaroo and a Re-Evaluation of Stem Macropodiform Interrelationships
Source: PLoS One. 2014 Nov 19;9(11):e112705. doi: 10.1371/journal.pone.0112705 (PMC4237356; doi:10.1371/journal.pone.0112705)
Supplement: Table S1 — Measurements (in mm) of the lower dentition of type and referred material of Balbaroo fangaroo from the Riversleigh World Heritage Area, Australia. (DOC) [file pone.0112705.s001.doc]

**Table S1. Measurements (in mm) of the lower dentition of type and referred material of *Balbaroo fangaroo* from the Riversleigh World Heritage Area, Australia.** Abbreviations: L, anteroposterior length; AW, anterior width; PW, posterior width; dp, deciduous premolar; p, premolar; m , molar. Riversleigh Site name abbreviations: Boid, Boid; BSE, Boid Site East; CK, Cadbury’s Kingdom; CS, Camel Sputum; CR, Creaser’s Ramparts; DT, Dirk’s Towers; Inab, Inabeyance; JH, Judith’s Horizontalis; MIM, Margo’s Immense Might; MM, Mike’s Menagerie; NG, Neville’s Garden; Outa, Outasite; PFI, Phil Found It; PIR, Price Is Right; QL, Quantum Leap; RSO, Ross Scott Orr; Souv, Souvenir; Up, Upper; WW, Wayne’s Wok.

| Specimen | Site | p2 | | dp3 | | p3 | | m1 | | | m2 | | | m3 | | | m4 | | |
| --- | --- | --- | --- | --- | --- | --- | --- | --- | --- | --- | --- | --- | --- | --- | --- | --- | --- | --- | --- |
| QM |  | L | W | L | W | L | W | L | AW | PW | L | AW | PW | L | AW | PW | L | AW | PW |
| F20616 | Boid |  |  |  |  |  |  | 7.18 | 4.95 | 5.58 | 7.55 | 5.58 | 5.54 |  |  |  |  |  |  |
| F56980 | BSE |  |  |  |  | 9.36 | 5.9 | 7.1 | 4.79 | 5.09 |  |  |  |  |  |  |  |  |  |
| F56294 | CK |  |  |  |  | 8.25 | 4.88 |  |  |  |  |  |  |  |  |  |  |  |  |
| F19806 | CS |  |  |  |  |  |  |  |  |  | 7.41 | 4.94 | 4.84 | 7.39 | 5.14 | 4.98 | 7.34 | 5.22 | 4.91 |
| F19867 | CS |  |  |  |  |  |  |  |  |  | 7.89 | 5.17 | 4.97 | 7.32 | 5.79 |  |  |  |  |
| F56976 | CS |  |  |  |  | 8.98 | 5.33 | 7.25 | 4.76 | 5.25 | 7.02 | 5.4 | 5.21 | 7.16 | 5.25 | 5.28 | 6.93 | 5.3 | 5.02 |
| F20085 | CS |  |  |  |  |  |  | 7.39 | 4.54 | 5.5 | 7.44 | 5.46 | 5.51 | 7.64 | 5.65 | 5.52 | 7.86 | 5.61 | 5.33 |
| F19697 | CS |  |  |  |  | 8.94 | 5.49 |  |  |  |  |  |  |  |  |  |  |  |  |
| F23480 | CS |  |  |  |  |  |  | 7.11 | 4.34 | 4.76 | 7.34 | 5.1 | 5.23 |  |  |  |  |  |  |
| F19865 | CS |  |  |  |  |  |  |  |  |  |  |  |  | 7.65 | 5.15 | 5.35 | 7.98 |  | 4.9 |
| F19608 | CS |  |  |  |  |  |  |  |  |  |  |  |  | 7.51 | 5.62 | 5.37 | 7.65 | 5.64 | 5.15 |
| F20283 | CS |  |  |  |  |  |  |  |  |  | 7.04 | 5.27 | 4.83 | 7.46 | 5.51 | 4.92 |  |  |  |
| F20024 | CS |  |  |  |  |  |  | 6.88 | 4.35 | 5.25 | 7.49 | 5.39 | 5.42 | 7.02 | 5.27 | 5.44 |  |  |  |
| F20247 | CS |  |  |  |  |  |  |  |  |  | 6.9 | 5.19 | 5.08 | 7.32 | 5.33 | 4.87 | 7.55 | 5.29 | 4.67 |
| F20251 | CS |  |  |  |  |  |  | 7.72 | 4.74 | 5.01 | 7.89 | 5.24 | 4.92 | 7.66 | 5.22 | 4.74 | 8.01 | 5.14 | 4.51 |
| F56288 | CS |  |  |  |  |  |  | 6.64 | 4.41 | 4.77 | 6.65 | 4.88 | 4.86 | 6.89 | 4.88 | 4.97 | 7.53 | 4.9 | 4.4 |
| F20606 | CS |  |  |  |  | 8.88 | 5.14 |  |  |  |  |  |  |  |  |  |  |  |  |
| F56984 | CS | 3.38 | 3.5 | 5.42 | 3.74 |  |  | 7.22 | 4.45 | 4.82 |  |  |  |  |  |  |  |  |  |
| F20071 | CS |  |  |  |  | 8.7 | 5.03 | 6.85 | 4.69 | 5.05 | 7.52 | 5.12 | 4.9 |  |  |  |  |  |  |
| F20245 | CS |  |  |  |  |  |  | 7.09 |  | 4.99 |  |  |  |  |  |  |  |  |  |
| F20629 | CR |  |  |  |  | 8.9 | 4.97 | 6.9 | 4.68 | 5.62 | 6.96 | 5.62 | 5.66 | 7.05 | 5.57 | 5.36 | 7.23 | 5.16 | 4.66 |
| F56287 | CR |  |  |  |  |  |  |  |  |  | 7.02 |  | 5 | 6.91 | 5.29 | 4.97 |  |  |  |
| F31401 | DT |  |  |  |  |  |  |  |  |  | 6.46 |  | 4.87 | 6.96 | 5.09 | 4.86 | 6.77 | 4.91 | 4.46 |
| F20610 | DT |  |  |  |  | 9.12 | 5.05 | 6.84 | 4.33 | 5.02 | 6.79 | 4.76 | 5.06 | 6.91 | 5.01 | 5.08 | 7.25 | 4.94 | 4.64 |
| F24518 | Inab |  |  |  |  |  |  | 7.15 | 4.66 | 5.23 | 7.37 | 5.15 | 5.03 | 7.43 | 5.68 | 5.39 |  |  |  |
| F56297 | JH |  |  |  |  | 9.38 | 5.81 |  |  |  |  |  |  |  |  |  |  |  |  |
| F30456 | MIM |  |  |  |  | 9.06 | 5.35 | 7.11 | 4.27 | 5.09 | 6.91 | 4.96 | 5.26 | 7.12 | 5.28 | 5.19 | 7.4 | 5.35 | 4.93 |
| F20277 | MM |  |  |  |  |  |  |  |  |  |  |  |  | 7.17 | 5.4 | 5.14 |  |  |  |
| F56290 | NG |  |  |  |  |  |  |  |  |  |  |  |  |  |  |  | 6.35 | 4.54 | 4.4 |
| F56289 | NG |  |  |  |  | 8.91 | 5.23 | 6.81 | 4.24 | 4.83 | 7.18 | 4.67 | 5.27 | 7.58 | 5.18 | 5.61 | 7.72 | 5.33 | 5.46 |
| F31448 | NG |  |  |  |  |  |  |  |  |  | 6.72 | 4.94 | 5.51 | 7.31 | 5.18 | 5.33 |  |  |  |
| F24614 | NG |  |  |  |  |  |  |  |  |  | 6.73 | 4.75 | 5.17 | 6.86 | 5.07 | 5.03 | 6.9 | 5.04 | 4.7 |
| F56983 | NG |  |  |  |  | 9.42 | 5.94 | 7.22 | 4.98 | 5.29 |  |  |  |  |  |  |  |  |  |
| F36994 | Outa |  |  |  |  | 7.97 | 5.12 | 6.44 | 4.38 | 4.88 | 6.15 | 4.75 | 4.94 | 6.39 | 4.97 | 4.79 | 6.94 | 4.89 | 4.4 |
| F40048 | PFI |  |  |  |  | 9.27 | 5.82 |  |  |  |  |  |  |  |  |  |  |  |  |
| F56285 | PIR |  |  |  |  | 8.92 | 5.26 | 6.87 | 4.3 | 5.31 | 6.9 | 5.19 | 5.64 | 7.59 | 5.69 | 5.33 | 7.04 | 5.44 | 4.95 |
| F56285 | PIR |  |  |  |  | 9.13 | 5.28 | 6.76 | 4.44 | 5.1 | 6.76 | 5.24 | 5.57 | 7.32 | 5.62 | 5.6 | 7.08 | 5.63 |  |
| F56286 | PIR |  |  |  |  |  |  |  |  |  | 7.18 | 5.37 | 5.48 | 7.01 | 5.68 | 5.57 | 7.06 | 5.54 | 5.28 |
| F30427 | QL |  |  |  |  |  |  |  |  |  |  |  |  | 6.25 | 4.21 |  |  |  |  |
| F20038 | RSO | 4.09 | 3.89 | 5.6 | 4.68 |  |  | 7.67 | 4.98 | 5.41 |  |  |  |  |  |  |  |  |  |
| F19578 | RSO | 3.35 | 3.58 | 4.88 | 3.86 |  |  | 6.94 | 4.58 | 4.78 |  |  |  |  |  |  |  |  |  |
| F30869 | Souv |  |  |  |  | 9.16 | 5.16 |  |  |  |  |  |  |  |  |  |  |  |  |
| F20087 | Up |  |  |  |  | 8.6 | 4.71 | 6.95 | 4.49 | 5.07 | 7.2 | 5.85 | 5.55 | 6.81 | 5.96 | 5.26 | 7.29 | 5.01 | 4.73 |
| F19832 | WW |  |  |  |  |  |  |  |  |  |  |  |  |  |  |  |  |  |  |
| F20063 | WW | 4.1 | 3.49 | 5.73 | 3.58 | 8.97 |  | 6.94 | 4.76 | 5.41 | 7.38 | 5.39 | 5.44 | 6.97 | 5.43 | 4.77 |  |  |  |
| F19823 | WW |  |  |  |  |  |  |  |  |  | 7.25 | 5.1 | 5.09 | 7.2 | 5.24 | 5.05 | 7.16 | 5.1 | 4.72 |
| F56295 | WW |  |  |  |  |  |  | 6.75 | 3.96 | 4.39 |  |  |  |  |  |  |  |  |  |
| F19847 | WW | 3.82 | 3.46 | 5.22 | 3.68 | 8.37 |  |  |  |  |  |  |  |  |  |  |  |  |  |
| F56981 | WW |  |  |  |  | 8.51 | 5.53 | 7.22 | 4.62 | 5.3 | 7.15 | 5.27 | 5.18 | 7.43 | 5.59 | 5.5 |  |  |  |
| F20078 | WW |  |  |  |  |  |  | 6.4 | 4.51 | 5.1 |  |  |  |  |  |  |  |  |  |
| F56982 | WW |  |  |  |  | 9.05 | 5.17 | 7.2 | 4.67 | 5.19 | 7.39 | 5.36 | 5.2 |  |  |  |  |  |  |
| F56985 | WW |  |  |  |  | 8.24 |  | 7.25 | 4.76 | 5.08 |  |  |  |  |  |  |  |  |  |
| F19815 | WW |  |  |  |  | 9.19 | 5.29 |  | 4.55 |  |  |  |  |  |  |  |  |  |  |
| F20073 | WW |  |  |  |  | 8.15 | 5.17 | 7.04 | 4.62 | 5.1 | 7.03 | 4.87 | 5.28 | 7.63 | 5.21 | 5.41 |  |  |  |
| F57589 | WW |  |  |  |  | 8.54 | 5.0 | 6.61 | 4.39 | 4.68 | 6.69 | 5.2 | 5.03 | 6.98 | 5.4 | 4.96 | 7.36 | 5.24 | 4.51 |
| F57590 | WW |  |  |  |  |  |  | 7.17 | 4.71 | 5.3 | 7.1 | 5.44 | 5.34 | 7.26 | 5.6 | 5.31 | 7.39 | 5.12 | 4.77 |
| F57591 | WW | 3.49 | 3.49 | 5.03 | 4.0 | 8.11 |  | 6.53 | 4.5 | 4.7 | 6.96 | 4.85 | 5.03 |  |  |  |  |  |  |
