# Supplementary material for: A New Species of the Basal “Kangaroo” Balbaroo and a Re-Evaluation of Stem Macropodiform Interrelationships
Source: PLoS One. 2014 Nov 19;9(11):e112705. doi: 10.1371/journal.pone.0112705 (PMC4237356; doi:10.1371/journal.pone.0112705)
Supplement: Table S2 — Measurements (in mm) of the upper dentition of type and referred material of Balbaroo fangaroo from the Riversleigh World Heritage Area, Australia. (DOC) [file pone.0112705.s002.doc]

**Table S2 Measurements (in mm) of the upper dentition of type and referred material of *Balbaroo fangaroo* from the Riversleigh World Heritage Area, Australia**. Abbreviations: L, anteroposterior length; W, bucco-lingual width; DP, deciduous premolar; P, premolar; M, molar. Riversleigh Site name abbreviations: CK, Cadbury’s Kingdom; CS, Camel Sputum; Dirk’s Towers; MIM, Margan’s Immense Might; MPP, Mike’s Potato Patch; NG, Neville’s Garden; Outa, Outasite; R12C, R12C; RSO, Ross Scott Orr; Up, Upper; WW, Wayne’s Wok.

| Specimen | Site | DP3 | | P3 | | M1 | | M2 | | M3 | | M4 | |
| --- | --- | --- | --- | --- | --- | --- | --- | --- | --- | --- | --- | --- | --- |
| QM |  | L | W | L | W | L | W | L | W | L | W | L | W |
| F56291 | CK |  |  |  |  |  |  |  | 7.23 | 6.83 | 5.5 |  | 6.28 |
| F56292 | CK |  |  | 10.29 | 6.64 |  |  |  |  |  |  |  |  |
| F56292 | CK |  |  | 10.27 | 6.22 |  |  |  |  |  |  |  |  |
| F56293 | CK |  |  | 9.84 | 6.19 |  |  |  |  |  |  |  |  |
| F56293 | CK |  |  | 9.67 | 5.21 |  |  |  |  |  |  |  |  |
| F56975 | CS |  |  | 9.81 | 6.55 | 7.15 | 6.69 | 6.11 | 7.03 | 6.9 | 5.92 | 7.1 | 6.71 |
| F56974 | CS |  |  | 9.27 | 5.9 | 7.56 | 6.63 | 6.42 | 7.15 | 6.84 | 5.85 |  |  |
| F24089 | CS |  |  | 8.8 | 5.8 | 6.92 | 6.73 | 5.92 | 7.44 | 6.83 | 5.67 |  |  |
| F19638 | CS |  |  |  |  | 6.68 | 6.46 | 6.08 | 7.08 | 6.8 | 5.86 | 7.39 | 6.52 |
| F20273 | CS |  |  |  |  | 7.12 | 6.21 | 5.72 |  |  |  |  |  |
| F13096 | CS |  |  |  |  |  |  |  | 7.01 | 6.53 | 5.87 | 7 | 6.67 |
| F20102 | CS |  |  | 9.33 | 6.16 |  |  |  |  |  |  |  |  |
| F19983 | CS |  |  |  |  |  |  |  | 7.04 | 6.13 | 5.26 | 7.05 | 6.05 |
| F23477 | CS |  |  |  |  |  |  |  |  |  |  | 6.64 |  |
| F20162 | CS |  |  |  |  | 7.2 | 6.27 | 5.4 | 7.35 | 6.61 | 5.51 | 7.46 | 6.61 |
| F56977 | CS |  |  | 9.84 | 6.72 | 7.62 |  | 6.05 | 7.41 | 6.88 | 5.81 | 7.73 | 6.64 |
| F20025 | CS |  |  | 9.95 |  |  |  | 5.82 | 7.6 | 6.54 | 5.6 | 7.49 | 6.58 |
| F20287 | CS |  |  |  |  |  |  |  | 6.57 | 6.38 | 5.54 | 6.36 | 6.15 |
| F23488 | CS |  |  | 9.68 |  | 7.87 | 6.6 | 6.46 | 7.63 | 7.14 | 6.34 | 7.71 | 6.8 |
| F19971 | CS |  |  |  |  |  |  |  | 7.28 | 6.65 | 5.65 | 7.87 | 6.73 |
| F19676 | CS |  |  |  |  | 7.39 | 6.85 | 6.18 | 7.32 | 6.91 | 5.92 | 7.6 | 7 |
| F19678 | CS |  |  |  |  | 6.47 | 6.4 | 5.38 | 7.56 | 6.23 | 5.07 |  | 5.17 |
| F20314 | CS |  |  |  |  | 7.62 | 6.65 | 6.27 |  |  |  |  |  |
| F23484 | CS |  |  |  |  | 6.39 | 5.64 | 5.39 | 6.89 | 6 | 5.21 | 7.31 | 5.93 |
| F19863 | CS |  |  | 9.39 |  |  |  | 5.86 | 7.28 | 7.14 | 6.1 |  |  |
| F20576 | CS |  |  | 9.33 | 6.22 | 6.9 | 6.64 | 5.86 | 7.01 | 6.73 | 5.78 | 7.22 | 6.46 |
| F56978 | CS |  |  | 9.54 | 6.27 | 7.19 | 6.37 | 5.85 |  |  |  |  |  |
| F31601 | CS |  |  |  |  | 7.01 | 6.63 | 5.94 | 7.13 | 6.89 | 5.55 | 7.31 | 6.37 |
| F20367 | DT |  |  |  |  |  |  |  | 6.1 | 6.52 | 5.62 | 6.65 | 6.39 |
| F24524 | DT |  |  |  |  | 6.7 | 6.57 | 6.01 | 6.77 | 6.38 |  |  |  |
| F30456 | MIM |  |  | 9.48 | 6.36 | 7.1 | 6.66 | 6.04 | 7.31 | 6.93 | 6.02 | 7.4 | 6.98 |
| F30456 | MIM |  |  |  | 6.29 | 7.18 | 6.75 | 6.14 | 7.21 | 6.98 | 5.78 | 7.3 | 6.77 |
| F23605 | MPP |  |  |  |  | 6.87 | 6.59 | 6.62 | 6.94 | 6.7 | 6.43 | 7.09 | 7.09 |
| F13091 | NG |  |  |  |  |  |  |  |  |  |  | 7.35 | 6.42 |
| F36994 | Outa |  |  | 8.13 | 5.66 | 6.07 | 6.66 | 5.45 | 6.4 | 6.43 | 5.19 | 6.75 | 6.16 |
| F19671 | R12C |  |  |  |  | 6.11 | 6.08 | 5.14 | 6.38 | 6.18 |  |  |  |
| F20278 | RSO |  |  |  |  |  |  |  |  |  |  | 7.15 | 6.93 |
| F56296 | RSO |  |  |  |  |  |  |  | 7.39 | 7.2 | 6.18 | 7.83 | 6.95 |
| F20279 | Up |  |  | 9.6 | 5.76 | 7.32 | 6.63 | 6 | 7.41 | 6.66 | 5.66 | 7.5 | 6.41 |
| F19928 | Up |  |  |  |  |  |  |  |  |  |  |  |  |
| F19649 | Up |  |  |  |  |  |  |  |  |  |  | 7.38 | 6.86 |
| F19615 | WW |  |  |  |  |  |  |  |  |  |  |  |  |
| F19601 | WW |  |  |  |  |  |  |  |  |  |  | 7.18 | 6.24 |
| F19934 | WW |  |  |  |  | 6.75 | 6.41 | 6.05 | 7.38 | 6.54 | 6.12 |  |  |
| F24193 | WW |  |  | 9.05 | 5.5 | 6.63 | 6.2 | 5.4 | 6.71 | 6.67 | 5.37 |  |  |
| F56979 | WW |  |  | 9.37 | 5.92 | 7.02 | 6.24 | 5.75 | 7.15 | 6.2 | 5.48 | 7.01 | 6.28 |
| F20005 | WW | 5.03 | 4.77 | 9.36 |  | 7.24 | 6.48 | 6.12 |  |  |  |  |  |
| F39979 | WW |  |  | 9.66 | 5.37 |  |  |  |  |  |  |  |  |
| F31594 | WW | 5.41 | 4.56 |  |  | 6.67 | 5.96 | 5.75 | 7.4 | 6.52 | 5.62 |  |  |
| F36413 | WW |  |  |  |  | 7.34 |  |  | 7.75 | 7.34 | 6.62 | 7.18 | 7.03 |
| F36365 | WW |  |  |  |  | 7.24 | 6.36 | 5.85 | 7.37 | 6.68 | 5.97 | 7.65 | 6.75 |
