# Supplementary material for: A New Species of the Basal “Kangaroo” Balbaroo and a Re-Evaluation of Stem Macropodiform Interrelationships
Source: PLoS One. 2014 Nov 19;9(11):e112705. doi: 10.1371/journal.pone.0112705 (PMC4237356; doi:10.1371/journal.pone.0112705)
Supplement: Table S3 — Univariate statistics of type and referred material of Balbaroo nalima sp. nov from the Riversleigh World Heritage Area, Australia. (DOC) [file pone.0112705.s003.doc]

**Table S3. Univariate statistics of type and referred material of *Balbaroo nalima* sp. nov from the Riversleigh World Heritage Area, Australia**. Abbreviations: AW; anterior width; CV, Coefficient of Variation; L, anteroposterior length; m, lower molar; M, upper molar; Max, maximum measurement; Min, minimum measurement; N, number of specimens; p, lower premolar; P, upper premolar; PW, posterior width; SD, Standard Deviation; SE, Standard Error.

|  | N | Min | Max | Mean | SE |  | SD | CV |
| --- | --- | --- | --- | --- | --- | --- | --- | --- |
| p3L | 13 | 8.9 | 10.67 | 9.88 | 0.09 |  | 0.47 | 4.78 |
| p3W | 14 | 4.87 | 5.77 | 5.32 | 0.06 |  | 0.32 | 6.00 |
| m1L | 16 | 6.84 | 7.97 | 7.23 | 0.06 |  | 0.30 | 4.15 |
| m1AW | 15 | 4.39 | 5.1 | 4.72 | 0.04 |  | 0.21 | 4.36 |
| m1PW | 17 | 4.85 | 5.61 | 5.19 | 0.04 |  | 0.23 | 4.45 |
| m2L | 14 | 7.16 | 8.03 | 7.67 | 0.04 |  | 0.24 | 3.14 |
| m2AW | 15 | 5.05 | 5.87 | 5.45 | 0.05 |  | 0.25 | 4.53 |
| m2PW | 14 | 5.02 | 5.67 | 5.30 | 0.04 |  | 0.20 | 3.85 |
| m3L | 15 | 7.6 | 9.11 | 7.98 | 0.07 |  | 0.36 | 4.57 |
| m3AW | 15 | 5.18 | 6.51 | 5.72 | 0.06 |  | 0.32 | 5.54 |
| m3PW | 14 | 5.12 | 6.37 | 5.66 | 0.07 |  | 0.36 | 6.31 |
| m4L | 8 | 7.73 | 9.03 | 8.31 | 0.07 |  | 0.39 | 4.66 |
| m4AW | 8 | 5.33 | 6.09 | 5.62 | 0.05 |  | 0.25 | 4.48 |
| m4PW | 8 | 4.85 | 5.83 | 5.32 | 0.05 |  | 0.30 | 5.58 |
| P3L | 11 | 10.3 | 12.86 | 11.36 | 0.17 |  | 0.84 | 7.42 |
| P3W | 10 | 5.89 | 7.14 | 6.54 | 0.07 |  | 0.34 | 5.21 |
| M1L | 18 | 6.9 | 8.13 | 7.46 | 0.08 |  | 0.37 | 4.97 |
| M1AW | 16 | 6.16 | 7.53 | 6.81 | 0.09 |  | 0.43 | 6.27 |
| M1PW | 16 | 5.63 | 6.95 | 6.27 | 0.09 |  | 0.42 | 6.70 |
| M2L | 16 | 6.45 | 8.16 | 7.62 | 0.08 |  | 0.41 | 5.43 |
| M2AW | 15 | 6.51 | 7.5 | 6.94 | 0.06 |  | 0.30 | 4.37 |
| M2PW | 16 | 5.45 | 6.81 | 6.23 | 0.07 |  | 0.34 | 5.44 |
| M3L | 11 | 7.43 | 8.92 | 8.01 | 0.10 |  | 0.47 | 5.92 |
| M3AW | 11 | 6.32 | 7.64 | 6.92 | 0.08 |  | 0.38 | 5.44 |
| M3PW | 11 | 5.8 | 6.94 | 6.11 | 0.06 |  | 0.31 | 5.13 |
| M4L | 5 | 7.57 | 8.93 | 8.10 | 0.12 |  | 0.58 | 7.14 |
| M4AW | 5 | 5.99 | 7.45 | 6.51 | 0.11 |  | 0.55 | 8.48 |
| M4PW | 5 | 4.7 | 6.13 | 5.16 | 0.12 |  | 0.60 | 11.59 |
