# Supplementary material for: A New Species of the Basal “Kangaroo” Balbaroo and a Re-Evaluation of Stem Macropodiform Interrelationships
Source: PLoS One. 2014 Nov 19;9(11):e112705. doi: 10.1371/journal.pone.0112705 (PMC4237356; doi:10.1371/journal.pone.0112705)
Supplement: Table S4 — Univariate statistics of type and referred material of Balbaroo fangaroo from the Riversleigh World Heritage Area, Australia. (DOC) [file pone.0112705.s004.doc]

**Table S4. Univariate statistics of type and referred material of *Balbaroo fangaroo* from the Riversleigh World Heritage Area, Australia**. Abbreviations: AW; anterior width; CV, Coefficient of Variation; L, anteroposterior length; m, lower molar; M, upper molar; Max, maximum measurement; Min, minimum measurement; N, number of specimens; p, lower premolar; P, upper premolar; PW, posterior width; SD, Standard Deviation; SE, Standard Error.

|  | N | Min | Max | Mean | SE | SD | CV |
| --- | --- | --- | --- | --- | --- | --- | --- |
| p3L | 25 | 7.97 | 9.42 | 8.86 | 0.06 | 0.41 | 4.59 |
| p3W | 22 | 4.71 | 5.94 | 5.30 | 0.04 | 0.33 | 6.22 |
| m1L | 30 | 6.4 | 7.72 | 7.03 | 0.04 | 0.30 | 4.23 |
| m1AW | 30 | 3.96 | 4.98 | 4.56 | 0.03 | 0.24 | 5.18 |
| m1PW | 30 | 4.39 | 5.62 | 5.11 | 0.04 | 0.27 | 5.27 |
| m2L | 31 | 6.15 | 7.89 | 7.12 | 0.05 | 0.39 | 5.43 |
| m2AW | 29 | 4.67 | 5.85 | 5.17 | 0.04 | 0.29 | 5.52 |
| m2PW | 31 | 4.83 | 5.66 | 5.21 | 0.04 | 0.26 | 5.04 |
| m3L | 31 | 6.25 | 7.66 | 7.19 | 0.05 | 0.35 | 4.92 |
| m3AW | 31 | 4.21 | 5.96 | 5.33 | 0.05 | 0.34 | 6.32 |
| m3PW | 29 | 4.74 | 5.61 | 5.20 | 0.04 | 0.27 | 5.15 |
| m4L | 21 | 6.35 | 8.01 | 7.29 | 0.06 | 0.42 | 5.75 |
| m4AW | 20 | 4.54 | 5.64 | 5.20 | 0.04 | 0.29 | 5.63 |
| m4PW | 20 | 4.4 | 5.46 | 4.81 | 0.04 | 0.32 | 6.64 |
| P3L | 21 | 8.13 | 10.29 | 9.51 | 0.07 | 0.48 | 5.03 |
| P3W | 18 | 5.21 | 6.72 | 6.04 | 0.06 | 0.43 | 7.12 |
| M1L | 29 | 6.07 | 7.87 | 7.01 | 0.06 | 0.44 | 6.22 |
| M1AW | 27 | 5.64 | 6.85 | 6.46 | 0.04 | 0.27 | 4.24 |
| M1PW | 30 | 5.14 | 6.62 | 5.90 | 0.05 | 0.35 | 5.88 |
| M2L | 34 | 6.1 | 7.75 | 7.14 | 0.05 | 0.38 | 5.26 |
| M2AW | 34 | 6 | 7.34 | 6.67 | 0.04 | 0.32 | 4.78 |
| M2PW | 32 | 5.07 | 6.62 | 5.75 | 0.05 | 0.36 | 6.28 |
| M3L | 29 | 6.36 | 7.87 | 7.26 | 0.05 | 0.36 | 4.99 |
| M3AW | 30 | 5.17 | 7.09 | 6.53 | 0.06 | 0.40 | 6.18 |
| M3PW | 29 | 4.98 | 6.14 | 5.54 | 0.04 | 0.31 | 5.68 |
| M4L | 15 | 6.18 | 7.91 | 6.89 | 0.07 | 0.47 | 6.80 |
| M4AW | 15 | 5.39 | 6.61 | 5.97 | 0.05 | 0.34 | 5.68 |
| M4PW | 15 | 4.1 | 5.28 | 4.63 | 0.04 | 0.31 | 6.79 |
