# Supplementary material for: A New Species of the Basal “Kangaroo” Balbaroo and a Re-Evaluation of Stem Macropodiform Interrelationships
Source: PLoS One. 2014 Nov 19;9(11):e112705. doi: 10.1371/journal.pone.0112705 (PMC4237356; doi:10.1371/journal.pone.0112705)
Supplement: Table S5 — Measurements (in mm) of the lower and upper dentition of select species of Balbaroo , Nambaroo and Wururoo used in this study. (DOC) [file pone.0112705.s005.doc]

**Table S5. Measurements (in mm) of the lower and upper dentition of select species of *Balbaroo*, *Nambaroo* and *Wururoo* used in this study.** Abbreviations: AW, anterior width; dp, deciduous premolar; L, anteroposterior length; m, molar; p, premolar; PW, posterior width. Measurements for: NMVP165000 (*B. camfieldensis*) and CPC7349 (*Balbaroo* sp.) from Flannery et al. (1983); NTM P9464-215, NTM P87110-26 and QVM:2000:GFV:18 (*N. bullockensis*) from Schwartz and Megirian (2004).

| Taxon | Specimen | Locality | p3 |  | m1 | | | m2 | | | m3 | | | m4 | | |
| --- | --- | --- | --- | --- | --- | --- | --- | --- | --- | --- | --- | --- | --- | --- | --- | --- |
|  |  |  | L | W | L | AW | PW | L | AW | PW | L | AW | PW | L | AW | PW |
| *Balbaroo camfieldensis* | CPC22179 | Small Hills Locality  (Bullock Creek LF) |  |  | 6.99 | 3.47 | 4.63 | 7.72 | 5.31 | 5.09 | 7.81 | 5.1 | 5.08 | 8.08 | 4.85 |  |
|  | NMVP165000 | Horseshoe West Locality  (Bullock Creek LF) |  |  |  |  |  | 8.2 |  |  | 8.6 | 5.52 | 5.59 |  | 5.8 | 5.5 |
|  |  |  |  |  |  |  |  |  |  |  |  |  |  |  |  |  |
| *Balbaroo gregoriensis* | CPC22186 | G-Site  (Riversleigh FZA) |  |  | 6.92 | 3.88 | 4.24 |  |  |  |  |  |  |  |  |  |
|  |  |  |  |  |  |  |  |  |  |  |  |  |  |  |  |  |
| *Balbaroo* sp. | CPC7349 | Kangaroo Well LF |  |  |  |  |  |  |  |  |  |  |  | 7.9 | 4.9 | 4.7 |
|  |  |  |  |  |  |  |  |  |  |  |  |  |  |  |  |  |
| *Wururoo dayamayi* | QM F19820 | White Hunter Site  (Riversleigh FZA) | 8.53 | 4.83 | 6.13 | 4.31 | 4.56 | 6.24 | 4.58 | 4.62 | 6.81 | 5.15 | 4.82 | 6.74 | 4.62 | 4.06 |
|  |  |  |  |  |  |  |  |  |  |  |  |  |  |  |  |  |
| *Nambaroo bullockensis* | NTM P991-24 | Blast Site  (Bullock Creek LF) |  |  | 6.86 | 3.57 | 4.32 | 7.84 | 4.59 | 4.97 | 8.12 | 5.21 | 5.18 | 8.69 | 5.48 | 5.16 |
|  | QVM:2000:GFV:18 | Unrecorded quarry  (Bullock Creek LF) |  |  |  |  |  |  |  |  | 7.5 | 5.1 | 5.2 |  |  |  |
|  |  |  |  |  |  |  |  |  |  |  |  |  |  |  |  |  |
|  |  |  |  |  |  |  |  |  | M2 |  |  | M3 |  |  |  |  |
|  |  |  |  |  |  |  |  | L | AW | PW | L | AW | PW |  |  |  |
|  |  |  |  |  |  |  |  |  |  |  |  |  |  |  |  |  |
| *Nambaroo bullockensis* | NTM P9464-215 | Top Site  (Bullock Creek LF) |  |  |  |  |  | 7.6 | 4.8 | 4.5 |  |  |  |  |  |  |
|  | NTM P87110-26 | Top Site  (Bullock Creek LF) |  |  |  |  |  |  |  |  | 7.6 | 4.9 | 4.7 |  |  |  |
|  |  |  |  |  |  |  |  |  |  |  |  |  |  |  |  |  |
