# Supplementary material for: A New Species of the Basal “Kangaroo” Balbaroo and a Re-Evaluation of Stem Macropodiform Interrelationships
Source: PLoS One. 2014 Nov 19;9(11):e112705. doi: 10.1371/journal.pone.0112705 (PMC4237356; doi:10.1371/journal.pone.0112705)
Supplement: Table S6 — Balbaroo nalima sp. nov. postcranial skeletal dimensions. (DOCX) [file pone.0112705.s006.docx]

**Table S6. *Balbaroo nalima* sp. nov. postcranial skeletal dimensions.**

VERTEBRAE: QM F41234

Caudal

Centrum height: 5 mm

Centrum width: 8 mm

Max. length: 23 mm

Max. width: 10 mm

Lumbar

Centrum height: 11 mm

Centrum width: 9 mm

Cervical

Centrum height: 12 mm

Centrum width: 14 mm

Thoracic

Centrum height: 11 mm

Centrum width: 10 mm

CALCANEUM: QM F41234

Max. length: 36 mm

Max. width: 25 mm

Diameter tuber calcanei: 13 mm

Length tuber calcanei: 20 mm

Width across calcaneum-cuboid facet: 9 mm

ULNA: QM F58209

Max. length: 90 mm

Width coronoid process: 11 mm

FEMUR: QM F50468

Max. length: 190 mm

Shaft width: 18 mm

FIBULA: QM F57025

Max. length: 135 mm

Shaft max. width: 7 mm
