# Supplementary material for: A New Species of the Basal “Kangaroo” Balbaroo and a Re-Evaluation of Stem Macropodiform Interrelationships
Source: PLoS One. 2014 Nov 19;9(11):e112705. doi: 10.1371/journal.pone.0112705 (PMC4237356; doi:10.1371/journal.pone.0112705)
Supplement: Table S7 — Character-taxon matrix used for phylogenetic analysis of Macropodiformes. (DOCX) [file pone.0112705.s007.docx]

**Table S7. Character-taxon matrix used for phylogenetic analysis of Macropodiformes.** Abbreviations: '?' signifies 'missing data'. Polymorphic states indicated by: **A**, (1,2); **B**, (0,1,2)

Trichosurus vulpecula 0 0 0 1 1 0 0 0 0 1 0 0 1 0 0 0 0 0 0 2 0 0 0 0 1 0 0 0 0 0 1 1 0 0 0 1 2 2 1 0 0 1 1 0 0 1 0 0 0 0 0 0 0 0 0 0 0 0 0 0 0 0 0 0 0 0 0 0 0 0 0 0 0 0 0 0 0 0 0 0 0 0 0 0 0 0 0 0 0 0 0 0 0 0 0 0 0 0 0 0 0 0 0 0 0 0 0 0

Balbaroo camfieldensis ? ? ? ? ? ? ? ? ? ? ? 0 ? ? 2 1 1 ? ? ? ? ? ? 2 1 ? ? 1 1 1 2 0 1 ? ? ? ? ? ? ? ? ? ? ? ? ? ? ? ? ? ? ? ? ? ? ? ? ? ? ? ? ? ? ? ? ? ? ? ? ? ? ? ? ? ? ? ? ? ? ? ? ? ? ? ? ? ? ? ? ? ? ? ? ? ? ? ? ? ? ? ? ? ? ? ? ? ? 0

Balbaroo fangaroo 0 0 1 1 0 0 1 0 1 0 0 0 0 1 2 1 0 0 0 1 0 0 0 2 1 1 0 0 1 1 A 0 1 0 1 0 1 2 1 1 1 1 1 0 1 0 0 ? ? ? ? ? ? ? ? ? ? ? ? ? ? ? ? ? ? ? ? ? ? ? ? ? ? ? ? ? ? ? ? ? ? ? ? ? ? ? ? ? ? ? ? ? ? ? ? ? ? ? ? ? ? ? ? ? 1 1 2 0

Balbaroo gregoriensis ? ? ? ? ? ? ? ? ? ? ? ? ? ? ? ? ? ? ? ? ? ? ? 2 ? ? ? 0 0 1 2 0 1 ? ? ? ? ? ? ? ? ? ? ? ? ? ? ? ? ? ? ? ? ? ? ? ? ? ? ? ? ? ? ? ? ? ? ? ? ? ? ? ? ? ? ? ? ? ? ? ? ? ? ? ? ? ? ? ? ? ? ? ? ? ? ? ? ? ? ? ? ? ? ? ? ? ? 0

Balbaroo nalima 0 0 1 0 0 0 1 0 1 0 0 0 0 1 2 1 0 0 1 1 0 1 1 2 1 1 0 1 1 2 B 0 1 0 1 1 1 2 1 1 1 1 1 0 1 0 0 ? ? ? ? ? ? ? 0 0 0 0 0 ? ? ? ? ? ? ? ? ? ? ? ? ? ? ? ? ? ? ? ? ? ? ? ? ? ? 0 ? 0 ? ? ? ? ? ? ? ? ? ? 0 0 ? ? 0 1 1 1 2 0

Bettongia moyesi ? 0 0 0 0 0 0 ? ? ? 0 ? 0 1 1 1 1 0 0 1 0 1 1 1 0 0 0 1 1 2 2 1 1 1 0 1 0 2 0 0 1 0 1 0 ? ? 0 ? ? ? ? ? ? ? ? ? ? ? ? ? ? ? ? ? ? ? ? ? ? ? ? ? ? ? ? ? ? ? ? ? ? ? ? ? ? ? ? ? ? ? ? ? ? ? ? ? ? ? ? ? ? ? ? ? ? 1 3 0

Bettongia penicillata 1 0 0 0 0 0 0 1 0 1 1 0 0 1 0 1 1 0 1 1 0 1 1 1 0 0 0 1 1 2 2 1 1 1 0 1 0 2 0 0 1 0 2 0 1 0 0 0 0 0 1 1 0 0 1 0 0 0 0 0 1 1 1 1 0 1 0 ? 1 1 1 0 1 0 0 1 0 0 0 0 0 0 0 0 0 1 1 1 0 0 0 0 0 0 0 0 1 0 0 0 0 1 1 0 0 1 2 0

Bulungamaya delicata 0 1 0 0 0 0 0 ? ? 1 1 ? 0 1 1 1 0 1 0 1 ? 1 1 2 ? 1 0 1 1 2 2 1 2 1 0 1 0 1 0 1 1 1 1 1 1 1 0 ? ? ? ? ? ? ? ? ? ? ? ? ? ? ? ? ? ? ? ? ? ? ? ? ? ? ? ? ? ? ? ? ? ? ? ? ? ? ? ? ? ? ? ? ? ? ? ? ? ? ? ? ? ? ? ? ? ? 1 2 0

Dendrolagus lumholtzi 0 0 1 0 1 1 0 1 0 0 1 1 0 2 2 1 1 1 1 1 0 1 1 2 1 1 0 1 1 2 2 1 2 0 0 0 1 2 1 1 0 1 1 1 1 0 0 0 0 0 1 0 1 0 0 0 1 0 0 0 0 1 1 1 1 1 1 1 1 1 1 0 0 0 0 1 0 0 1 1 0 0 0 0 0 0 1 0 0 1 1 1 0 0 1 0 1 1 0 0 1 0 1 0 1 1 1 0

Dorcopsis muelleri 1 0 0 0 0 1 0 1 0 0 0 1 0 2 2 1 1 1 1 1 0 1 1 3 1 1 0 1 1 2 2 1 2 0 0 0 2 2 1 1 0 2 1 ? 1 0 0 0 0 0 1 0 1 1 0 0 1 1 0 0 1 1 1 1 1 1 1 1 1 1 1 0 1 1 0 1 0 0 1 1 0 0 0 0 0 0 1 1 1 1 1 1 0 0 1 1 1 1 1 1 1 1 1 0 1 1 1 0

Dorcopsoides fossilis ? ? ? ? 1 ? ? ? 1 1 1 ? 0 1 1 1 1 ? ? 1 ? 1 0 2 1 1 0 1 1 2 2 0 2 0 0 0 0 1 1 1 0 1 0 0 ? ? 0 0 0 0 1 0 1 0 0 0 1 1 0 0 1 1 1 1 1 1 1 1 1 ? ? ? 1 1 0 1 0 0 0 0 0 0 0 0 0 ? ? 0 0 ? ? ? ? ? ? ? ? 1 ? ? ? ? 1 0 1 1 1 0

Ekaltadeta ima 0 0 1 1 0 0 1 ? 1 0 0 0 0 1 2 1 1 0 0 0 ? 0 0 0 0 0 0 1 2 1 0 1 1 1 1 1 2 2 1 0 0 1 0 0 1 0 0 ? ? ? ? ? ? ? ? ? ? ? ? ? ? ? ? ? ? ? ? ? ? ? ? ? ? ? ? ? ? ? ? ? ? ? ? ? ? ? ? ? ? ? ? ? ? ? ? ? ? ? ? ? ? ? ? ? 1 1 2 0

Ganawamaya acris ? ? ? ? 0 0 ? ? ? ? 0 0 0 1 1 1 1 1 0 1 ? 1 1 2 1 1 1 0 0 0 2 0 1 1 1 2 2 2 0 1 0 1 0 1 ? ? 0 ? ? ? ? ? ? ? ? ? ? ? ? ? ? ? ? ? ? ? ? ? ? ? ? ? ? ? ? ? ? ? ? ? ? ? ? ? ? ? ? ? ? ? ? ? ? ? ? ? ? ? ? ? ? ? ? ? 1 1 2 0

Ganguroo bilamina ? ? ? ? ? ? ? ? ? ? 1 0 0 1 1 1 0 1 0 1 ? 0 1 2 0 ? 1 1 1 2 2 1 2 0 0 ? ? ? ? ? ? ? 1 ? ? ? 0 ? ? ? ? ? ? ? ? ? ? ? ? ? ? ? ? ? 0 0 1 ? 0 ? ? ? ? ? ? ? ? ? ? ? ? ? ? ? ? ? ? 0 ? 1 0 1 1 1 0 1 1 0 0 0 0 1 1 0 1 1 2 0

Hadronomas puckridgi 1 1 1 0 0 0 0 ? ? ? 1 1 0 1 ? 1 1 1 1 1 ? 1 1 2 1 1 1 1 1 2 2 1 2 0 2 0 1 1 1 1 0 1 1 1 1 0 0 1 1 0 2 1 1 1 1 0 0 1 1 0 0 0 1 1 1 1 1 1 1 ? ? ? 1 1 1 1 1 0 0 1 1 1 1 1 1 1 1 1 0 0 1 0 ? ? ? 0 ? 1 ? 0 ? 1 1 0 1 1 2 1

Hypsiprymnodon bartholomaii 0 ? ? ? 0 0 0 0 0 1 0 ? ? ? ? ? ? ? ? ? ? ? 1 0 ? 2 ? ? ? ? ? ? ? ? ? 1 2 2 1 0 0 1 ? 0 1 0 ? ? ? ? ? ? ? ? ? ? ? ? ? ? ? ? ? ? ? ? ? ? ? ? ? ? ? ? ? ? ? ? ? ? ? ? ? ? ? ? ? ? ? ? ? ? ? ? ? ? ? ? ? ? ? ? ? ? ? ? ? ?

Hypsiprymnodon moschatus 0 0 0 0 0 0 1 0 0 1 0 0 0 1 1 1 1 0 0 0 0 0 0 0 0 2 0 1 2 1 0 1 1 1 0 1 2 2 1 0 0 1 0 0 1 0 0 0 0 0 1 0 0 0 0 0 0 0 0 0 0 0 1 0 0 0 0 1 1 0 0 0 1 0 0 0 0 0 0 0 0 0 0 0 0 0 1 0 1 0 0 0 0 1 0 1 0 0 0 0 0 0 0 0 1 1 2 0

Macropus rufus 1 0 0 0 1 1 0 1 0 0 1 1 0 2 2 1 1 1 1 1 1 1 1 3 1 2 1 1 1 2 2 1 2 0 0 0 2 2 1 1 0 0 0 1 1 0 0 0 0 0 1 1 1 1 1 1 1 1 0 0 1 1 1 1 1 1 1 1 1 1 1 0 1 1 0 1 0 0 1 1 0 0 0 0 0 1 1 1 0 1 1 1 0 0 1 0 0 1 1 1 1 1 1 0 1 1 1 0

Nambaroo gillespieae 0 0 1 1 0 0 1 0 1 0 0 0 0 1 1 1 0 0 0 1 0 0 0 2 0 1 0 0 0 0 0 0 1 0 1 0 2 2 1 1 0 1 0 0 1 0 0 0 0 1 1 0 0 0 0 0 0 0 0 0 0 0 1 0 0 0 0 1 0 0 0 1 1 0 0 0 0 0 0 0 0 0 0 0 0 0 1 0 1 0 0 0 ? ? 0 ? 0 0 0 0 0 1 0 1 1 1 2 0

Ngamaroo archeri ? ? ? ? ? ? ? ? ? ? 1 0 0 1 1 1 ? ? ? 1 ? ? 1 1 1 ? 1 1 1 2 2 1 1 ? ? ? ? ? ? ? ? ? ? ? ? ? ? ? ? ? ? ? ? ? 0 0 0 0 0 0 0 0 1 0 ? ? ? ? ? ? ? ? ? 0 0 0 ? ? ? ? ? ? ? ? ? 0 1 ? 0 ? ? ? ? 1 0 1 1 1 1 ? ? ? ? ? 0 2 3 0

Nowidgee matrix ? ? ? ? 0 0 ? ? ? ? 1 0 0 1 1 1 0 0 0 1 ? 0 1 1 0 0 0 1 1 2 2 1 1 0 0 1 0 2 0 1 1 1 1 ? ? ? 0 ? ? ? ? ? ? ? ? ? ? ? ? ? ? ? ? ? ? ? ? ? ? ? ? ? ? ? ? ? ? ? ? ? ? ? ? ? ? ? ? ? ? ? ? ? ? ? ? ? ? ? ? ? ? ? ? ? 1 1 2 0

Petrogale penicillata 1 0 0 0 1 1 0 1 0 1 1 1 0 2 2 1 1 1 1 1 1 1 1 3 1 2 0 1 1 2 2 1 2 0 0 2 2 2 1 1 0 0 0 1 1 2 0 0 0 0 1 1 1 1 0 0 1 1 0 0 1 1 1 1 1 1 1 1 1 1 1 0 1 1 0 1 0 0 1 1 0 0 0 0 0 1 1 1 0 1 1 1 0 0 1 0 0 1 1 1 1 1 1 0 1 1 1 0

Potorous tridactylus 0 0 0 0 0 0 0 ? ? ? 0 0 0 1 1 1 1 0 1 1 0 1 1 1 0 0 0 1 1 2 2 1 1 0 0 1 0 2 0 0 1 0 1 0 ? ? 0 0 0 ? 1 0 0 0 0 0 0 0 0 0 0 0 1 1 0 0 0 1 0 ? ? ? 1 0 0 0 0 0 0 0 0 0 0 0 0 ? ? 1 1 0 0 ? 0 1 1 0 0 0 0 0 1 1 0 ? 0 1 1 0

Procoptodon goliah 1 ? 1 0 ? 1 0 1 0 1 1 1 1 2 2 1 0 0 1 1 1 1 1 3 1 2 0 1 1 2 2 1 2 0 2 0 2 2 1 1 1 2 1 2 0 0 1 1 1 0 2 1 1 1 1 0 0 1 1 1 0 0 1 1 1 1 1 1 1 ? 1 0 1 1 1 1 1 1 0 1 1 1 1 1 1 1 1 1 0 1 1 0 0 1 1 0 1 1 1 1 1 ? 1 0 1 1 1 0

Propleopus oscillans ? ? ? ? 0 1 ? ? ? ? 0 0 0 1 1 1 1 0 0 1 0 0 0 1 0 0 0 0 0 1 1 1 1 1 0 1 2 2 0 1 0 0 0 0 ? ? 0 ? ? ? ? ? ? ? ? ? ? ? ? ? ? ? ? ? ? ? ? ? ? ? ? ? ? ? ? ? ? ? ? ? ? ? ? ? ? ? ? ? ? ? ? ? 1 1 0 ? 1 ? ? ? ? ? ? ? 1 1 2 0

Purtia mosaicus ? ? ? ? ? ? ? ? ? ? 0 0 ? 1 1 1 ? ? 1 1 ? 0 1 1 ? ? 1 1 1 2 2 1 1 1 0 ? ? ? ? ? ? ? 1 ? ? ? ? ? ? ? ? ? ? ? ? ? ? ? ? ? ? ? ? ? ? ? ? ? ? ? ? ? ? ? ? ? ? ? ? ? ? ? ? ? ? ? ? ? ? ? ? ? ? ? ? ? ? ? ? ? ? ? ? ? ? 2 3 1

Rhizosthenurus flanneryi 1 1 1 0 0 0 0 ? 0 0 1 1 0 1 1 1 1 1 1 1 0 1 1 2 1 1 1 1 1 2 ? 1 2 0 2 0 ? ? ? ? ? 1 1 1 1 0 0 0 0 0 2 1 1 1 0 0 0 1 1 0 0 0 1 1 1 1 1 1 1 ? ? ? 1 1 0 1 0 0 0 1 1 1 1 1 1 ? ? 1 0 ? ? ? 0 ? ? ? 1 ? ? 0 ? ? ? 0 1 1 2 1

Simosthenurus occidentalis 1 1 0 0 1 1 0 ? 0 0 1 1 1 2 2 1 0 0 1 1 1 1 1 3 1 2 0 1 1 2 2 1 2 0 2 0 2 2 1 2 0 2 1 2 1 0 1 1 1 0 2 1 1 1 1 0 0 1 1 1 0 0 1 1 1 1 1 1 1 ? 1 0 1 1 1 1 1 1 0 1 1 1 1 1 1 1 1 1 0 1 1 0 0 1 1 0 1 1 1 1 1 1 1 0 1 1 1 0

Thylogale thetis 1 0 0 1 1 1 0 1 0 0 0 1 0 2 2 1 1 1 1 1 1 1 1 3 1 1 0 1 1 2 2 1 2 0 0 2 2 2 1 1 0 1 1 1 1 0 0 0 0 0 1 0 1 1 0 0 1 1 0 0 1 1 1 1 1 1 1 1 1 1 1 0 1 1 0 1 0 0 1 1 0 0 0 0 0 1 1 1 0 1 1 1 0 0 1 0 0 1 1 1 1 1 1 0 1 1 1 0

Wabularoo naughtoni ? 1 0 0 0 0 0 ? ? 1 0 0 0 ? ? ? 0 ? 0 1 ? 0 1 2 ? 0 0 1 1 2 2 1 2 ? ? ? ? ? ? ? ? ? ? ? ? ? 0 ? ? ? ? ? ? ? ? ? ? ? ? ? ? ? ? ? ? ? ? ? ? ? ? ? ? ? ? ? ? ? ? ? ? ? ? ? ? ? ? ? ? ? ? ? ? ? ? ? ? ? ? ? ? ? ? ? 1 1 2 0

Wakiewakie lawsoni ? ? ? ? ? ? ? ? ? ? 1 0 0 1 1 1 0 0 ? 1 ? 1 1 1 0 ? 0 1 1 2 2 1 1 ? ? ? ? ? ? ? ? ? ? ? ? ? ? ? ? ? ? ? ? ? ? ? ? ? ? ? ? ? ? ? ? ? ? ? ? ? ? ? ? ? ? ? ? ? ? ? ? ? ? ? ? ? ? ? ? ? ? ? ? ? ? ? ? ? ? ? ? ? ? ? 0 2 3 1

Wallabia bicolor 1 0 0 0 1 1 0 1 0 0 1 1 0 2 2 1 1 1 1 1 1 1 1 3 1 2 0 1 1 2 2 1 2 0 0 2 2 2 1 1 0 1 0 1 0 0 0 0 0 0 1 1 1 0 0 1 1 1 0 0 1 1 1 1 1 1 1 1 1 1 1 0 1 1 0 1 0 0 1 1 0 0 0 0 0 1 1 1 0 1 1 1 0 0 1 0 0 1 1 1 1 1 1 0 1 1 1 0

Wanburoo hilarus ? ? ? ? ? 0 ? ? ? ? 1 1 0 1 1 1 0 1 ? 1 0 1 0 2 ? 1 0 1 1 2 2 1 2 0 0 1 1 2 0 1 1 1 1 1 0 ? ? ? ? ? ? ? ? ? ? ? ? ? ? ? ? ? ? ? ? ? ? ? ? ? ? ? ? ? ? ? ? ? ? ? ? ? ? ? ? ? ? ? ? ? ? ? ? ? ? ? ? ? ? ? ? ? ? ? 1 1 2 1

Wururoo dayamayi ? ? ? ? ? ? ? ? ? ? ? ? 0 1 ? ? 0 ? ? 1 ? 1 0 2 ? ? 0 0 0 1 1 0 1 ? ? ? ? ? ? ? ? ? 1 ? ? ? ? ? ? ? ? ? ? ? ? ? ? ? ? ? ? ? ? ? ? ? ? ? ? ? ? ? ? ? ? ? ? ? ? ? ? ? ? ? ? ? ? ? ? ? ? ? ? ? ? ? ? ? ? ? ? ? ? ? 1 1 2 0
