# Supplementary material for: A New Species of the Basal “Kangaroo” Balbaroo and a Re-Evaluation of Stem Macropodiform Interrelationships
Source: PLoS One. 2014 Nov 19;9(11):e112705. doi: 10.1371/journal.pone.0112705 (PMC4237356; doi:10.1371/journal.pone.0112705)
Supplement: List S1 — Characters used in the phylogenetic analysis based on those of Kear and Pledge (2008). (DOCX) [file pone.0112705.s008.docx]

**List S1. Characters used in the phylogenetic analysis based on those of Kear and Pledge (2008)**

**1.** **BASIOCCIPITAL/BASISPHENOID:**

0. COPLANAR

1. ANGLED

**2.** **FRONTAL REGION:**

0. NOT FLAT

1. FLAT

**3.** **FRONTAL SINUSES:**

0. NOT MARKEDLY INFLATED

1. MARKEDLY INFLATED

**4.** **POSTORBITAL CONSTRICTION OF SKULL:**

0. ABSENT

1. PRESENT

**5.** **MASSETERIC PROCESS:**

0. NOT EXTENDING BELOW ALVEOLAR MARGIN

1. EXTENDING BELOW ALVEOLAR MARGIN

**6.** **CHEEK REGION OF SKULL:**

0. SMOOTH TRANSITION OF ZYGOMA TO CHEEK

1. WITH SULCUS ANTERIOR TO ZYGOMA

**7.** **LATERAL WALL OF NEUROCRANIUM:**

0. ALISPHENOID-PARIETAL CONTACT

1. WITH SQUAMOSAL-FRONTAL CONTACT

**8.** **PAROCCIPITAL PROCESS-ALISPHENOID CONTRIBUTION:**

0. DOES NOT CONTRIBUTE

1. CONTRIBUTES TO ANTERIOR FACE

**9.** **MASTOID PROCESS:**

0. SMALL (<PAROCCIPITAL PROCESS)

1. HYPERTROPHIED (>PAROCCIPITAL PROCESS)

**10.** **INFLATED ALISPHENOID FORMING AUDITORY BULLA:**

0. ABSENT

1. PRESENT

**11.** **ALIGNMENT OF MOLAR DORSOVENTRAL AXES:**

0. CHANGING IN ANTEROPOSTERIOR DIRECTION

1. ALIGNED

**12.** **DIGASTRIC EMINENCE:**

0. CENTRALLY POSITIONED

1. POSTERIORLY POSITIONED

**13.** **MANDIBULAR SYMPHYSIS:**

0. NOT ANKYLOSED

1. ANKYLOSED

**14.** **ANTERIOR PENETRATION OF MASSETER (ADULT):**

0. MASSETER NOT INVADING MANDIBLE

1. PENETRATION ANTERIOR TO M_4_

2. PENETRATION NOT ANTERIOR TO M_4_

**15.** **MASSETERIC AND INFERIOR DENTAL CANALS:**

0. MASSETERIC CANAL NOT DEVELOPED

1. CANALS CONFLUENT ANTERIOR TO MASSETERIC FOSSA

2. CANAL PARTITIONED ANTERIOR TO THE MASSETERIC FOSSA

**16.** **MANDIBULAR CANAL:**

0. MASSETERIC FORAMEN ABSENT

1. DISTINCT CANAL SEPARATES MANDIBULAR AND MASSETERIC FORAMINA

2. CANAL LENGTH REDUCED, FORAMINA OVERLAP

3. CANAL LOST, SINGLE MANDIBULAR FORAMEN

**17.** **POSTERIOR MENTAL FORAMEN:**

0. PRESENT

1. ABSENT

**18.** **I_1_ OCCLUSAL SURFACE:**

0. REACHING TO LEVEL OF MOLAR OCCLUSAL PLANE

1. BELOW MOLAR OCCLUSAL PLANE

**19.** **CANINIFORM TOOTH (I_2_) PRESENT IMMEDIATELY POSTERIOR TO I_1_:**

0. PRESENT

1. ABSENT

**20.** **P_2_:**

0. PRESENT, NOT REDUCED, PERSISTS AFTER P_3_ ERUPTION

1. PRESENT, NOT REDUCED, DISPLACED BYP_3_ ERUPTION

2. REDUCED OR ABSENT

**21.** **C^1^:**

0. PRESENT

1. ABSENT

**22.** **P_3_:**

0. BUCCALLY FLEXED

1. ALIGNED WITH MOLAR ROW

**23.** **P^3^/P_3_:**

0. SHORT (<1.5M^1^/_1_)

1. ELONGATE (>1.5M^1^/_1_)

**24.** **MOLARS:**

0. BUNODONT

1. BUNOLOPHODONT

2. LOPHODONT, BRACHYODONT

3. LOPHODONT, HYPSODONT

**25.** **M_4_:**

0. MARKEDLY SMALLER

1. NOT MARKEDLY SMALLER THAN ANTERIOR MOLARS

**26.** **P^3^:**

0. LACKING POSTEROLINGUAL CUSP OR RIDGE

1. WITH POSTEROLINGUAL CUSP

2. WITH POSTEROLINGUAL RIDGE

**27.** **P_3_:**

0. CINGULIDS ABSENT

1. WITH DISTINCT BUCCAL AND/OR LINGUAL CINGULIDS

**28.** **M_1_ PROTOCONID:**

0. CENTRAL

1. BUCCAL

**29.** **M_1_ PROTOCONID:**

0. DOMINATES TRIGONID

1. SUBEQUAL TO METACONID

2. REDUCED

**30.** **TRIGONID BASIN ON M_1_:**

0. NOT DEVELOPED

1. NARROW TRANSVERSELY

2. BROAD TRANSVERSELY

**31.** **M_1_ PROTOSTYLID:**

0. PRESENT AS CUSPID

1. PRESENT AS ENAMEL RIDGE

2. ABSENT

**32.** **HYPOCINGULID ON LOWER MOLARS:**

0. PRESENT

1. ABSENT

**33.** **HYPOLOPHID FORMATION:**

0. HYPOLOPHID AND BUCCAL CRESTFROM ENTOCONID ABSENT

1. FORMED BY BUCCAL CREST FROMENTOCONID WITH POSTHYPOCRISTID LOW AND POSTERIORLY POSITIONED

2. FORMED BY ELEVATED POSTHYPOCRISTID, BUCCALCREST FROM ENTOCONID REDUCED OR LOST

**34.** **I_1_ DORSAL ENAMEL FLANGE:**

0. PRESENT

1. ABSENT

**35.** **I_1_ ENAMEL:**

0. BUCCAL SURFACE ONLY

1. BUCCAL AND DORSOLINGUAL SURFACES

2. BUCCAL AND LINGUAL SURFACES

**36.** **'FORELINK' (PREPROTOCRISTA) ON M^1^:**

0. ABSENT

1. PRESENT, DIAGONAL

2. BUCCALLY DISPLACED, ANTEROPOSTERIORLY DIRECTED

**37.** **M^1^ STYLAR CUSP C:**

0. PRESENT

1. REDUCED TO STYLAR CREST

2. ABSENT

**38.** **M^1^ STYLAR CUSP D:**

0. PRESENT

1. REDUCED TO STYLAR CREST

2. ABSENT

**39.** **M^1^ ANTEROLINGUAL CINGULUM:**

0. PRESENT

1. ABSENT

**40.** **M^1^ POSTPROTOCRISTA:**

0. CONTACTS METACONULE

1. CONTACTS METALOPH BUCCAL TO METACONULE

2. ABSENT

**41.** **M^1^ NEOMETACONULE AND 'POSTLINK':**

0. ABSENT

1. PRESENT

**42.** **POSTMETACRISTA-POSTMETACONULE CRISTA:**

0. SEPARATED

1. CONTINUOUS

2. OVERLAP

**43.** **MENTAL FORAMEN:**

0. ANTERIOR TO P_3_, WELL BELOW DIASTEMAL MARGIN

1. CLOSE TO P_3_, WELL BELOW DIASTEMAL MARGIN

2. CLOSE TO P_3_, CLOSE TO DIASTEMAL MARGIN

**44.** **P^3^ LINGUAL CINGULUM:**

0. ABSENT

1. PRESENT

2. PRESENT AND ELEVATED

**45.** **INTERNAL CAROTID FORAMEN:**

0. ANTERIOR TO SUTURE

1. CLOSE TO OR POSTERIOR TO BASIOCCIPITAL/BASISPHENOID SUTURE

**46.** **FORAMEN OVALE:**

0. LATERAL, FLOORED BY ALISPHENOID PROCESS FROM TYMPANIC WING

1. LATERAL, WITH NARROW VENTRAL ALISPHENOID PROCESS

2. MESIAL, WITH INCOMPLETE VENTRAL ALISPHENOID PROCESS

**47.** **OCCLUSAL EDGE OF I_1_:**

0. NOT SINUOUS

1. SINUOUS

**48.** **MEDIAL MALLEOLAR FOSSA OF ASTRAGALUS:**

0. LARGE AND OVOID

1. SMALL AND CIRCULAR

**49.** **MEDIAL MALLEOLAR PROCESS OF ASTRAGALUS:**

0. TRANSVERSELY BROAD AND LATERALLY FLARING

1. REDUCED AND TRANSVERSELY NARROW

**50.** **MALLEOLAR FOSSA BORDER OF ASTRAGALUS:**

0. SHORT AND NARROWING TOWARDS EXTREMITIES

1. ELONGATE AND UNIFORMLY THICK ALONG LENGTH

**51.** **TROCHLEAR RIDGES ON ASTRAGALUS:**

0. INDISTINCT WITH NO CLEAR ORIENTATION

1. OBLIQUELY ORIENTED RELATIVE TO LONG AXIS OF PES, GIVING RHOMBOIDAL OUTLINE

2. SUBPARALLEL TO LONG AXIS OF PES, GIVING RECTANGULAR DORSAL OUTLINE TO ARTICULAR FACET

**52.** **POSTERIOR EDGE OF LATERAL TROCHLEAR CREST OF ASTRAGALUS:**

0. CONTINUOUS

1. TRUNCATED

**53.** **ASTRAGALAR NECK:**

0. ELONGATE AND ANTERIORLY DIRECTED

1. SHORT AND ANTERIORLY DIRECTED

2. VERY SHORT AND PLANTARLY DIRECTED

**54.** **NAVICULAR FACET OF ASTRAGALUS:**

0. ELONGATE AND CONDYLE LIKE

1. ANTEROPOSTERIORLY SHORT AND ANTERIORLY DIRECTED

**55.** **SUSTENTACULUM TALI OF CALCANEUM:**

0. HORIZONTALLY ORIENTED AND TRANSVERSELY BROAD

1. STEEPLY INCLINED AND TRANSVERSELY NARROW

**56.** **TUBER CALCIS OF CALCANEUM:**

0. DORSOVENTRALLY DEEP AND POSTERIORLY FLARING

1. DORSOVENTALLY NARROW AND SUBCYLINDRICAL

**57.** **ASTRAGALUS-CALCANEUM FACET:**

0. BROAD WITH MEDIAL CONSTRICTION ABSENT

1. MEDIALLY CONSTRICTED OR COMPLETELY DIVIDED

**58.** **PLANTAR RUGOSITY ON THE CALCANEUM:**

0. ANTEROPOSTERIORLY SHORT WITH BROAD, SHALLOW PLANTAR SULCUS

1. ANTEROPOSTERIORLY LONG WITH PLANTAR SULCUS NARROW, DEEP, OR ABSENT

**59.** **LONG AXIS OF DORSOLATERAL FACET OF CALCANEUM:**

0. OBLIQUELY ORIENTED

1. DORSOVENTRALLY ORIENTED

**60.** **CUBOID BODY:**

0. NOT PROXIMODISTALLY COMPRESSED

1. PROXIMODISTALLY COMPRESSED

**61.** **MEDIAL PLANTAR TUBEROSITY OF CUBOID:**

0. WEAKLY DEVELOPED

1. WELL DEVELOPED, FORMING PROMINENT RAISED TUBERCLE

**62.** **GROOVE BETWEEN MEDIAN AND LATERAL PLANTAR TUBEROSITIES OF CUBOID:**

0. ABSENT

1. PRESENT

**63.** **CALCANEUM-CUBOID FACET:**

0. WEAKLY STEPPED AND SIGMOIDAL

1. DISTINCTLY STEPPED

**64.** **GROOVE BETWEEN DORSOMEDIAL AND VENTROMEDIAN FACETS OF CUBOID:**

0. ABSENT

1. PRESENT

**65.** **NAVICULAR BODY:**

0. CRESCENTIC IN LATERAL VIEW WITH VENTRAL EDGE LONGER THAN DORSAL

1. SUBRECTANGULAR IN LATERAL VIEW WITH DORSAL EDGE LONGER OR SUBEQUAL TO VENTRAL EDGE

**66.** **NAVICULAR PLANTAR EMINENCE:**

0. PROMINENT AND CONDYLE LIKE

1. REDUCED

**67.** **ASTRAGALAR FACET OF NAVICULAR:**

0. TRANSVERSELY NARROW AND DORSOVENTRALLY ELONGATE

1. TRANSVERSELY WIDE AND DORSOVENTRALLY SHORTENED

**68.** **MESOCUNEIFORM/NAVICULAR CONTACT:**

0. PRESENT

1. ABSENT

**69.** **CUBOID FACET OF NAVICULAR:**

0. DORSOVENTRALLY SHORT

1. DORSOVENTRALLY ELONGATE

**70.** **POSTERIOR NODE ON ENTOCUNEIFORM:**

0. PRESENT

1. ABSENT

**71.** **METATARSAL I:**

0. PRESENT

1. ABSENT

**72.** **METATARSAL I PROXIMAL FACET MARGIN:**

0. WITH PLANTAR LIP

1. WITHOUT PLANTAR LIP

**73.** **METATARSAL IV:**

0. SHORT AND ROBUST WITH TOTAL LENGTH < THREE TIMES TRANSVERSE WIDTH OF DISTAL FACET

1. LONG AND SLENDER WITH TOTAL LENGTH > THREE TIMES TRANSVERSE WIDTH OF DISTAL FACET

**74.** **PLANTAR CREST OF METATARSAL IV:**

0. ABSENT

1. PRESENT

**75.** **METATARSAL V FACET OF METATARSAL IV:**

0. CONTINUOUS WITH MEDIAN GROOVE ABSENT

1. SUBDIVIDED BY DISTINCT MEDIAN GROOVE

**76.** **CUNEIFORM FACET OF METATARSAL IV:**

0. CONVEX OR PLANAR

1. CONCAVE AND INSET

**77.** **INTRA-ARTICULAR GROOVE OF METATARSAL IV:**

0. LATERALLY OPENING

1. VENTRALLY OPENING

**78.** **PEDAL DIGIT V:**

0. WELL DEVELOPED

1. REDUCED

**79.** **LATERAL TUBEROSITY OF METATARSAL V:**

0. WELL DEVELOPED AND POSTERIORLY EXTENDING

1. REDUCED

**80.** **VENTROMEDIAN PROCESS OF METATARSAL V:**

0. PRESENT

1. ABSENT

**81.** **METATARSAL V CUBOID FACET:**

0. SHALLOW AND TRANSVERSELY ELONGATE

1. DEEP AND CUP-SHAPED

**82.** **PEDAL DIGIT IV PROXIMAL AND MEDIAL PHALANGES:**

0. NOT COMPRESSED

1. DORSOVENTRALLY COMPRESSED

**83.** **CRUCIATE FOSSA OF PEDAL DIGIT IV PROXIMAL PHALANX:**

0. DEEP AND NARROW

1. SHALLOW AND BROAD

**84.** **CROSSED SESAMOID AND OBLIQUE SESAMOID LIGAMENT SCARS OF PEDAL DIGIT IV PROXIMAL PHALANX:**

0. WEAKLY DEVELOPED

1. WELL DEVELOPED

**85.** **UNGUAL PROCESS OF PEDAL DIGIT IV DISTAL PHALANX:**

0. TAPERING AND POINTED OR DISTINCTLY CLEFT

1. ROUNDED AND HOOF LIKE

**86.** **POSTERIOR SURFACE OF FEMORAL SHAFT:**

0. MIDLINE BOSS VERY WEAKLY DEVELOPED OR ABSENT

1. BEARING PROMINENT MIDLINE BOSS

**87.** **CONDITION OF FEMORAL HEAD:**

0. SPHERICAL WITH CIRCULAR SECTION AND ORIENTED OBLIQUE TO LONG AXIS OF SHAFT

1. WITH DOMED SEMICIRCULAR SECTION ORIENTED PERPENDICULAR TO LONG AXIS OF SHAFT

**88.** **DISTAL PORTION OF FIBULA SHAFT:**

0. D-SHAPED IN CROSS SECTION

1. CRESCENTIC IN CROSS SECTION

**89.** **TIBIA SHAFT:**

0. STRAIGHT OR SLIGHTLY CURVED IN ANTEROPOSTERIOR VIEW

1. SINUOUS IN ANTEROPOSTERIOR VIEW

**90.** **ACROMION PROCESS OF SCAPULA:**

0. WELL DEVELOPED AND ANTERODORSALLY PRODUCED

1. POORLY DEVELOPED AND ROUNDED

**91.** **SUPRASPINOUS FOSSA OF SCAPULA:**

0. ANTEROPOSTERIORLY ELONGATE AND SUBEQUAL IN LENGTH TO INFRASPINOUS FOSSA

1. ANTEROPOSTERIORLY SHORTENED RELATIVE TO INFRASPINOUS FOSSA

**92.** **CORACOID PROCESS OF SCAPULA:**

0. LARGE WITH NECK

1. REDUCED WITHOUT NECK

**93.** **SUPINATOR CREST OF HUMERUS:**

0. WELL DEVELOPED AND TRANSVERSELY BROAD WITH DISTINCTLY POINTED APEX

1. REDUCED AND TRANSVERSELY NARROW WITH ROUNDED APEX

**94.** **DELTOID CREST OF HUMERUS:**

0. NOT UNIFORM IN HEIGHT WITH DISTAL EXTREMITY FORMING A STEEP INCLINE THAT MAY BEAR A RUGOSE BOSS AT ITS APEX

1. UNIFORM IN HEIGHT ALONG ITS LENGTH WITH DISTAL EXTREMITY FORMING A SHALLOW INCLINE

**95.** **LATERAL DELTOID RIDGE OF HUMERUS:**

0. POORLY DEVELOPED

1. WELL DEVELOPED AND PROMINENT

**96.** **ENTEPICONDYLE OF HUMERUS:**

0. SEPARATED FROM TROCHLEA BY A DISTINCT GROOVE

1. CLOSELY ABUTS TROCHLEA

**97.** **CORONAL DEPRESSION OF HUMERUS:**

0. SHALLOWLY EXCAVATED AND WEAKLY DELINEATED

1. DEEPLY EXCAVATED AND STRONGLY DELINEATED

**98.** **CAPITELLUM AND TROCHLEA OF HUMERUS:**

0. DISTINCTLY UNEQUAL IN SIZE WITH CAPITELLUM LARGER THAN TROCHLEA

1. SUBEQUAL IN SIZE

**99.** **SHAFT OF ULNA:**

0. STRAIGHT VENTRAL-EDGE PROFILE

1. DISTINCTLY SINUOUS VENTRAL-EDGE PROFILE

**100.** **OLECRANON PROCESS OF ULNA:**

0. DORSOVENTRALLY TALL WITH DISTINCT TRANSVERSE DORSAL CREST

1. DORSOVENTRALLY SHORT AND ROUNDED IN LATERAL VIEW

**101.** **DISTAL APEX OF ILIUM:**

0. TRANSVERSELY NARROWED BY POOR CONTRIBUTION OF DISTAL END OF ILIAC CREST

1. TRANSVERSELY BROADENED BY CONTRIBUTION OF THE RAISED DISTAL END OF THE ILIAC CREST

**102.** **ILIOPECTINEAL PROCESS OF ILIUM:**

0. REDUCED AND RESTRICTED TO A SLIGHT RIDGE

1. PROMINENT AND SUBCYLINDRICAL

**103.** **VENTRAL PROFILE OF ANTERIOR LUMBAR CENTRA:**

0. SMOOTH AND ROUNDED

1. BEARS DISTINCT MEDIAN KEEL

**104.** **CAUDAL CENTRA:**

0. CYLINDRICAL WITH SUBCIRCULAR CROSS SECTION

1. MARKEDLY DORSOVENTRALLY COMPRESSED WITH OVOID CROSS SECTION

**105.** **VENTRAL MARGIN OF MANDIBLE:**

0. CONVEX (ANTERIORMANDIBLE STEEPLY INCLINED)

1. STRAIGHT (ANTERIOR MANDIBLESHALLOWLY INCLINED)

**106.** **P_3_ TRANSCRISTIDS:**

0. ABSENT

1. COARSE, DEEPLY INCISED

2. FINE, SHALLOWLY INCISED

**107.** **P_3_ TRANSCRISTID NUMBER:**

0. TRANSCRISTAE ABSENT

1. ORLESS

2. BETWEEN 5 AND 10

3. 10 OR MORE

**108.** **LOWER MOLAR OCCLUSAL OUTLINE:**

0. RECTANGULAR (LENGTH ATLEAST 0.3 > WIDTH)

1. SQUARE (SUB-EQUAL LENGTH/WIDTH)
